# Electron‐Transfer and Hydride‐Transfer Pathways in the Stoltz–Grubbs Reducing System (KO*t*Bu/Et_3_SiH)

**DOI:** 10.1002/anie.201707914

**Published:** 2017-10-02

**Authors:** Andrew J. Smith, Allan Young, Simon Rohrbach, Erin F. O'Connor, Mark Allison, Hong‐Shuang Wang, Darren L. Poole, Tell Tuttle, John A. Murphy

**Affiliations:** ^1^ Department of Pure and Applied Chemistry University of Strathclyde 295 Cathedral Street Glasgow G1 1XL UK; ^2^ Flexible Discovery Unit GlaxoSmithKline Medicines Research Centre Gunnels Wood Road Stevenage SG1 2NY UK

**Keywords:** density-functional calculations, electron transfer, hydrides, reaction mechanisms, silicon

## Abstract

Recent studies by Stoltz, Grubbs et al. have shown that triethylsilane and potassium *tert*‐butoxide react to form a highly attractive and versatile system that shows (reversible) silylation of arenes and heteroarenes as well as reductive cleavage of C−O bonds in aryl ethers and C−S bonds in aryl thioethers. Their extensive mechanistic studies indicate a complex network of reactions with a number of possible intermediates and mechanisms, but their reactions likely feature silyl radicals undergoing addition reactions and S_H_2 reactions. This paper focuses on the same system, but through computational and experimental studies, reports complementary facets of its chemistry based on a) single‐electron transfer (SET), and b) hydride delivery reactions to arenes.

Recently, Stoltz, Grubbs et al.^1^ have discovered a simple and elegant system comprising Et_3_SiH (**2**) and KO*t*Bu which achieves a number of remarkable reactions: 1) converting arenes and heteroarenes, and their alkylated counterparts, into silyl‐substituted products, often with excellent regiocontrol1a–1c (e.g. **1**→**3**; Scheme 1); 2) achieving reductive C−S bond cleavage in aryl thioethers (e.g. **4**→**5**) in a reaction which has potential importance in removing sulfur traces from hydrocarbon fuels;1d 3) triggering reductive C−O bond cleavage in aryl ethers (e.g. **6**→**7**) in a reaction with potential applications to controlled lignin degradation.1a,1d A number of intermediates likely arise from reaction of these two reagents, and spectroscopic evidence has resulted in informed proposals being made for their structures. These reactions have proved puzzling, but a recent coordinated study by synthetic, mechanistic, and computational chemists has allowed significant advances to be made.1e,1f The conclusions are: 1) the combination of Et_3_SiH and KO*t*Bu leads to triethylsilyl radicals which have a major role to play in the reductive cleavage of the C−O and C−S bonds,1d 2) triethylsilyl radicals are also likely to be involved in the silylation reactions, although nonradical routes to the silylation have also been considered in depth and may also play a central role.1e,1f The mechanistic details are not fully in place, for example, on how formation of the silyl radicals occurs, but rational working hypotheses have been advanced.1e

**Scheme 1:**
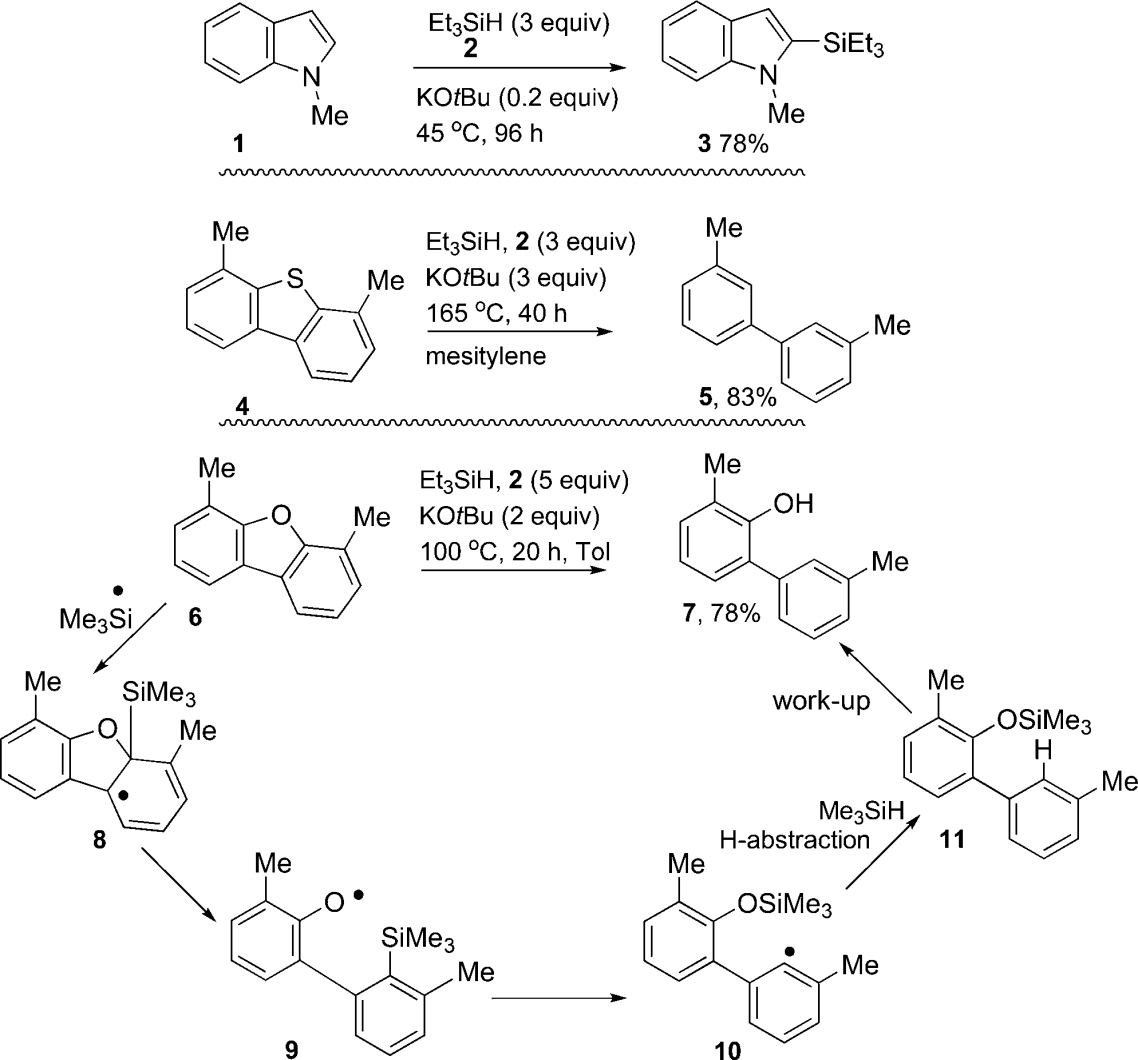
Selected transformations of the KO*t*Bu/Et_3_SiH system.^1^

We had wondered if single‐electron transfer mechanisms were playing a significant role in some of these reactions, notably for the cleavage of C−O and C−S bonds. An early suggestion1a mentioned pentavalent silicates (e.g. **13 b**; see Scheme 2) as reagents that were likely involved in the C−O cleavage, but the more recent computational studies on the substrates **4** and **6** instead support an alternative mechanism.1d In this regard, Scheme 1 shows *ipso* addition to the carbon atom of the C−O bond by triethylsilyl radicals, followed by C−O bond cleavage in conversion of **6** into **7**.

Our recent interest in reductive chemistry carried out by reactions involving KO*t*Bu attracted us to this area.^2^ Studies mentioned above1e suggest that the reactive species produced could include the radical anion **12 b** (Scheme 2) and the silicate anion **13 b**.1a,1e Because of their subsequent importance in this paper, we mention here that the radical anions **12** may be formed in a number of ways, two of which are shown (inset) in Scheme 2 (see Figure 14 in Ref. 1e for an additional route). For these studies, we used the computationally less costly trimethylsilyl group instead of the triethylsilyl group.1d,1e To these, we add the triethylsilyl anion **14 b** as another putative intermediate. At first sight, these compounds are potentially excellent electron donors, although, as will be seen below, computational chemistry is very helpful in eliminating species and mechanisms which are unlikely to contribute. In recent years, we have reported on many highly reducing organic electron donors that demonstrate remarkable behavior.^3^ We were therefore keen to test the KO*t*Bu/Et_3_SiH system for evidence of single‐electron transfer (SET) activity and, if found, to calibrate the system's reactivity.

**Scheme 2:**
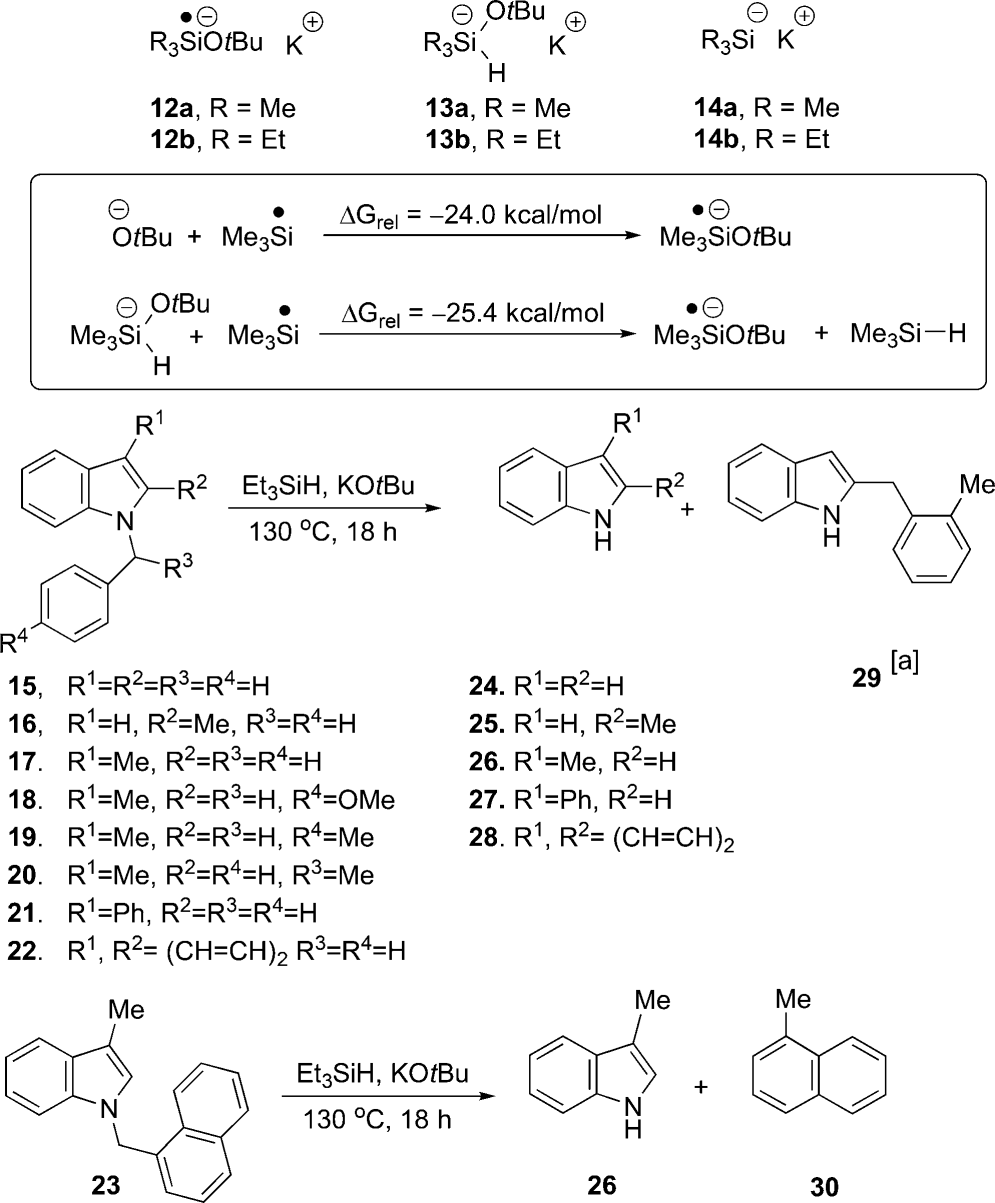
Indole‐based substrates as probes of electron‐transfer activity. [a] See the Supporting Information for a discussion of the mechanism of formation of this compound.

A literature search reveals that *N*‐benzylindole substrates are reductively cleaved to indoles and toluenes with two reagents—both involving electron transfer. The first uses Birch chemistry^4^ and the second uses low‐valent titanium reagents.^5^ Accordingly, we prepared a range of *N*‐benzylindole substrates (**15**–**23**; Scheme 2), to test for cleavage with silane and *tert*‐butoxide, and the outcomes are shown in Table 1. In each case, reactions afforded the debenzylated products, while blank reactions (no silane) led to excellent recovery of starting materials. The examples **15**–**22** also afforded volatile products from the benzyl unit. To counteract this, the naphthylmethyl substrate **23** was subjected to the reaction and afforded 1‐methylnaphthalene (**30**), in addition to 3‐methylindole (**26**), and recovered **23** (entry 18).

**Table 1:** Cleavage of benzyl groups from indole derivatives.

Entry	Substrate	Silane (3 or 0 equiv)	Base (3 equiv)	Yield [%]	
				Product	Recovered Substrate
1	**15**	Et_3_SiH	KO*t*Bu	**24** (29)	–
2	**15**	‐(blank)‐	KO*t*Bu	–	(85)
3	**16**	Et_3_SiH	KO*t*Bu	**25** (49) + **29** (15)	–
4	**16**	‐(blank)‐	KO*t*Bu	–	(99)
5	**17**	Et_3_SiH	KO*t*Bu	**26** (73)	–
6	**17**	Et_3_SiH	NaO*t*Bu^[a]^	–	(98)
7	**17**	‐(blank)‐	KO*t*Bu	–	(88)
8	**18**	Et_3_SiH	KO*t*Bu	**26** (76)	–
9	**18**	‐(blank)‐	KO*t*Bu	–	(98)
10	**19**	Et_3_SiH	KO*t*Bu	**26** (63)	Trace
11	**19**	‐(blank)‐	KO*t*Bu	–	(86)
12	**20**	Et_3_SiH	KO*t*Bu	**26** (47)	trace
13	**20**	‐(blank)‐	KO*t*Bu	–	(93)
14	**21**	Et_3_SiH	KO*t*Bu	**27** (80)	–
15	**21**	‐(blank)‐	KO*t*Bu	–	(100)
16	**22**	Et_3_SiH	KO*t*Bu	**28** (57)	(26)
17	**22**	‐(blank)‐	KO*t*Bu	–	(99)
18	**23**	Et_3_SiH	KO*t*Bu	**26** (55) + **30** (23)	(23)
19	**23**	‐(blank)‐	KO*t*Bu	–	(88)

Yields of products and recovered substrates are those for the isolated compounds. [a] As in Ref. 1, NaO*t*Bu is not a successful substitute for KO*t*Bu.

To understand the site of electron transfer in these reactions, we modelled the formation and reaction of two radical anions—those arising by electron transfer to the indole **17** and carbazole **22**. In both cases (Figure 1), the SOMO showed spin density on the heterocycle, rather than on the benzyl group. These data is consistent with the greater delocalization available in either the bicyclic or tricyclic heterocycle for the transferred electron.

**Figure 1:**
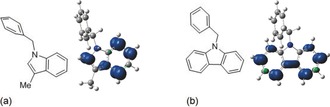
Representations of the spin density of the SOMO of the radical anion of *N*‐benzyl‐3‐methylindole **17** (a) and *N*‐benzylcarbazole **22** (b). Geometry optimizations and frequency calculations were carried out in Gaussian^13^ at M062X/6‐31++G(d,p) level of theory,^14^, ^15^ with solvation modelled implicitly using the C‐PCM model^16^ (For full computational details, see the Supporting Information).

We now use computational methods to compare the cleavage of the *N*‐benzyl group of **15** by an SET mechanism (Table 2) with the three potential electron donors **12 a**–**14 a**. Here it is seen that electron transfer from **12 a** to **15** is almost barrierless and is exergonic (entry 1; the scheme also shows facile fragmentation of the radical anion **31**), while the electron‐transfer reactions from **13 a** and **14 a** (entries 2 and 3) show prohibitive energy profiles.

**Table 2:** Energy profiles for candidate electron transfers to **15**. 
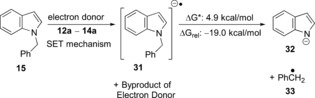

Entry	Electron donor		Energy profile [kcal mol^−1^]	Byproduct of electron donor	Byproduct of electron donor
1	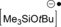	**12 a**	ΔG*=0.3 Δ*G*_rel_=−8.1	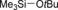	**34**
2	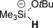	**13 a**	ΔG*=53.6 Δ*G*_rel_=49.4	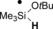	**35**
3	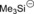	**14 a**	ΔG*=44.8 Δ*G*_rel_=38.7	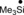	**36**

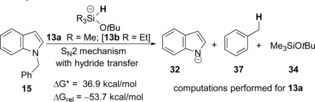


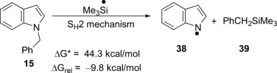

We also tested energy profiles for the debenzylation reaction with two possible competing pathways (Table 2; lower panels). The first of these recognizes that **13 a** could be a very powerful hydride‐transfer agent and might facilitate an S_N_2 reaction, although an unusual one, at the benzylic carbon center. However, transfer of hydride from **13 a** to **15** shows a barrier of 36.9 kcal mol^−1^ for the benzyl cleavage, and so this type of reaction will not occur under our reaction conditions in the laboratory. The second competing reaction type would involve an S_H_2 reaction by a R_3_Si radical at the benzylic carbon center. This path would also be an unexpected reaction, as radical displacements at tetrahedral carbon centers are almost unknown, and indeed the kinetic barrier (44.3 kcal mol^−1^) is again insurmountable. From these results, SET from **12 a** is overwhelmingly the most likely of the computed candidate mechanisms for benzyl group cleavage. In effect, cleavage occurred to afford *N*‐methylaniline, **41**, which was converted into the more easily isolated **42** following acetylation (56 % over 2 steps; Scheme 3). When the reaction was repeated, but in the absence of Et_3_SiH, no cleavage was observed, with the starting material **40** recovered (97 %). We next varied the protecting group on our indole substrates from benzyl to allyl. Given that the computational results showed electron transfer to the indole group in the substrates **17** and **22**, rather than to the benzyl group, then the reagent should also to be able to cleave *N*‐allylindoles by an SET mechanism, because of the stabilization of the allyl radical leaving group.^6^ Accordingly, the substrates **43** and **45** were prepared. The indole products **26** and **46** were indeed formed from these substrates (35 % and 33 % respectively). The low yields may indicate the wealth of alternative reactions open to this reagent system. Indeed, a second product was isolated from the reaction of **43**, namely *o*‐isopropylaniline (**44**; 18 %), although we have not explored the mechanism of its formation as yet. It was clear that the KO*t*Bu/Et_3_SiH system is a more than competent electron‐donating system.

**Scheme 3:**
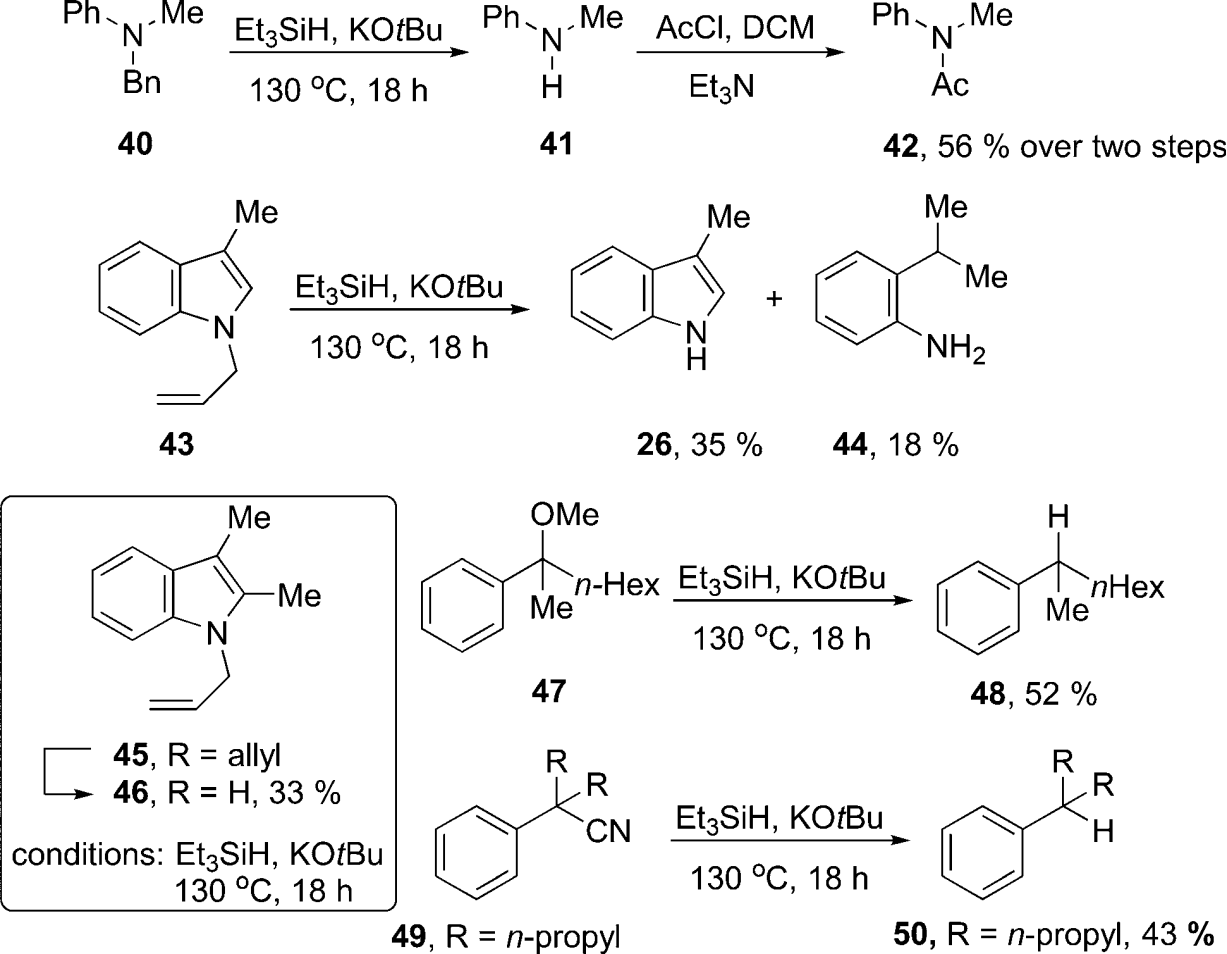
Reductive cleavage induced by the Et_3_SiH/KO*t*Bu system.

In a more challenging probe for electron‐transfer potency, we subjected the benzyl methyl ether **47** to reduction by this system (Scheme 3). A close analogue of this substrate had proven a very tough substrate in previous studies.3h It did not undergo fragmentation until two electrons had been transferred. In this case, the reduced product **48** was produced in 52 % yield [a blank reaction afforded recovered starting material exclusively (62 %)]. Additionally, subjecting the nitrile **49** 7 to the reaction afforded the hydrocarbon **50** as the sole product, consistent with electron transfer followed by loss of cyanide anion.

We calculated the oxidation potential of **12 a** 8 to be E=−3.74 V vs. SCE (MeCN). This potential makes it much more powerful than alkali metals. Such a powerful electron donor should provide a good probe for the Marcus inverted region of SET reactions with substrates that show low reorganization energies, (e.g. polycyclic arenes).^9^ Stoltz, Grubbs et al. reported1d small amounts of partially reduced arenes from reduction of naphthalenes. In our hands, and in the presence of excess of KO*t*Bu/Et_3_SiH, anthracene, phenanthrene, and naphthalene all afforded significant amounts of their dihydro counterparts (Scheme 4). These compounds would be expected products from Birch‐type electron‐transfer processes, but to probe the mechanism we undertook computational studies of electron transfer from **12 a** to the hydrocarbons **51**–**53** to yield the corresponding radical anions **60**–**62**. (Table 3) Here, the expected normal order of reactivity is **51**>**52**>**53**.^10^ This order is also reflected in the Δ*G*_rel_ values shown in Table 3. However, the reverse pattern is seen for the ΔG* values. SET to **51** from the radical anion **12 a** shows an extraordinary barrier of 90 kcal mol^−1^,^11^ while reduction of **52** and **53** show progressively lower barriers; if this can be verified by detailed experimental studies, it will be a very rare intermolecular ground‐state illustration of the Marcus inverted region, (stronger driving force leads to retarded electron transfer).

**Scheme 4:**
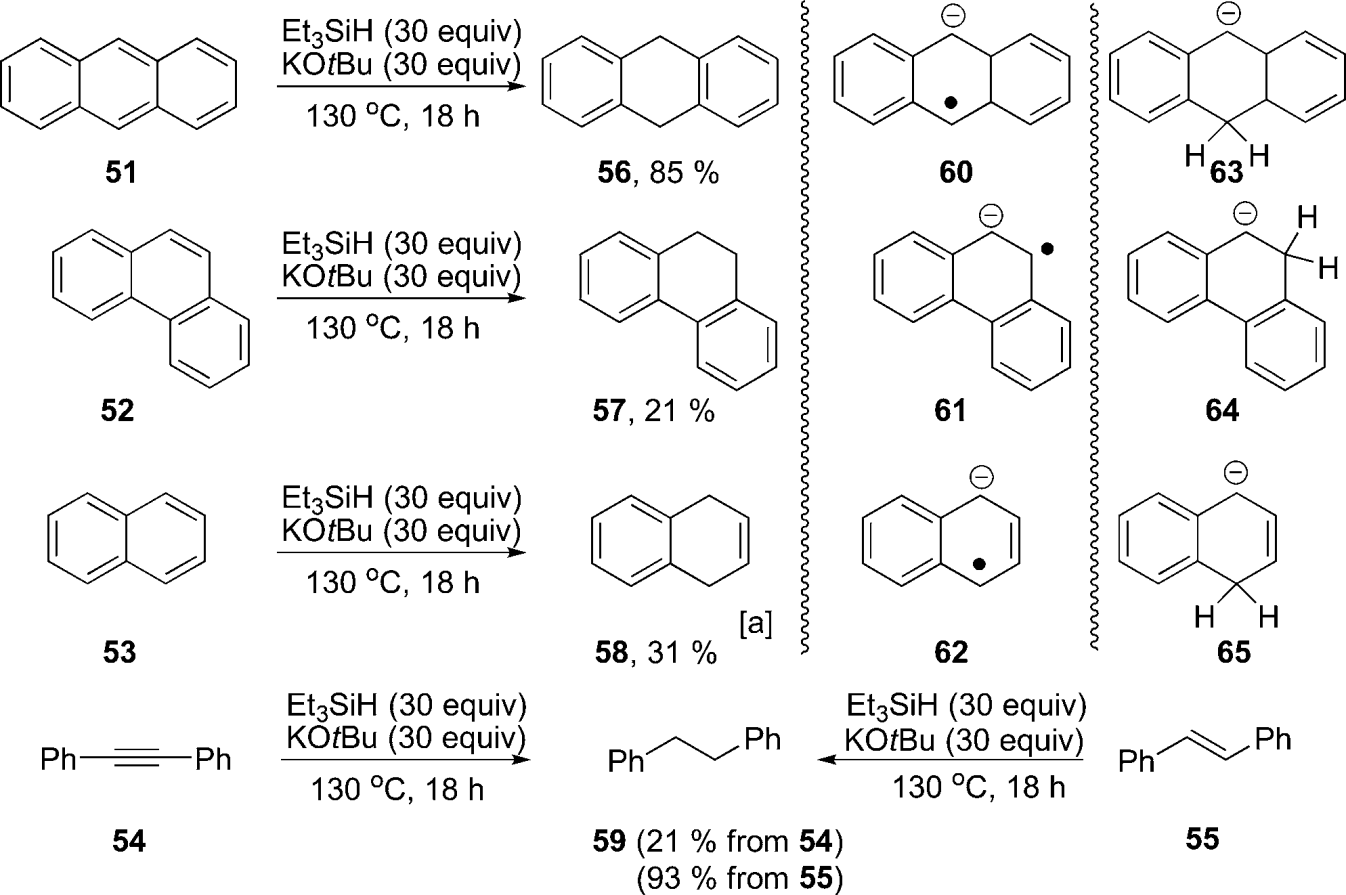
Reductions of polycylic arenes by KO*t*Bu/Et_3_SiH. [a] Yield determined by NMR spectroscopy.

**Table 3:** Energy profiles: SET from **12 a**.

Substrate	Energy profile [kcal mol^−1^]	Radical anion product
**51**	ΔG*: 90.0; Δ*G*_rel_: −37.8	**60**
**52**	ΔG*: 28.3; Δ*G*_rel_: −25.0	**61**
**53**	ΔG*: 25.7; Δ*G*_rel_: −22.3	**62**

In comparison, hydride transfer from **13 a** to afford the corresponding anions **63**–**65** featured low barriers and favorable thermodynamics (Table 4). At least for the reduction of anthracene, hydride transfer from **13 a** is indeed likely to occur. With the other substrates, hydride‐transfer reactions again show lower barriers than electron transfer from **12 a** and this will of course be modulated by the concentration of the reducing species present. Finally, the alkyne **54** and stilbene **55** were reacted and gave (PhCH_2_)_2_ **59** as the sole product (21 and 93 % respectively; Scheme 4).^12^

**Table 4:** Energy profiles: Hydride transfer from **13 a**.

Substrate	Energy profile [kcal mol^−1^]	Anionic product
**51**	ΔG*: 16.7; Δ*G*_rel_: −29.4	**63**
**52**	ΔG*: 20.0; Δ*G*_rel_: −14.8	**64**
**53**	ΔG*: 21.7; Δ*G*_rel_: −13.2	**65**

In summary, the KO*t*Bu/Et_3_SiH system provides access to a broad range of mechanisms for reductive chemistry, now including electron transfer and hydride delivery to arenes. The electron‐donor **12 b** is identified as a uniquely powerful agent.

## Conflict of interest

The authors declare no conflict of interest.

## Supporting information

As a service to our authors and readers, this journal provides supporting information supplied by the authors. Such materials are peer reviewed and may be re‐organized for online delivery, but are not copy‐edited or typeset. Technical support issues arising from supporting information (other than missing files) should be addressed to the authors.

Supplementary
