# Supplementary material for: Electron‐Transfer and Hydride‐Transfer Pathways in the Stoltz–Grubbs Reducing System (KOtBu/Et3SiH)
Source: Angew Chem Int Ed Engl. 2017 Oct 2;56(44):13747–51. doi: 10.1002/anie.201707914 (PMC5656899; doi:10.1002/anie.201707914)
Supplement: Supplementary file 1 — Supplementary [file ANIE-56-13747-s001.pdf]

Angewandte Chemie
Eine Zeitschrift der Gesellschaft Deutscher Chemiker

Supporting Information

Electron-Transfer and Hydride-Transfer Pathways in the Stoltz–Grubbs Reducing System ($KO\textit{t}Bu/Et_3SiH$)

Andrew J. Smith, Allan Young, Simon Rohrbach, Erin F. O'Connor, Mark Allison, Hong-Shuang Wang, Darren L. Poole, Tell Tuttle, and John A. Murphy**

anie_201707914_sm_miscellaneous_information.pdf

Contents

General Procedures - 6 -
- General Experimental - 6 -
- Computational Methods - 6 -
- Electron Transfer Methodology - 6 -
- Calculated Reduction and Oxidation Potentials - 7 -
- General Procedure A - Benzylation of Indoles[9] - 8 -
- General Procedure B - Allylation of Indoles[9] - 8 -
- General Procedure C - Debenzylation/Deallylation of Indole Derivatives - 8 -
- General Procedure D - Blank Reactions in the Absence of Triethylsilane - 9 -
- General Procedure E – Reductive Cleavage of Ethers - 9 -
- General Procedure F - Reduction of Arenes - 9 -
- Determination of Product Yields by ^{1}H-NMR - 9 -

Substrates - 10 -
- 1-Benzyl-1H-indole (**15**)[9] - 10 -
- 1-Benzyl-2-methyl-1H-indole (**16**)[9] - 10 -
- 1-Benzyl-3-methyl-1H-indole (**17**)[11] - 11 -
- 1-(4-Methoxybenzyl)-3-methyl-1H-indole (**18**) - 11 -
- 3-Methyl-1-(4-methylbenzyl)-1H-indole (**19**) - 12 -
- 3-Methyl-1-(1-phenylethyl)-1H-indole (**20**) - 12 -
- 3-Phenyl-1H-indole (**27**)[13] - 13 -
- 1-Benzyl-3-phenyl-1H-indole (**21**)[15] - 13 -
- 9-Benzyl-9H-carbazole (**22**)[17] - 14 -
- 3-Methyl-1-(naphthalen-1-ylmethyl)-1H-indole (**23**) - 14 -
- N-Benzyl-N-methylaniline (**40**)[18] - 15 -
- 1-Allyl-3-methyl-1H-indole (**43**)[19] - 15 -
- 1-Allyl-2,3-dimethyl-1H-indole (**45**) - 16 -
- (2-Methoxyoctan-2-yl)benzene (**47**)[20] - 16 -
- 2-Phenyl-2-propylpentanenitrile (**49**) - 17 -
- 1,2-Diphenylethyne (**54**)[21] - 17 -
- Literature synthesis of 1,4-Dihydronaphthalene (**58**)[23] - 18 -

Products - 18 -
- 1H-Indole (**24**)[24] - 18 -
- 2-Methyl-1H-indole (**25**)[26] and 2-(2-methylbenzyl)-1H-indole (**29**)[27] - 19 -
- 3-Methyl-1H-indole (**26**)[28] - 19 -
- 9H-Carbazole (**28**)[29] - 20 -
- 1-Methylnaphthalene (**30**)[31] - 20 -

N-methyl-N-phenylacetamide (**42**)[32] .... - 21 -
2-Isopropylaniline (**44**)[33] .... - 21 -
2,3-Dimethyl-1H-indole (**46**)[34] .... - 22 -
Octan-2-ylbenzene (**48**)[36] .... - 22 -
4-Phenylheptane (**50**) .... - 23 -
9,10-Dihydroanthracene (**56**)[37] .... - 23 -
9,10-Dihydrophenanthrene (**57**)[39] .... - 24 -
1,2-Diphenylethane (**59**)[40] .... - 24 -
Suggested Mechanism for the Formation of **29** .... - 24 -
References .... - 25 -
Spectra .... - 29 -
Substrates .... - 29 -
1-Benzyl-1H-indole (**15**) .... - 29 -
1-Benzyl-2-methyl-1H-indole (**16**) .... - 30 -
1-Benzyl-3-methyl-1H-indole (**17**) .... - 31 -
1-(4-Methoxybenzyl)-3-methyl-1H-indole (**18**) .... - 32 -
3-Methyl-1-(4-methylbenzyl)-1H-indole (**19**) .... - 33 -
3-Methyl-1-(1-phenylethyl)-1H-indole (**20**) .... - 34 -
3-Phenyl-1H-indole (**27**) .... - 35 -
1-Benzyl-3-phenyl-1H-indole (**21**) .... - 36 -
9-Benzyl-9H-carbazole (**22**) .... - 37 -
3-Methyl-1-(naphthalen-1-ylmethyl)-1H-indole (**23**) .... - 38 -
N-Benzyl-N-methylaniline (**40**) .... - 39 -
1-Allyl-3-methyl-1H-indole (**43**) .... - 40 -
1-Allyl-2,3-dimethyl-1H-indole (**45**) .... - 41 -
2-Phenyloctan-2-ol .... - 42 -
(2-Methoxyoctan-2-yl)benzene (**47**) .... - 43 -
2-Phenyl-2-propylpentanenitrile (**49**) .... - 44 -
1,2-Diphenylethyne (**54**) .... - 45 -
1,4-Dihydronaphthalene (**58**) .... - 46 -
Products .... - 47 -
1H-indole (**24**) .... - 47 -
2-Methyl-1H-indole (**25**) .... - 48 -
3-Methyl-1H-indole (**26**) .... - 49 -
9H-carbazole (**28**) .... - 50 -
2-(2-Methylbenzyl)-1H-indole (**29**) .... - 51 -
1-Methylnaphthalene (**30**) .... - 52 -

N-methyl-*N*-phenylacetamide (**42**) ... - 53 -
2-Isopropylaniline (**44**) ... - 54 -
2,3-Dimethyl-1H-indole (**46**) ... - 55 -
Octan-2-ylbenzene (**48**) ... - 56 -
4-Phenylheptane (**50**) ... - 57 -
9,10-Dihydroanthracene (**56**) ... - 58 -
9,10-Dihydrophenanthrene (**57**) ... - 59 -
1,2-Diphenylethane (**59**) ... - 60 -
Computational Chemistry - XYZ Coordinates ... - 61 -
$[Me_3SiO^tBu]^{\bullet-}$ ... - 61 -
$[Me_3SiO^tBu]^{\bullet-}$ (Acetonitrile as Solvent) ... - 61 -
$[Me_3SiO^tBu]^{\bullet-}$ Optimised Geometry - Single Point as Neutral Singlet ... - 62 -
Me_3SiO^tBu ... - 63 -
Me_3SiO^tBu (Acetonitrile as Solvent) ... - 64 -
Me_3SiO^tBu Optimised Geometry - Single Point as Radical Anion ... - 64 -
Me_3SiH ... - 65 -
O^tBu Anion ... - 66 -
Me_3SiH + O^tBu Anion Transition State ... - 66 -
$[(Me)_3(H)SiO^tBu]^-$ ... - 67 -
$[(Me)_3(H)SiO^tBu]^-$ Optimised Geometry - Single Point as Radical ... - 68 -
$[(Me)_3(H)SiO^tBu]^\bullet$ ... - 68 -
$[(Me)_3(H)SiO^tBu]^\bullet$ Optimised Geometry - Single Point as Anion ... - 69 -
Me_3SiH + O^tBu Deprotonation Transition State ... - 70 -
HO^tBu ... - 71 -
$[SiMe_3]^-$ ... - 72 -
$[SiMe_3]^-$ Optimised Geometry - Single Point as Radical ... - 72 -
$[SiMe_3]^\bullet$ ... - 73 -
$[SiMe_3]^\bullet$ Optimised Geometry - Single Point as Anion ... - 73 -
N-Benzylcarbazole ... - 74 -
N-Benzylcarbazole Radical Anion ... - 75 -
N-Benzylindole ... - 76 -
N-Benzylindole Optimised Geometry - Single Point as Radical Anion ... - 77 -
N-Benzylindole Radical Anion ... - 78 -
N-Benzylindole Radical Anion Optimised Geometry - Single Point as Neutral ... - 79 -
Indole Anion ... - 79 -
Toluene ... - 80 -
N-Benzylindole + $[(Me)_3(H)SiO^tBu]^-$ Transition State ... - 80 -

N-Benzylindole Radical Anion Fragmentation Transition State .... - 82 -
Toluene Radical .... - 83 -
N-Benzylindole + $[SiMe_3]^{\bullet}$ Transition State .... - 83 -
$PhCH_2SiMe_3$ .... - 84 -
Indole Radical .... - 85 -
3-Methyl-N-benzylindole .... - 86 -
3-Methyl-N-benzylindole Radical Anion .... - 87 -
Anthracene .... - 88 -
Anthracene (Acetonitrile as solvent) .... - 88 -
Anthracene Optimised Geometry - Single Point as Radical Anion .... - 89 -
Anthracene Radical Anion .... - 90 -
Anthracene Radical Anion (Acetonitrile as solvent) .... - 90 -
Anthracene Radical Anion Optimised Geometry - Single Point as Neutral .... - 91 -
9,10-Dihydroanthracen-9-ide .... - 92 -
Anthracene + $[(Me)_3(H)SiO^tBu]^-$ Transition State .... - 93 -
Phenanthrene .... - 94 -
Phenanthrene (Acetonitrile as solvent) .... - 95 -
Phenanthrene Optimised Geometry - Single Point as Radical Anion .... - 95 -
Phenanthrene Radical Anion .... - 96 -
Phenanthrene Radical Anion (Acetonitrile as solvent) .... - 97 -
Phenanthrene Radical Anion Optimised Geometry - Single Point as Neutral .... - 97 -
9,10-Dihydrophenanthren-9-ide .... - 98 -
Phenanthrene + $[(Me)_3(H)SiO^tBu]^-$ Transition State .... - 99 -
Naphthalene .... - 100 -
Naphthalene (Acetonitrile as solvent) .... - 101 -
Naphthalene Optimised Geometry - Single Point as Radical Anion .... - 101 -
Naphthalene Radical Anion .... - 102 -
Naphthalene Radical Anion (Acetonitrile as solvent) .... - 102 -
Naphthalene Radical Anion Optimised Geometry - Single Point as Neutral .... - 103 -
1,4-Dihydronaphthalen-1-ide .... - 103 -
Naphthalene + $[(Me)_3(H)SiO^tBu]^-$ Transition State .... - 104 -

General Procedures

General Experimental

All reagents and solvents were obtained from commercial suppliers and were used without further purification. The glovebox was supplied by Innovative Technology Inc., USA, and the atmosphere used is nitrogen. Sodium hydride was supplied as a 60 % dispersion in mineral oil and was washed with hexane to remove this oil before use. Thin layer chromatography was carried out using Merck silica plates coated with fluorescent indicator UV254. These were analysed under 254 nm UV light. Flash chromatography was carried out using ZEOprep 60 HYD 40-63 µm silica gel. Fourier Transform Infra-Red (FTIR) spectra were obtained on a Shimadzu IRAffinity-1 machine. ^{1}H and ^{13}C NMR spectra were obtained on a Bruker DPX 400 spectrometer at 400 and 101 MHz, respectively. Chemical shifts are reported in ppm and coupling constants are reported in Hz, with $CDCl_3$ referenced at 7.27 (^{1}H) and 77.00 ppm (^{13}C). The following abbreviations are used for the multiplicities: s, singlet; d, doublet; t, triplet; q, quartet; spt, septet; m, multiplet; br, broad. High resolution mass spectrometry (HRMS) was performed at the University of Swansea in the EPSRC National Mass Spectrometry Centre. Accurate mass was obtained using LTQ Orbitrap XL using Atmospheric Pressure Chemical Ionisation (APCI) or High Resolution Nano-Electrospray (HNESP) using Electrospray Ionisation (ESI).

Computational Methods

Density Functional Theory (DFT) was used for the geometry optimisations of all reactants, transition states (TSs), intermediates and products. The final optimised geometries were characterised as minima or transition states by performing frequency calculations, which also enabled calculation of the zero-point energies (ZPE), enthalpies (H), entropies (S) and Gibbs free energies (G) at 298 K. All geometry optimisations and frequency calculations were performed in Gaussian09,[1] with the M06-2X functional[2] and the 6-31++G(d,p) basis set.[3] The effects of solvation were modelled implicitly using the conductor-like polarisable continuum model (CPCM),[4] with triethylamine (ε=2.3832) as solvent (unless otherwise specified). Since triethylsilane is not defined in Gaussian09, triethylamine was deemed the most suitable alternative, with a dielectric constant similar to that of triethylsilane (ε=2.323).[5]

Electron Transfer Methodology

In order to model a single electron transfer reaction computationally, Marcus-Hush Theory[6] is employed with the 4-point method of Nelsen et al.[7] allowing calculation the reorganisation energy (λ), ΔG_{rel} and ΔG^*. This method requires optimisation of the individual electron donor and acceptor species, before and after single electron transfer. Single point energy calculations are then performed on these optimised geometries using the charge and multiplicity of their other state in the electron transfer reaction.

Table SI1 Calculated Reorganisation Energy, Relative Free Energies and Activation Free Energies for Single Electron Transfer from **12a** to **15**, **51**, **52**, and **53**.

Substrate	Reorganisation Energy (λ, kcal/mole)	ΔG_{rel} (kcal/mole)	ΔG^* (kcal/mole)
**15**	5.4	-8.1	0.3
**51**	3.3	-37.8	90.0
**52**	3.9	-25.0	28.3
**53**	3.4	-22.3	25.7

Calculated Reduction and Oxidation Potentials

In order to calculate the reduction and oxidation potentials of the species in Table SI2, the method of Nicewicz et al. was utilised (Equation S1).[8]

$$E_{1/2}^{0,calc} = -\frac{(G_{298}[reduced] - G_{298}[oxidised])}{n_e F} - E_{1/2}^{0,SHE} + E_{1/2}^{0,SCE} \qquad \text{Equation S1}$$

Where n_e is the number of electrons transferred (here, $n_e = 1$ in all examples), F is the Faraday Constant (23.061 kcal mole^{-1} V^{-1}), $E_{1/2}^{0,SHE}$ is the absolute value for the standard hydrogen electrode (SHE, 4.281V), $E_{1/2}^{0,SCE}$ is the potential of the saturated calomel electrode (SCE) relative to SHE in acetonitrile (-0.141V), $G_{298}[reduced]$ and $G_{298}[oxidised]$ are the Gibbs free energies in acetonitrile obtained from DFT calculations. In order to allow determination of Gibbs free energies in acetonitrile, geometry optimisations and frequency calculations were conducted for each species using the parameters for acetonitrile (ε=35.688), whilst maintaining the previously described level of theory.

Table SI2 Calculated Reduction or Oxidation Potentials

Species	$E_{1/2}^{M06-2X}$(V, *vs*. SCE, in MeCN)
**12a**	-3.74 (Oxidation)
**51**	-1.96 (Reduction)
**52**	-2.50 (Reduction
**53**	-2.57 (Reduction)

General Procedure A - Benzylation of Indoles[9]

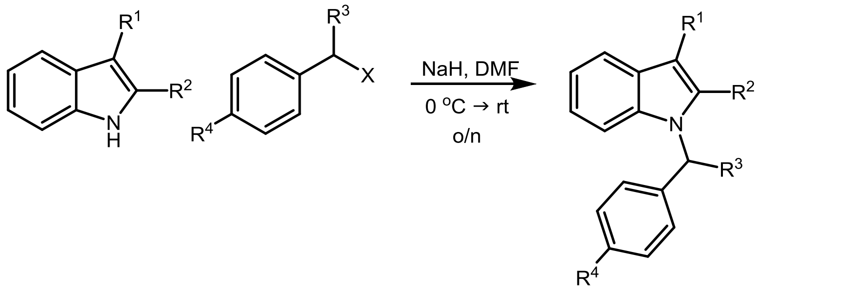

24. R^1=R^2=H
25. R^1=H, R^2=Me
26. R^1=Me, R^2=H
27. R^1=Ph, R^2=H
28. R^1, R^2= $(CH{=}CH)_2$

15. R^1=R^2=R^3=R^4=H
16. R^1=H, R^2=Me, R^3=R^4=H
17. R^1=Me, R^2=R^3=R^4=H
18. R^1=Me, R^2=R^3=H, R^4=OMe
19. R^1=Me, R^2=R^3=H, R^4=Me
20. R^1=Me, R^2=R^4=H, R^3=Me
21. R^1=Ph, R^2=R^3=R^4=H
22. R^1, R^2= $(CH{=}CH)_2$ R^3=R^4=H

To a stirred solution of sodium hydride (144 mg, 6.00 mmol, 1.2 equiv.) in DMF (5 mL) was added the appropriate indole (5.00 mmol, 1.0 equiv.) in DMF (5.0 mL) at 0 °C under nitrogen. The resulting mixture was stirred at room temperature for 30 min before addition of the appropriate benzyl halide (7.50 mmol, 1.5 equiv.) at 0 °C. The resulting mixture was stirred overnight at room temperature. The reaction mixture was then quenched with water and extracted into ethyl acetate. The combined organic layers were dried over Na_2SO_4, filtered and concentrated under reduced pressure.

General Procedure B - Allylation of Indoles[9]

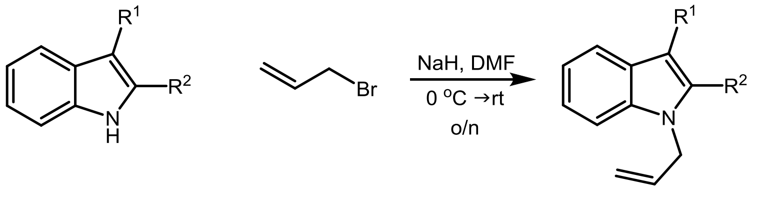

26. R^1=Me, R^2=H
46. R^1=R^2=Me

43. R^1=Me, R^2=H
45. R^1=R^2=Me

To a stirred solution of sodium hydride (144 mg, 6.00 mmol, 1.2 equiv.) in DMF (5.0 mL) was added the appropriate indole (5.00 mmol, 1.0 equiv.) in DMF (5.0 mL) at 0 °C under nitrogen. The resulting mixture was stirred at room temperature for 30 min before addition of allyl bromide (0.65 mL, 7.5 mmol, 1.5 equiv.) at 0 °C. The resulting mixture was stirred overnight at room temperature. The reaction mixture was then quenched with water and extracted into ethyl acetate. The combined organic layers were dried over Na_2SO_4, filtered and concentrated under reduced pressure.

General Procedure C - Debenzylation/Deallylation of Indole Derivatives

N-benzylindole derivative (0.50 mmol, 1.0 equiv.), triethylsilane (0.24 mL, 1.5 mmol, 3.0 equiv.) and potassium *tert*-butoxide (168 mg, 1.50 mmol, 3.0 equiv.) were sealed in a pressure tube in a glovebox under nitrogen. The tube was removed and heated at 130 °C for 18 h behind a safety shield. After cooling to room temperature, the mixture was diluted with water and extracted into diethyl ether. The

combined organic layers were dried over Na_2SO_4, filtered and concentrated under reduced pressure to yield the debenzylated indole derivative. Purification by column chromatography, as specified below, afforded the product.

General Procedure D - Blank Reactions in the Absence of Triethylsilane

N-benzylindole derivative (0.5 mmol, 1 equiv.) and potassium *tert*-butoxide (168 mg, 1.5 mmol, 3 equiv.) were sealed in a pressure tube in a glovebox under nitrogen. The tube was removed from the glove box and heated at 130 °C for 18 h behind a safety shield. After cooling to room temperature, the mixture was diluted with water and extracted into diethyl ether. The combined organic layers were dried over Na_2SO_4, filtered and concentrated under reduced pressure.

General Procedure E – Reductive Cleavage of Ethers

Substrate **47** (110 mg, 0.50 mmol, 1.0 equiv.), triethylsilane (0.24 mL, 1.5 mmol, 3.0 equiv.) and potassium *tert*-butoxide (168 mg, 1.50 mmol, 3.0 equiv.) were sealed in a pressure tube in a glovebox under nitrogen. The tube was removed and heated at 130 °C for 18 h behind a safety shield. After cooling to room temperature, the mixture was diluted with water and extracted into diethyl ether. The combined organic layers were dried over Na_2SO_4, filtered and concentrated under reduced pressure. Purification by column chromatography, as specified below, afforded the product.

General Procedure F - Reduction of Arenes

The reaction vessel was charged with the arene substrate (0.50 mmol, 1.0 equiv.). The reaction vessel was then brought inside the glovebox and triethylsilane (2.40 mL, 15.0 mmol, 30 equiv.) and potassium *tert*-butoxide (1.68 g, 15.0 mmol, 30 equiv.) were added in this sequence. The pressure tube was sealed and the reaction was heated to 130 °C for 18 h outside the glovebox. The reaction was carefully quenched (exothermic reaction, vigorous gas evolution) with water until all solid material was dissolved and no more gas evolved. The crude mixture was partitioned between water (20 mL) and dichloromethane (20 mL). The phases were separated and the aqueous phase was washed with dichloromethane (3 x 15 mL). The combined organic phases were back-extracted with water (20 mL), dried over $MgSO_4$ and carefully concentrated under reduced pressure (45 °C, >100 mbar, < 15 min). The reaction products were purified by flash column chromatography (hexane).

Determination of Product Yields by ^{1}H-NMR

In some special cases, product yields were determined by NMR as noted below. After the standard work-up an exactly measured amount of 1,3,5-trimethoxybenzene was added to the crude material. All material was dissolved in a small amount of chloroform-d_1 and the material was analysed by ^{1}H-NMR. Aliphatic signals of the compounds of interest were referenced against the methoxy signal of the internal standard, aromatic signals were referenced against the aromatic signals of the internal

standard. The signals of the compounds of interest were compared to the signals of an authentic sample.

Substrates

1-Benzyl-1*H*-indole (15)[9]

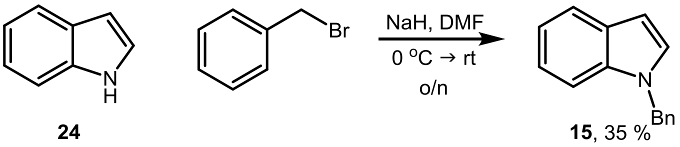

24

15, 35 %

Prepared according to the **General Procedure A - Benzylation of Indoles** from indole **24** (586 mg, 5.00 mmol, 1.0 equiv.) and benzyl bromide (0.89 mL, 7.5 mmol, 1.5 equiv.). Purification by distillation (200 °C, 21 mbar) afforded 1-benzyl-1*H*-indole **15** as a yellow oil (364 mg, 35%). **^{1}H-NMR** (400 MHz, $CDCl_3$) δ 5.35 (s, 2H, CH_2) 6.58 (dd, *J* = 3.3, 0.8 Hz, 1H, Ar*H*) 7.10 - 7.17 (m, 4H, Ar*H*) 7.20 (td, *J* = 7.0, 1.0 Hz, 1H, Ar*H*) 7.24 - 7.36 (m, 4H, Ar*H*) 7.68 (m, 1H, Ar*H*). **^{13}C-NMR** (101 MHz, $CDCl_3$) δ 50.0, 101.7, 109,7, 119.5, 121.0, 121.7, 126.8, 127.6, 128.2, 128.7, 136.3, 137.5. **ATR-IR** v_{max} (neat)/cm^{-1} 3025, 1604, 1511, 1454, 1316, 1182, 1013, 740, 715, *m/z* **(EI)**: 207.1 (87, $[M]^+$), 116.1 (11), 91.1 (100), 77.1 (3), 65.0 (17):

1-Benzyl-2-methyl-1*H*-indole (16)[9]

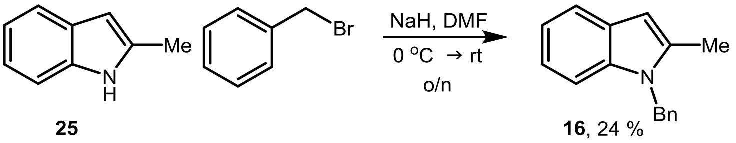

25

16, 24 %

Prepared according to the **General Procedure A - Benzylation of Indoles** from 2-methylindole **25** (655 mg, 5.00 mmol, 1.0 equiv.) and benzyl bromide (0.89 mL, 7.5 mmol, 1.5 equiv.). Purification by column chromatography (4:1 hexane: toluene) afforded 1-benzyl-2-methyl-1*H*-indole **16** as a white solid (266 mg, 24%). **Mp** = 42-44 °C (lit. mp = 47-48 °C).[10] **^{1}H-NMR** (400 MHz, $CDCl_3$) δ 2.41 (d, *J* = 0.8 Hz, 3H, CH_3), 5.34 (s, 2H, CH_2), 6.28 - 6.44 (m, 1H, Ar*H*), 7.01 (m, 2H, Ar*H*), 7.07 - 7.19 (m, 2H, Ar*H*), 7.20 - 7.34 (m, 4H, Ar*H*), 7.56 - 7.64 (m, 1H, Ar*H*). **^{13}C-NMR** (101 MHz, $CDCl_3$) δ 12.7, 46.4, 100.4, 109.2, 119.5, 119.7, 120.7, 126.0, 127.2, 128.2, 128.7, 136.7, 137.2, 137.9. **ATR-IR** v_{max} (neat)/cm^{-1} 3022, 2899, 1547, 1452, 1395, 1339, 1311, 1078, 1021, 801, 736, 721, 695. ***m/z* (EI):** 221.1 (90, $[M]^+$), 91.1 (100), 77.0 (12), 65.0 (20)

1-Benzyl-3-methyl-1*H*-indole (17)[11]

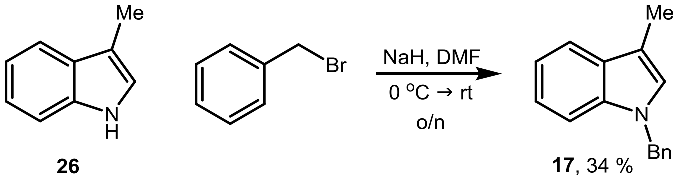

Prepared according to the **General Procedure A - Benzylation of Indoles** from 3-methylindole **26** (655 mg, 5.00 mmol, 1.0 equiv.) and benzyl bromide (0.89 mL, 7.5 mmol, 1.5 equiv.). Purification by column chromatography (9:1 hexane:diethyl ether), followed by recrystallisation from methanol afforded 1-benzyl-3-methyl-1*H*-indole **17** as a green solid (372 mg, 34%). **Mp** = 72-73 °C (lit. mp = 72-73 °C).[12] **^{1}H-NMR** (400 MHz, $CDCl_3$) δ 2.38 (d, *J* = 1.0 Hz, 3H, CH_3), 5.30 (s, 2H, CH_2), 6.93 (s, 1H, Ar*H*), 7.12 - 7.18 (m, 3H, Ar*H*), 7.21 (td, *J* = 7.8, 1.0 Hz, 1H, Ar*H*), 7.26 - 7.36 (m, 4H, Ar*H*), 7.63 (d, *J* = 7.8 Hz, 1H, Ar*H*); **^{13}C-NMR** (101 MHz, $CDCl_3$) δ 9.6, 49.8, 109.4, 110.8, 118.8, 119.0, 121.6, 125.8, 126.8, 127.5, 128.7, 128.9, 136.6, 137.9. **ATR-IR** ν_{max} (neat)/cm^{-1} 3042, 3029, 2927, 2912, 1614, 1586, 1462, 1467, 1439, 1359, 1331, 1181, 732, 697. ***m/z* (EI):** 221.1 (87, $[M]^+$), 130.1 (14), 91.0 (100), 77.0 (10), 65.0 (15)

1-(4-Methoxybenzyl)-3-methyl-1*H*-indole (18)

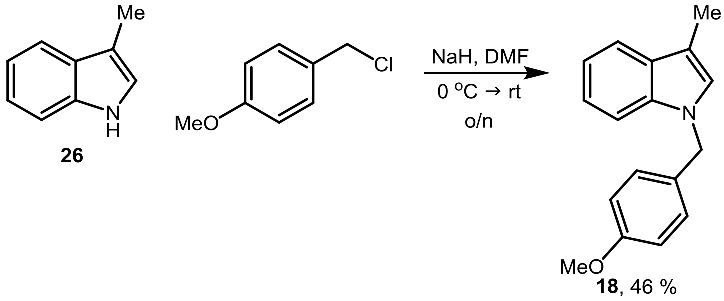

Prepared according to the **General Procedure A - Benzylation of Indoles** from 3-methylindole **26** (655 mg, 5.00 mmol, 1.0 equiv.) and 4-methoxybenzyl chloride (1.02 mL, 7.50 mmol, 1.5 equiv.). Purification by column chromatography (9:1 hexane:diethyl ether) afforded 1-(4-methoxybenzyl)-3-methyl-1*H*-indole **18** as a yellow solid (584 mg, 46 %). **Mp** = 45-48 °C. **^{1}H-NMR** (400 MHz, $CDCl_3$) δ 2.34 (d, *J* = 1.0 Hz, 3 H, CH_3), 3.79 (s, 3 H, OCH_3), 5.21 (s, 2 H, CH_2), 6.84 (d, *J* = 8.8 Hz, 2 H, Ar*H*), 6.88 (d, *J* = 1.0 Hz, 1 H, Ar*H*), 7.06 - 7.11 (m, 2 H, Ar*H*), 7.11 - 7.15 (m, 1 H, Ar*H*), 7.19 (td, *J* = 7.5, 1.3 Hz, 1 H, Ar*H*), 7.25 - 7.32 (m, 1 H, Ar*H*), 7.56 - 7.63 (m, 1 H, Ar*H*). **^{13}C-NMR** (101 MHz, $CDCl_3$) δ 9.6, 49.1, 55.1, 109.4, 110.6, 114.0, 118.7, 118.9, 121.5, 125.6, 128.1, 128.9, 129.8, 136.5, 158.9. **ATR-IR** ν_{max} (neat)/cm^{-1} 3042, 2912, 2832, 1612, 1585, 1512, 1465, 1244, 1173, 1032, 738. **HRMS (CI):** calcd for $C_{17}H_{18}NO^+$ ($[M+H]^+$): 252.1383, found: 252.1384.

3-Methyl-1-(4-methylbenzyl)-1*H*-indole (19)

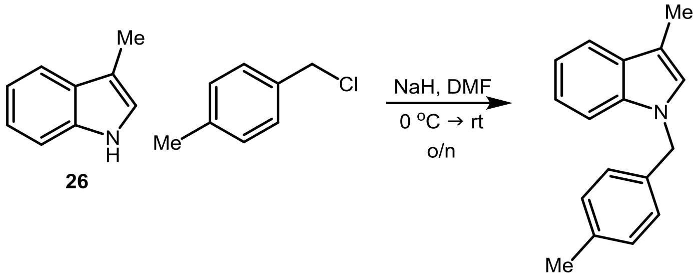

26

19, 28 %

Prepared according to the **General Procedure A - Benzylation of Indoles** from 3-methylindole **26** (655 mg, 5.00 mmol, 1 equiv.) and 4-methylbenzyl chloride (0.99 mL, 7.5 mmol, 1.5 equiv.). Purification by column chromatography (19:1 hexane:diethyl ether) afforded 3-methyl-1-(4-methylbenzyl)-1*H*-indole **19** as a colourless oil (335 mg, 28 %). **^{1}H-NMR** (400 MHz, $CDCl_3$) δ 2.33 (s, 3H, C*H*$_3$), 2.36 (d, *J* = 1.25 Hz, 3H, C*H*$_3$), 5.24 (s, 2H, C*H*$_2$), 6.90 (d, *J* = 0.8 Hz, 1H, Ar*H*), 7.04 (d, *J* = 8.0 Hz, 2H, Ar*H*), 7.09 - 7.16 (m, 3H, Ar*H*), 7.19 (td, *J* = 7.0, 2.0 Hz, 1H, Ar*H*), 7.28 (d, *J*=7.3 Hz, 1H, Ar*H*), 7.57 - 7.64 (m, 1H, Ar*H*). **^{13}C-NMR** (101 MHz, $CDCl_3$) δ 9.6, 21.0, 49.5, 109.4, 110.7, 118.6, 119.0, 121.5, 125.7, 126.8, 128.9, 129.3, 134.8, 136.6, 137.1. **ATR-IR** ν_{max} (neat)/cm^{-1} 3022, 2914, 1614, 1515, 1467, 1329, 1183, 1015, 736. **HRMS (CI):** calcd for $C_{17}H_{18}N^+$ ($[M+H]^+$): 236.1434, found: 236.1434.

3-Methyl-1-(1-phenylethyl)-1*H*-indole (20)

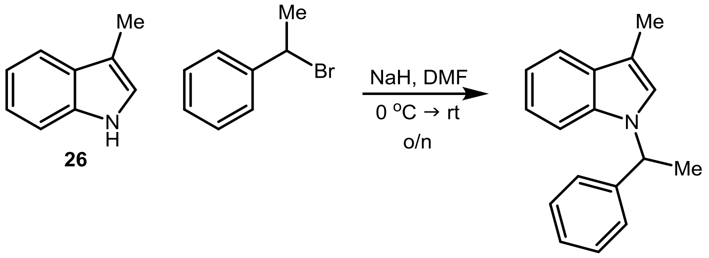

26

20, 60 %

Prepared according to the **General Procedure A - Benzylation of Indoles** from 3-methylindole **26** (655 mg, 5.00 mmol, 1.0 equiv.) and (1-bromoethyl)benzene (1.02 mL, 7.50 mmol, 1.5 equiv.). Purification by column chromatography (9:1 hexane:diethyl ether) afforded 3-methyl-1-(1-phenylethyl)-1*H*-indole **20** as a colourless oil (706 mg, 60%). **^{1}H-NMR** (400 MHz, $CDCl_3$) δ 1.90 (d, *J* = 5.9 Hz, 3H, C*H*$_3$), 2.36 (s, 3H, C*H*$_3$), 5.59 - 5.69 (q, J = 7.1 Hz, 1H, C*H*), 7.05 (s, 1H, Ar*H*), 7.07 - 7.18 (m, 4H, Ar*H*), 7.18 - 7.26 (m, 2H, Ar*H*), 7.27 - 7.33 (m, 2H, Ar*H*), 7.58 (d, *J* = 6.8 Hz, 1H, Ar*H*). **^{13}C-NMR** (101 MHz, $CDCl_3$) δ 9.8, 21.6, 54.4, 109.8, 110.5, 118.8, 118.9, 121.4, 122.4, 125.9, 127.3, 128.6, 128.9, 136.4, 142.9. **ATR-IR** ν_{max} (neat)/cm^{-1} 3024, 2972, 2912, 1612, 1452, 1355, 1233, 1186, 1015, 736, 699. **HRMS (CI):** calcd for $C_{17}H_{18}N^+$ ($[M+H]^+$): 236.1434, found: 236.1435.

3-Phenyl-1*H*-indole (27)[13]

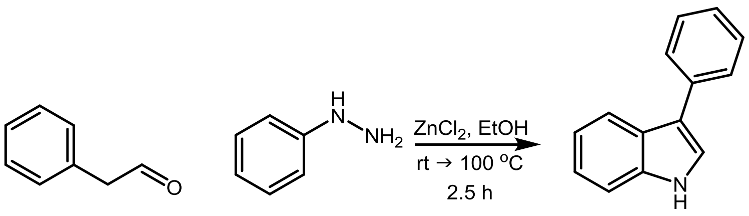

27, 69 %

A mixture of phenylacetaldehyde (1.17 mL, 10.0 mmol, 1.0 equiv.) and phenylhydrazine (0.98 mL, 10 mmol, 1.0 equiv.) was stirred at room temperature for 1 hour under argon. The mixture was then heated to 100 °C and stirred for 30 min. A solution of zinc chloride (2.45 g, 18.0 mmol, 1.8 equiv.) in ethanol (11 mL) was added to the reaction mixture and this was refluxed at 100 °C for 1 h. After cooling to room temperature, the mixture was filtered and the solvent was removed under reduced pressure. Hydrochloric acid (2 M) l was added to the crude residue and the organic products were extracted into DCM. The organic layers were dried over Na_2SO_4, filtered and concentrated under reduced pressure. Purification by column chromatography (3:1 hexane:diethyl ether) afforded 3-phenyl-1*H*-indole **27** as orange crystals (1.34 g, 69%). **Mp** = 81-83 °C (lit. mp =83-85 °C).[14] **^{1}H-NMR** (400 MHz, $CDCl_3$) δ 7.15 - 7.38 (m, 4H, Ar*H*), 7.41 (d, *J* = 7.9 Hz, 1H, Ar*H*), 7.50 (t, *J* = 7.6 Hz, 2H, 2 x Ar*H*), 7.72 (d, *J* = 7.5 Hz, 2H, Ar*H*), 8.01 (d, *J* = 7.9 Hz, 1H, Ar*H*), 8.08 (br. s., 1H, N*H*). **^{13}C-NMR** (101 MHz, $CDCl_3$) δ 111.4, 118.2, 119.8, 120.3, 121.8, 122.4, 125.7, 126.0, 127.4, 128.7, 135.5, 136.6. **ATR-IR** ν_{max} (neat)/cm^{-1} 3400, 1597, 1541, 1456, 1417, 1338, 1259, 1236, 1112, 1101, 1010, 823, 769, 694, 632. ***m/z* (EI)**: 193.2 (100, $[M]^+$), 165.1 (37)

1-Benzyl-3-phenyl-1*H*-indole (21)[15]

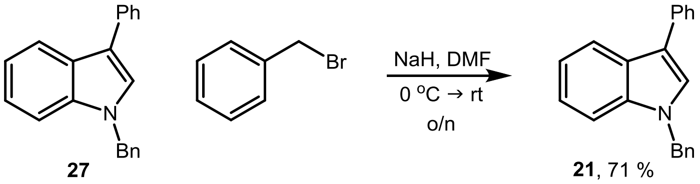

27 **21**, 71 %

Prepared according to the **General Procedure A - Benzylation of Indoles** from 3-phenylindole **27** (750 mg, 5.00 mmol, 1.0 equiv.) and benzyl bromide (0.89 mL, 7.5 mmol, 1.5 equiv.). Purified by column chromatography (1:19 diethyl ether:hexane) to afford **21** as a white solid (782 mg, 71%). **Mp** = 58-60 °C (lit. mp = 62-63 °C).[16] **^{1}H-NMR** (400 MHz, $CDCl_3$) δ 5.38 (s, 2H, CH_2), 7.17 - 7.26 (m, 4H, Ar*H*), 7.27 - 7.30 (m, 2H, Ar*H*), 7.30 - 7.38 (m, 4H, Ar*H*), 7.44 (t, *J* = 7.7 Hz, 2 H, Ar*H*), 7.68 (d, *J* = 7.0 Hz, 2 H, Ar*H*), 7.98 (d, *J* = 7.7 Hz, 1 H, Ar*H*). **^{13}C-NMR** (101 MHz, $CDCl_3$) δ 49.9, 110.0, 117.2, 120.0, 120.1, 122.1, 125.8, 125.9, 126.4, 126.8, 127.3, 127.6, 128.7, 128.7, 135.5, 137.1, 137.1. **ATR-IR** ν_{max} (neat)/cm^{-1} 3026, 1598, 1539, 1467, 1454, 1435, 1388, 1359, 1322, 1242, 1203, 1188, 1070, 1018, 968, 937, 898, 813, 763, 692. ***m/z* (EI):** 283.2 (100, $[M]^+$), 192.1 (51), 165.1 (24), 91.1 (86), 65.1 (14).

9-Benzyl-9*H*-carbazole (22)[17]

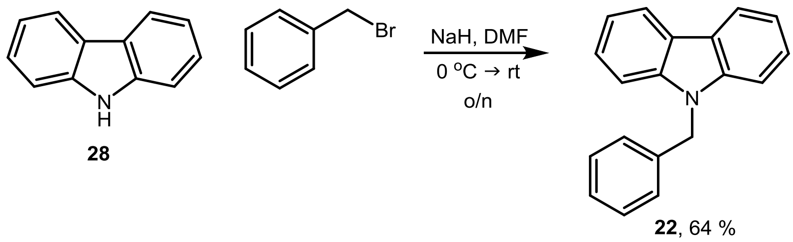

Prepared according to the **General Procedure A - Benzylation of Indoles** from 9*H*-carbazole **28** (836 mg, 5.00 mmol, 1.0 equiv.) and benzyl bromide (0.89 mL, 7.5 mmol, 1.5 equiv.). Purification by recrystallisation from diethyl ether afforded 9-benzyl-9*H*-carbazole **22** as a white solid (732 mg, 57%). **Mp** = 115-116 °C (lit. mp = 114-116 °C).[17] **^{1}H-NMR** (400 MHz, $CDCl_3$) δ 5.54 (s, 2H, C*H*$_2$), 7.16 (d, *J* = 7.0 Hz, 2H, Ar*H*), 7.21 - 7.33 (m, 5H, Ar*H*), 7.39 (d, *J* = 8.1 Hz, 2H, Ar*H*), 7.45 (td, *J* = 7.5, 0.8 Hz, 2H, Ar*H*), 8.15 (d, *J* = 7.8 Hz, 2H, Ar*H*). **^{13}C-NMR** (101 MHz, $CDCl_3$) δ 46.6, 108.9, 119.2, 120.4, 123.0, 125.8, 126.4, 127.4, 128.8, 137.2, 140.7. **ATR-IR** ν_{max} (neat)/cm^{-1} 3046, 2925, 1595, 1484, 1450, 1326, 1207, 995, 842, 747, 721, 695. ***m/z* (EI):** 257.1 (98, [M]$^+$), 207.0 (15), 180.1 (17), 166.1 (25), 140.1 (17), 91.0 (100), 65.0 (15)

3-Methyl-1-(naphthalen-1-ylmethyl)-1*H*-indole (23)

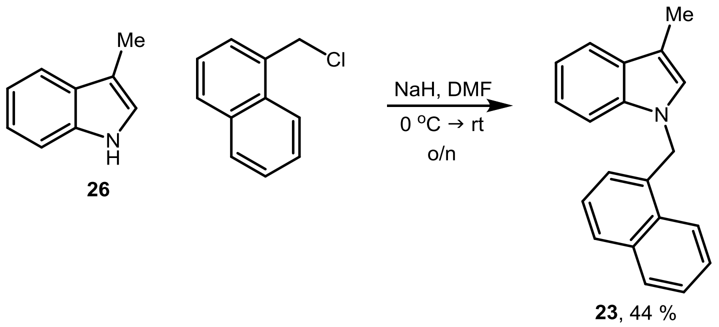

Prepared according to the **General Procedure A - Benzylation of Indoles** from 3-methylindole **26** (655 mg, 5.00 mmol, 1.0 equiv.) and 1-(chloromethyl)naphthalene (1.12 mL, 7.50 mmol, 1.5 equiv.). Purification by column chromatography (19:1 hexane:diethyl ether) afforded 3-methyl-1-(naphthalen-1-ylmethyl)-1*H*-indole **23** as a colourless oil (603 mg, 44%). **^{1}H-NMR** (400 MHz, $CDCl_3$) δ 2.37 (d, *J* = 1.0 Hz, 3H, C*H*$_3$), 5.73 (s, 2H, C*H*$_2$), 6.85 (d, *J* = 0.8 Hz, 1H, Ar*H*), 6.95 (dd, *J* = 7.2, 0.9 Hz, 1H, Ar*H*), 7.19 (td, *J* = 7.3, 1.4 Hz, 1H, Ar*H*), 7.24 (td, *J* = 7.5, 1.4 Hz, 1H, Ar*H*), 7.31 - 7.40 (m, 2 H, Ar*H*), 7.53 - 7.60, (m, 2H, Ar*H*), 7.65 - 7.70 (m, 1H, Ar*H*), 7.83 (d, *J* = 8.3 Hz, 1H, Ar*H*), 7.91 - 7.98 (m, 1H, Ar*H*), 7.98 - 8.05 (m, 1H, Ar*H*). **^{13}C-NMR** (101 MHz, $CDCl_3$) δ 9.7, 47.2, 109.3, 110.8, 118.9, 119.1, 121.6, 122.6, 124.8, 125.5, 125.7, 125.8, 126.4, 128.1, 128.8, 128.9, 130.8, 132.8, 133.6, 136.7. **ATR-IR** ν_{max} (neat)/cm^{-1} 3044, 2912, 1599, 1467, 1324, 1188, 1013, 792, 732. **HRMS (CI):** calcd for $C_{20}H_{19}N^+$ ([M+H]$^+$): 272.1434, found: 272.1436.

N-Benzyl-*N*-methylaniline (40)[18]

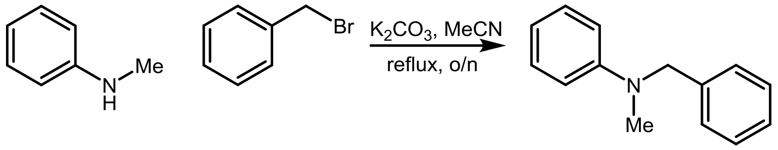

41 **40**, 61 %

N-Methylaniline **41** (0.54 mL, 5 mmol, 1 equiv.), potassium carbonate (1.38 g, 10.0 mmol, 2.0 equiv.) and benzyl bromide (0.71 mL, 6.0 mmol, 1.2 equiv.) were dissolved in acetonitrile (5.0 mL) and refluxed overnight. The reaction mixture was then cooled to room temperature and extracted into ethyl acetate, washed with water, dried over Na_2SO_4, filtered and concentrated under reduced pressure. Purification by column chromatography (9:1 petroleum ether:diethyl ether) afforded *N*-benzyl-*N*-methylaniline **40** as a brown oil (606 mg, 61%). **^{1}H-NMR** (400 MHz, $CDCl_3$) δ 3.04 (s, 3H, C*H*$_3$), 4.56 (s, 2H, C*H*$_2$), 6.68 - 6.82 (m, 3H, Ar*H*), 7.19 - 7.30 (m, 5H, Ar*H*), 7.30 - 7.38 (m, 2H, Ar*H*). **^{13}C-NMR** (101 MHz, $CDCl_3$) δ 38.5, 56.6, 112.4, 116.5, 126.7, 126.8, 128.5, 129.2, 139.0, 149.7. **ATR-IR** $ν_{max}$ (neat)/cm^{-1} 3059, 3024, 2877, 2812, 1597, 1502, 1450, 1352, 1247, 1211, 1190, 1114, 1026, 985, 943, 860, 746, 725. ***m/z* (EI):** 197.2 (94, [M]$^+$), 120.1 (78), 104.1 (12), 91.1 (100), 77.1 (30), 65.1 (20)

1-Allyl-3-methyl-1*H*-indole (43)[19]

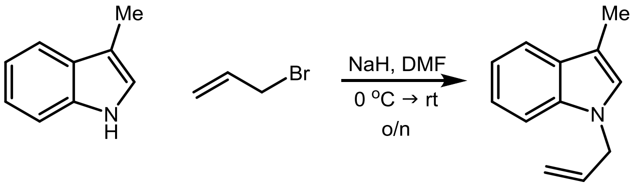

26 **43**, 64 %

Prepared according to the **General Procedure B - Allylation of Indoles** from 3-methylindole **26** (655 mg, 5.00 mmol, 1.0 equiv.) and allyl bromide (0.65 mL, 7.5 mmol, 1.5 equiv.). Purification by column chromatography (19:1 hexane:diethyl ether) afforded 1-allyl-3-methyl-1*H*-indole **43** as a pink oil (546 mg, 64%). **^{1}H-NMR** (400 MHz, $CDCl_3$) δ 2.38 (d, *J* = 1.0 Hz, 3H, C*H*$_3$), 4.70 (dt, *J* = 5.5, 1.5 Hz, 2H, C*H*$_2$), 5.13 (dq, *J* = 17.1, 1.6 Hz, 1H, C*H*), 5.22 (dq, *J* = 10.3, 1.4 Hz, 1H, C*H*), 6.02 (ddt, *J* = 17.1, 10.4, 5.3, 5.3 Hz, 1H, C*H*), 6.87 - 6.92 (m, 1 H, Ar*H*), 7.10 - 7.20 (m, 1H, Ar*H*), 7.24 (td, *J* = 7.5, 1.3 Hz, 1H, Ar*H*), 7.32 (d, *J* = 8.5 Hz, 1H, Ar*H*), 7.62 (dt, *J* = 7.8, 1.0 Hz, 1H, Ar*H*). **^{13}C-NMR** (101 MHz, $CDCl_3$) δ 9.6, 48.5, 109.3, 110.5, 117.0, 118.6, 119.0, 121.4, 125.4, 128.8, 133.8, 136.4. **ATR-IR** $ν_{max}$ (neat)/cm^{-1} 3053, 2914, 1463, 1436, 1417, 1384, 1361, 1328, 1186, 1126, 1012, 987, 918, 786, 688. ***m/z* (EI):** 171.2 (100, [M]$^+$), 156.1 (49), 144.1 (35), 130.1 (57), 115.1 (11), 103.1 (23), 89.1 (4), 77.1 (30), 63.1 (6)

1-Allyl-2,3-dimethyl-1*H*-indole (45)

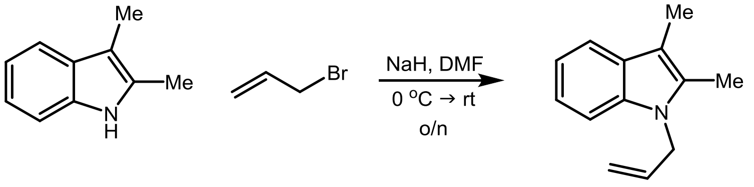

Prepared according to the **General Procedure B - Allylation of Indoles** from 2,3-dimethylindole **46** (726 mg, 5.00 mmol, 1.0 equiv.) and allyl bromide (0.65 mL, 7.5 mmol, 1.5 equiv.). Purification by column chromatography (19:1 hexane:diethyl ether) afforded 1-allyl-2,3-dimethyl-1*H*-indole **45** as a red oil (342 mg, 35%). **^{1}H-NMR** (400 MHz, $CDCl_3$) δ 2.27 (d, *J* = 0.5 Hz, 3H, CH_3), 2.33 (s, 3H, CH_3), 4.68 (dt, *J* = 4.6, 2.0 Hz, 2H, CH_2), 4.84 (dq, *J* = 17.2, 1.6 Hz, 1H, C*H*), 5.10 (dq, *J* = 10.3, 1.6 Hz, 1H, C*H*), 5.93 (ddt, *J* = 17.1, 10.3, 4.7, 4.7 Hz, 1H, C*H*), 7.03 - 7.17 (m, 2H, Ar*H*), 7.19 - 7.25 (m, 1H, Ar*H*), 7.46 - 7.55 (m, 1H, Ar*H*). **^{13}C-NMR** (101 MHz, $CDCl_3$) δ 8.8, 9.9, 45.2, 106.6, 108.6, 115.9, 117.9, 118.6, 120.5, 128.6, 132.2, 133.7, 136.0. **ATR-IR** ν_{max} (neat)/cm^{-1} 3053, 2980, 2912, 2856, 1643, 1614, 1570, 1487, 1413, 1363, 1328, 1261, 1215, 1182, 1147, 1126, 1016, 985, 916, 792, 663. **HRMS (CI):** calcd for $C_{13}H_{16}N^+$ ($[M+H]^+$): 186.1277, found: 186.1276.

(2-Methoxyoctan-2-yl)benzene (47)[20]

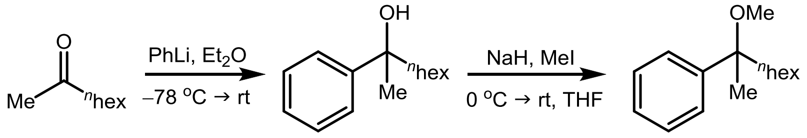

To a solution of 2-octanone (0.78 mL, 5.0 mmol, 1.0 equiv.) in diethyl ether (5.0 mL) under argon was added phenyllithium (3.3 mL of a 1.8 M solution in dibutyl ether, 6.0 mmol, 1.1 equiv.) dropwise at -78 °C. The solution was then warmed to room temperature and stirred for 1 h. The reaction was then quenched by careful addition of isopropanol, followed by hydrochloric acid (1 M). The organic materials were then extracted into diethyl ether, washed with brine, dried over Na_2SO_4, filtered and concentrated. Purification by column chromatography (19:1 hexane:diethyl ether) afforded 2-phenyloctan-2-ol as a colourless oil (610 mg, 59%). **^{1}H-NMR** (400 MHz, $CDCl_3$) δ 0.79 - 0.91 (m, 3H, CH_3), 1.12 - 1.35 (m, 8H, 4 x CH_2), 1.56 (s, 3H, CH_3), 1.73 - 1.87 (m, 2H, CH_2), 7.25 (tt, *J* = 7.3, 1.5 Hz, 1H, Ar*H*), 7.35 (tt, *J* = 7.5, 2.3 Hz, 2H, Ar*H*), 7.39 - 7.50 (m, 2H, Ar*H*). **^{13}C-NMR** (101 MHz, $CDCl_3$) δ 14.0, 22.6, 23.9, 29.6, 30.1, 31.7, 44.2, 74.7, 124.8, 126.4, 128.1, 148.1. **ATR-IR** ν_{max} (neat)/cm^{-1} 3414, 2929, 1492, 1444, 1373, 1151, 1067, 1028, 931, 900, 862, 763. ***m/z* (EI):** 206.2 (1, $[M]^+$), 191.2 (13), 121.1 (100), 105.1 (34), 91.1 (21), 77.1 (35).

2-Phenyloctan-2-ol (610 mg, 2.96 mmol, 1.0 equiv.) was added at 0 °C to a suspension of sodium hydride (71 mg, 3.0 mmol, 1.0 equiv.) in THF (5.0 mL) under nitrogen. The resulting solution was then stirred for 45 min at room temperature before again cooling to 0 °C. Methyl iodide (0.28 mL, 4.4 mmol, 1.5 equiv.) was added dropwise before being warmed to room temperature and stirred overnight. The reaction mixture was quenched with water and extracted into diethyl ether. The organic phase was

washed with water and brine, then dried over Na_2SO_4, filtered and concentrated. Purification by column chromatography (2:98 diethyl ether:hexane) afforded (2-methoxyoctan-2-yl)benzene **47** as a colourless oil (271 mg, 42%). **^{1}H-NMR** (400 MHz, $CDCl_3$) δ 0.85 (t, *J* = 6.9 Hz, 3H, C*H*$_3$), 1.08 - 1.18 (m, 1H, C*H*), 1.18 - 1.31 (m, 7H, 7 x C*H*), 1.53 (s, 3H, C*H*$_3$), 1.69 - 1.80 (m, 2H, C*H*$_2$), 3.09 (s, 3H, C*H*$_3$), 7.22 - 7.28 (m, 1H, Ar*H*), 7.32 - 7.42 (m, 4H, Ar*H*). **^{13}C-NMR** (101 MHz, $CDCl_3$) δ 14.0, 22.6, 23.0, 23.9, 29.7, 31.8, 42.7, 50.3, 79.1, 126.2, 126.6, 128.0, 145.4. **ATR-IR** ν_{max} (neat)/cm^{-1} 2929, 2854, 1492, 1480, 1444, 1165, 1132, 1072, 1023, 912, 840, 763. ***m/z* (EI):** 205.2 (4, [M-Me]$^+$), 135.1 (100), 105.1, (22), 91.1 (19), 77.1 (18).

2-Phenyl-2-propylpentanenitrile (49)

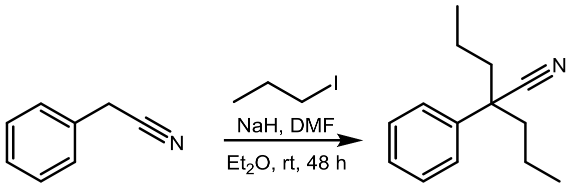

49, 79 %

To a suspension of NaH (227 mg, 9.45 mmol, 3.15 equiv.) in DMF (11 mL) was added benzyl cyanide (0.35 mL, 3 mmol, 1 equiv.) and 1-iodopropane (1.32 mL, 13.5 mmol, 4.5 equiv.) in diethyl ether (5.5 mL) at room temperature. This mixture was stirred for 48 h at room temperature, and was then quenched with methanol and filtered. After washing with ether, the filtrates were concentrated under reduced pressure, re-dissolved in ether and then washed with water, sodium bisulfite solution and sodium carbonate solution. The organic layer was then dried over Na_2SO_4, filtered and concentrated. Purification by column chromatography (49:1 hexane:diethyl ether) afforded 2-phenyl-2-propylpentanenitrile **49** as a colourless oil (476 mg, 79 %). **^{1}H-NMR** (400 MHz, $CDCl_3$) 0.89 (t, *J* = 7.5 Hz, 6 H), 1.05 - 1.24 (m, 2 H), 1.40 - 1.58 (m, 2 H), 1.79 - 2.03 (m, 4 H), 7.25 - 7.34 (m, 1 H), 7.35 - 7.44 (m, 4 H). **^{13}C-NMR** (101 MHz, $CDCl_3$) 13.9, 18.6, 43.2, 48.3, 122.7, 125.9, 127.5, 128.8, 138.8 **ATR-IR** ν_{max} (neat)/cm^{-1} 2958, 2872, 2233, 1600, 1492, 1463, 1448, 1379, 1201, 1112, 1083, 1029, 912, 763, 738, 698. ***m/z* (EI):** 201.2 (25, M$^+$), 159.1 (77), 130.1 (78), 116.1 (100), 103.1 (48), 91.1 (14), 77.1 (16). **HRMS (CI):** calcd for $C_{14}H_{20}N^+$ ([M+H]$^+$): 202.1596, found: 202.1594

1,2-Diphenylethyne (54)[21]

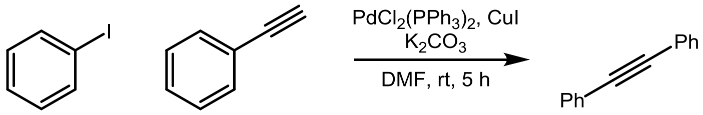

54, 60 %

The reaction vessel was charged with bis(triphenylphosphine)palladium(II) dichloride (175 mg, 0.250 mmol, 0.05 equiv.), copper(I) iodide (95 mg, 0.50 mmol, 0.1 equiv.), potassium carbonate (2.07 g, 15.0 mmol, 3.0 equiv.) and DMF (17 mL). Then iodobenzene (0.56 mL, 5.0 mmol, 1.0 equiv.) and ethynylbenzene (0.55 mL, 5.0 mmol, 1.0 equiv.) were added and the reaction was stirred at room temperature for 5 h. The reaction was partitioned between water (80 mL) and diethyl ether (20 mL).

The aqueous phase was extracted with diethyl ether (3 x 20 mL). The combined organic phases were washed with water (50 mL) and brine. They were dried over $MgSO_4$ and concentrated under reduced pressure in the presence of silica. Purification by flash column chromatography (hexane) gave 1,2-diphenylethyne **54** as a white solid (534 mg, 60%). **mp** = 57 -58 °C (lit mp = 58-60 °C)[22]. **^{1}H-NMR** (400 MHz, $CDCl_3$) δ 7.31 - 7.42 (m, 6H), 7.52 - 7.60 (m, 4H). **^{13}C-NMR** (101 MHz, $CDCl_3$) δ 89.5, 123.4, 128.4, 128.5, 131.7. **GC-MS** [*m/z* (%)]: 178 (M^+, 100), 177 (10), 176 (20), 152 (15), 151 (10), 150 (10), 139 (5), 126 (10), 98 (5), 89 (5), 88 (5), 76 (10), 75 (5), 63 (5), 51 (5), 50 (5).

Literature synthesis of 1,4-Dihydronaphthalene (58)[23]

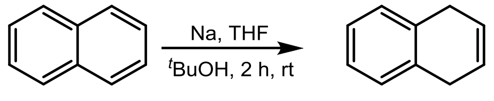

53 **58**, 75%

To a solution of naphthalene (1.28 g, 10.0 mmol, 1.0 equiv.) in THF (21 mL) was added sodium (0.575 g, 25.0 mmol, 2.5 equiv.) in small portions over ca 2 min. 10 min after the addition was finished, *tert*-butanol (2.39 mL, 25.0 mmol, 2.5 equiv.) was added slowly and the reaction was stirred at room temperature for 2 h. The remaining sodium was removed from the reaction. The crude was partitioned between water (30 mL) and diethyl ether (30 mL). The phases were separated and the aqueous phase was extracted with diethyl ether (3 x 20 mL). The combined organic phases were washed with brine, dried over $MgSO_4$ and concentrated under reduced pressure to give the crude product as a pale-yellow oil. Distillation (70 °C ±1 °C at 8.5 mm Hg) gave the product 1,4-dihydronaphthalene **58** as a colourless oil (1.08 g, 75%). **^{1}H-NMR** (400 MHz, $CDCl_3$) δ 3.41 (d, *J* = 1.4 Hz, 4H), 5.94 (m, 2H), 7.11 - 7.19 (m, 4H). **^{13}C-NMR** (101 MHz, $CDCl_3$) δ 29.9, 124.9, 126.0, 128.6, 134.4. **GC-MS** [m/z (%)]: 130 (M^+, 100), 129 (95), 128 (60), 127 (25), 126 (10), 115 (65), 103 (5), 102 (20), 89 (10), 87 (5), 78 (10), 77 (15), 76 (10), 75 (15), 74 (15), 64 (10), 63 (30), 62 (15), 52 (10), 51 (35), 50 (15).

Products

1*H*-Indole (24)[24]

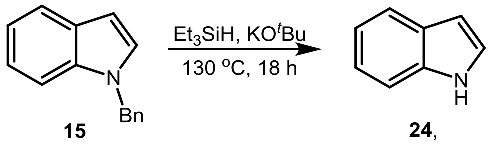

15 **24**, 29%

Carried out according to General Procedure C - Debenzylation/Deallylation of Indole Derivatives from **15** and purified by column chromatography (9:1 hexane:diethyl ether) followed by recrystallisation from hexane to afford **24** as a yellow solid (17 mg, 29 %). **mp** = 50-51 °C (lit. mp = 52-54 °C).[25] **^{1}H-NMR** (400 MHz, $CDCl_3$) δ 6.58 (ddd, *J* = 3.2, 2.1, 1.0 Hz, 1H, Ar*H*), 7.05 - 7.17 (m, 1H, Ar*H*), 7.18 - 7.24 (m, 2H, Ar*H*), 7.37 - 7.46 (m, 1H, Ar*H*), 7.62 - 7.71 (m, 1H, Ar*H*), 8.15 (br. s., 1H, N*H*). **^{13}C-NMR**

(101 MHz, $CDCl_3$) δ 102.7, 111.0, 119.8, 120.7, 122.0, 124.1, 127.9, 135.8. **ATR-IR** ν_{max} (neat)/cm^{-1} 3393, 3047, 1576, 1455, 1335, 1247, 1059, 743, 721. ***m/z*(EI):** 117.0 (100, $[M]^+$), 90.0 (42), 63.0 (24)

2-Methyl-1*H*-indole (25)[26] and 2-(2-methylbenzyl)-1*H*-indole (29)[27]

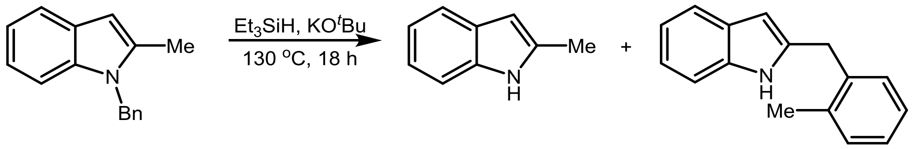

16 **25**, 49 % **29**, 15%

Carried out according to General Procedure C - Debenzylation/Deallylation of Indole Derivatives from **16.** Purification by column chromatography (9:1 hexane:diethyl ether) afforded 2-methyl-1*H*-indole **25** as an orange solid (32 mg, 49 %). **mp** = 53-54 °C (lit. mp = 56-57 °C).[26] **^{1}H-NMR** (400 MHz, $CDCl_3$) δ 2.46 (s, 3H, C*H*$_3$), 6.17 - 6.30 (m, 1H, Ar*H*), 7.08 (td, *J* = 7.3, 1.1 Hz, 1H, Ar*H*), 7.12 (td, *J* = 6.9, 1.4 Hz, 1 H, Ar*H*), 7.29 (d, *J* = 7.8 Hz, 1 H, Ar*H*), 7.53 (d, *J* = 7.6 Hz, 1 H, Ar*H*), 7.84 (br. s., 1 H, N*H*). **^{13}C-NMR** (101 MHz, $CDCl_3$) δ 13.7, 100.4, 110.2, 119.6, 119.6, 120.9, 129.1, 135.0, 136.0. **ATR-IR** ν_{max} (neat)/cm^{-1} 3379, 3050, 2936, 1549, 1452, 1344, 1285, 1037, 783, 749. ***m/z* (EI):** 131.1 (75, $[M]^+$), 130.1 (100), 103.1 (11), 89.0 (4), 77.0 (12), 63.0 (6).

Column chromatography (9:1 hexane:diethyl ether) also afforded 2-(2-methylbenzyl)-1*H*-indole **29** as a brown oil (16 mg, 15 %). **^{1}H-NMR** (400 MHz, $CDCl_3$) δ 2.31 (s, 3H, C*H*$_3$), 4.14 (s, 2H, C*H*$_2$), 6.22 - 6.33 (m, 1H, Ar*H*), 7.03 - 7.17 (m, 2H, Ar*H*), 7.18 - 7.24 (m, 4H, Ar*H*), 7.24 - 7.27 (m, 1 H, Ar*H*), 7.51 - 7.57 (m, 1 H, Ar*H*), 7.73 (br. s., 1 H, N*H*). **^{13}C-NMR** (101 MHz, $CDCl_3$) δ 19.4, 32.6, 100.9, 110.4, 119.7, 119.9, 121.2, 126.3, 127.1, 128.8, 129.5, 130.6, 136.0, 136.5, 136.9, 137.2. **ATR-IR** ν_{max} (neat)/cm^{-1} 3412, 3050, 2912, 1603, 1495, 1452, 1328, 1181, 1013, 747, 727. ***m/z* (EI):** 221.1 (100, $[M]^+$), 204.1 (20), 130.1 (52), 117.1 (28), 104.1 (30), 89.0 (7), 77.0 (11), 63.0 (5)

3-Methyl-1*H*-indole (26)[28]

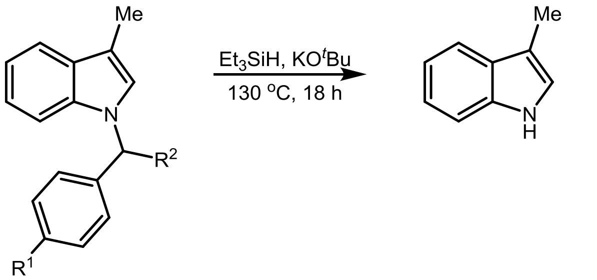

17. R^1=H, R^2=H **26**. 73 %
18. R^1=OMe, R^2=H **26**. 76 %
19. R^1=Me, R^2=H **26**. 63 %
20. R^1=H, R^2=Me **26**. 47 %

Carried out according to General Procedure C - Debenzylation/Deallylation of Indole Derivatives from **17-20.** Purification by column chromatography (9:1 hexane:diethyl ether) afforded 3-methyl-1*H*-indole **26** as a yellow solid in the yields indicated above . **mp** = 91-93 °C (lit. mp = 97 °C).[28] **^{1}H-NMR** (400 MHz, $CDCl_3$) δ 2.35 (d, *J* = 1.3 Hz, 3H, CH_3), 6.96 - 7.00 (m, 1H, Ar*H*), 7.13 (ddd, *J* = 7.9, 6.9, 1.3 Hz, 1H, Ar*H*), 7.20 (td, *J* = 7.3, 1.5 Hz, 1H, Ar*H*), 7.36 (dt, *J* = 8.0, 0.9 Hz, 1H, Ar*H*), 7.56 - 7.63 (m, 1H, Ar*H*), 7.88 (br. s., 1H, N*H*). **^{13}C-NMR** (101 MHz, $CDCl_3$) δ 9.6, 110.9, 111.8, 118.8, 119.1, 121.5, 121.9, 128.3, 136.3. **ATR-IR** ν_{max} (neat)/cm^{-1} 3396, 3052, 2916, 1616, 1456, 1333, 1088, 1010, 738. ***m/z* (EI)** 131.1 (55, $[M]^+$), 130.0 (100), 103.0 (8), 77.0 (19), 63.0 (6), 51.0 (9).

9*H*-Carbazole (28)[29]

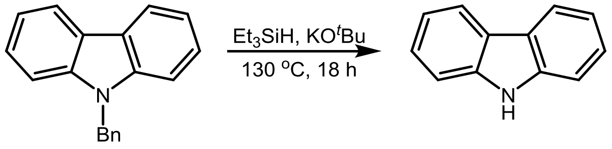

22 **28**, 57%

Carried out according to General Procedure C - Debenzylation/Deallylation of Indole Derivatives from **22**. Purification by column chromatography (9:1 hexane:diethyl ether) afforded 9*H*-carbazole **28** as a white solid (48 mg, 57 %). **mp** = 238-240 °C (lit. 237-238 °C).[30] **^{1}H-NMR** (400 MHz, $CDCl_3$) δ 7.22 - 7.27 (m, 2H, Ar*H*), 7.38 - 7.50 (m, 4H, Ar*H*), 8.06 (br. s., 1H, N*H*), 8.07 - 8.13 (m, 2H, Ar*H*). **^{13}C-NMR** (101 MHz, $CDCl_3$) δ 110.6, 119.4, 120.3, 123.4, 125.8, 139.5. **ATR-IR** ν_{max} (neat)/cm^{-1} 3412, 3050, 1599, 1450, 1326, 930, 747, 723. ***m/z* (EI):** 167.1 (100, $[M]^+$), 139.0 (15), 113.0 (4), 83.5 (8).

1-Methylnaphthalene (30)[31]

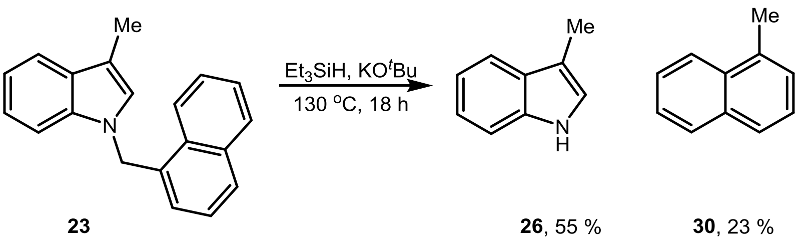

23 **26**, 55 % **30**, 23 %

Carried out according to General Procedure C - Debenzylation/Deallylation of Indole Derivatives from **23.** Purification by column chromatography (hexane until elution of first product, then 1:9 diethyl ether:hexane) afforded **26** as a yellow solid (36 mg, 55 %) and **30** as a mixture with *tert*-butoxy triethylsilane. The mixture was treated with concentrated hydrochloric acid (36%, w/v, 2 mL) in THF (2 mL) for 2 days at room temperature open to air. The initially colourless reaction turned wine-red. The crude reaction was diluted with water (10 mL) and extracted with dichloromethane (4 x 15 mL). The combined organic phases were dried over $MgSO_4$ and carefully concentrated under reduced pressure (45 °C, < 15 min, > 100 mbar). Purification by flash column chromatography (hexane) afforded 1-methylnaphthalene **30** as a colourless oil (16 mg, 23 %). **^{1}H-NMR** (400 MHz, $CDCl_3$) δ 2.72 (s, 3 H), 7.34 (d, *J* = 7.0 Hz, 1H), 7.36 - 7.41 (m, 1H), 7.48 - 7.52 (m, 1H), 7.54 (ddd, *J* = 8.5, 6.8, 1.6 Hz, 1H), 7.72 (d, *J* = 8.1 Hz, 1H), 7.86 (d, *J* = 7.8 Hz, 1H), 8.02 (d, *J* = 8.3 Hz, 1H). **^{13}C-NMR** (101 MHz, $CDCl_3$)

δ 19.5, 124.2, 125.7, 125.7, 125.8, 126.5, 126.7, 128.7, 132.7, 133.7, 134.4. [*m/z* (%)]: 142 (M^+, 100), 141 (75), 139 (10), 115 (40) 63 (5).

***N*-Methyl-*N*-phenylacetamide (42)**[32]

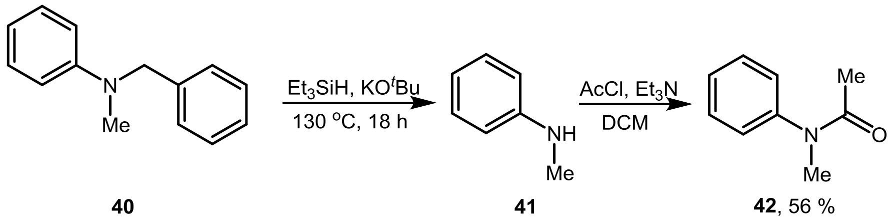

Aniline **40** (99 mg, 0.5 mmol, 1 equiv.), Et_3SiH (0.24 mL, 1.5 mmol, 3 equiv.), and KO^tBu (168 mg, 1.5 mmol, 3 equiv.) were sealed in a pressure tube in a glovebox under nitrogen. The tube was removed and heated at 130 °C for 18 h behind a shield. After cooling to room temperature, the reaction was quenched with 1 mL of water and 1,3,5-trimethoxybenzene (10 mol %, 8.4 mg) in $CDCl_3$ (3 mL) was added as an internal NMR standard. This indicated that the yield of *N*-methylaniline **41** was 65 %. This crude mixture was dissolved in DCM and triethylamine (0.08 mL, 0.5 mmol, 1.1 equiv.) added. This solution was cooled to 0 °C and acetyl chloride (0.04 mL, 0.5 mmol, 1 equiv.) added dropwise. After 10 min, the solution was warmed to room temperature and stirred at this temperature for 3 h before quenching with 1 M HCl and extracting into DCM. The resulting organic layers were dried over Na_2SO_4, filtered and concentrated under reduced pressure. Recrystallisation from hexane gave *N*-methyl-*N*-phenylacetamide **42** as off-white crystals (42 mg, 56 %). **mp** = 86-88 °C (lit. mp = 94- 95 °C).[32] **^{1}H-NMR** (400 MHz, $CDCl_3$) δ ($CDCl_3$ 1.88 (s, 3H, CH_3), 3.28 (s, 3H, CH_3), 7.15 - 7.24 (m, 2H, Ar*H*), 7.34 (t, *J* = 8.0 Hz, 1H, Ar*H*), 7.39 - 7.46 (m, 2H, Ar*H*). **^{13}C-NMR** (101 MHz, $CDCl_3$) δ 22.4, 37.1, 127.1, 127.7, 129.7, 144.6, 170.6. **ATR-IR** ν_{max} (neat)/cm^{-1} 3043, 1647, 1595, 1492, 1417, 1382, 1296, 1139, 1083, 1028, 970, 773, 623. ***m/z* (EI)** 149.1 (30, $[M]^+$), 106.1 (100), 77.0 (30), 65.0 (9), 51.0 (20).

2-Isopropylaniline (44)[33]

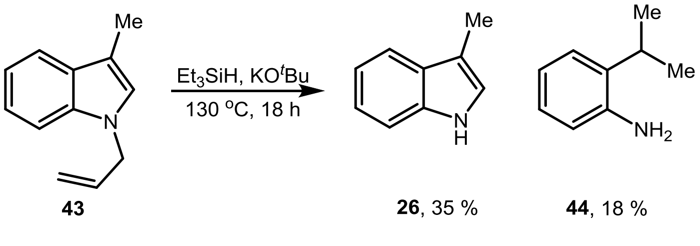

Carried out according to General Procedure C - Debenzylation/Deallylation of Indole Derivatives from **43**. Purification by column chromatography (9:1 hexane:diethyl ether) afforded **26** as a yellow solid (23 mg, 35 %) and 2-isopropylaniline **44** as a yellow oil (12 mg, 18 %). **^{1}H-NMR** (400 MHz, $CDCl_3$) δ 1.28 (d, *J* = 7.0 Hz, 6H), 2.85 - 2.99 (spt, *J* = 6.8 Hz, 1H), 3.66 (br. s., 2H), 6.69 (dd, *J* = 7.8, 1.3 Hz, 1H), 6.80 (td, *J* = 8.0, 1.3 Hz, 1H), 6.98 - 7.07 (m, 1H), 7.16 (dd, *J* = 7.5, 1.5 Hz, 1H). **^{13}C-NMR** (101 MHz, $CDCl_3$) δ 22.3, 27.6, 115.8, 119.0, 125.4, 126.5, 132.7, 143.3. **ATR-IR** ν_{max} (neat)/cm^{-1} 3456, 3369, 2956, 2927, 2870, 1618, 1494, 1454, 1381, 1361, 1292, 1261, 1155, 1033, 929, 721. ***m/z* (EI):** 135.1 (26, $[M]^+$), 120.1 (100), 103.1 (11), 93.1 (18), 77.1 (20), 65.0 (19), 51.0 (10).

2,3-Dimethyl-1*H*-indole (46)[34]

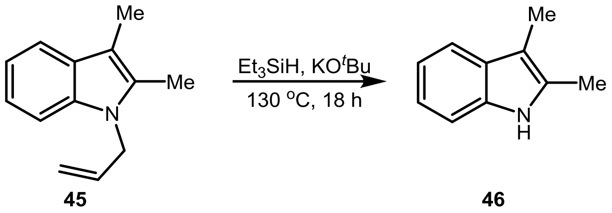

Carried out according to General Procedure C - Debenzylation/Deallylation of Indole Derivatives from **45.** Purification by column chromatography (4:1 hexane:diethyl ether) afforded **46** as a red solid. (24 mg, 33 %). **mp** = 98-101 °C (lit. mp = 104-106 °C).[35] **^{1}H-NMR** (400 MHz, $CDCl_3$) δ 2.24 (s, 3H, C*H*$_3$) 2.38 (s, 3H, C*H*$_3$) 7.05 - 7.14 (m, 2H, Ar*H*) 7.25 - 7.27 (m, 1H, Ar*H*) 7.48 (d, *J* = 7.2 Hz, 1H, Ar*H*) 7.68 (br. s., 1H, N*H*). **^{13}C-NMR** (101 MHz, $CDCl_3$) δ 8.4, 11.5, 107.1, 110.0, 117.9, 119.0, 120.9, 129.5, 130.6, 135.2. **ATR-IR** ν_{max} (neat)/cm^{-1} 3404, 2954, 2912, 2870, 2360, 1610, 1462, 1240, 1143, 1105, 1008, 669. ***m/z* (EI):** 145.1 (81, [M]$^+$), 144.1 (100), 130.1 (57), 115.1 (13), 102.1 (9), 89.1 (6), 77.1 (17), 63.0 (11), 51.0 (13).

Octan-2-ylbenzene (48)[36]

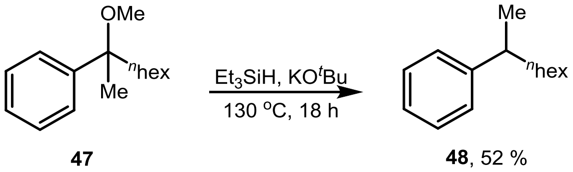

(2-Methoxyoctan-2-yl)benzene **47** (110 mg, 0.5 mmol, 1 equiv.), triethylsilane (0.24 mL, 1.5 mmol, 3 equiv.) and potassium *tert*-butoxide (168 mg, 1.5 mmol, 3 equiv.) were sealed in a pressure tube in a glovebox under nitrogen. The tube was removed and heated at 130 °C for 18 h behind a safety shield. After cooling to room temperature, the mixture was diluted with water and extracted into diethyl ether. The combined organic layers were dried over Na_2SO_4, filtered and concentrated under reduced pressure. Purification by column chromatography (hexane) afforded octan-2-ylbenzene **48** which co-eluted with some silyl byproducts. This mixture was dissolved in diethyl ether (3 mL) and hydrochloric acid (conc. 3 mL) added. This mixture was stirred overnight at room temperature before being diluted with water, extracted into DCM, dried over Na_2SO_4, filtered and concentrated under reduced pressure to afford an inseparable mixture of **48** and a small amount of a silyl impurity as a colourless oil (49 mg, 52 %). **^{1}H-NMR** (400 MHz, $CDCl_3$) δ 0.84 - 0.91 (m, 3H, C*H*$_3$), 1.16 - 1.33 (m, 11H, aliphatic C*H*), 1.55 - 1.64 (m, 2H, C*H*$_2$), 2.60 - 2.74 (m, 1H, C*H*), 7.13 - 7.23 (m, 3H, Ar*H*), 7.27 - 7.33 (m, 2H, Ar*H*). **^{13}C-NMR** (101 MHz, $CDCl_3$) δ 14.1, 22.3, 22.6, 27.7, 29.4, 31.8, 38.5, 39.9, 125.7, 127.0, 128.2, 148.0. **ATR-IR** ν_{max} (neat)/cm^{-1} 2954, 1922, 2852, 1492, 1452, 1375, 1014, 759, 719, 698. ***m/z* (EI):** 190.2 (16, [M]$^+$), 105.2 (100), 91.1 (17), 77.1 (9).

4-Phenylheptane (50)

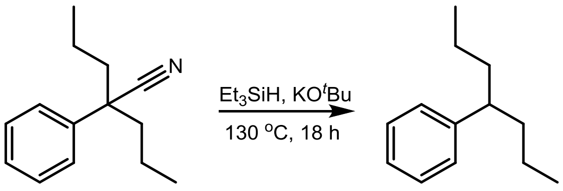

49 **50**, 43 %

Compound **49** (101 mg, 0.5 mmol, 1 equiv.), Et_3SiH (0.24 mL, 1.5 mmol, 3 equiv.) and KO^{t}Bu (168 mg, 1.5 mmol, 3 equiv.) were sealed in a pressure tube in a glovebox under nitrogen. The tube was removed and heated at 130 °C for 18 h behind a safety shield. After cooling to room temperature, the mixture was diluted with water and extracted into diethyl ether. The combined organic layers were dried over Na_2SO_4, filtered and concentrated under reduced pressure. Purification by column chromatography (hexane) afforded 4-phenylheptane **50** which was inseparable from some silyl-derived by-products. This crude mixture was then dissolved in diethyl ether (3 mL) and conc. HCl (3 mL) added. This mixture was stirred at room temperature for 48 h under air before diluting with water and extracting into hexane. The organic layers were washed with $NaHCO_3$, dried over Na_2SO_4, filtered and concentrated to afford 4-phenylheptane **50** as a colourless oil (38 mg, 43 %) **^{1}H-NMR** (400 MHz, $CDCl_3$) 0.86 (t, *J* = 7.3 Hz, 6 H), 1.11 - 1.25 (m, 4 H), 1.48 - 1.70 (m, 4 H), 2.47 - 2.59 (m, 1 H), 7.11 - 7.22 (m, 3 H), 7.26 - 7.33 (m, 2 H). **^{13}C-NMR** (101 MHz, $CDCl_3$) 14.1, 20.7, 39.2, 45.5, 125.7, 127.7, 128.1, 146.3. **ATR-IR** ν_{max} (neat)/cm^{-1} 2953, 2924, 2870, 1610, 1492, 1452, 1377, 1099, 1066, 1029, 1008, 759, 731, 665 ***m/z* (EI):** 176.2 (13, M$^+$), 133.1 (25), 91.1 (100). **HRMS (CI):** calcd for $C_{13}H_{20}^+$ ([M]$^+$): 176.1560, found: 176.1562

9,10-Dihydroanthracene (56)[37]

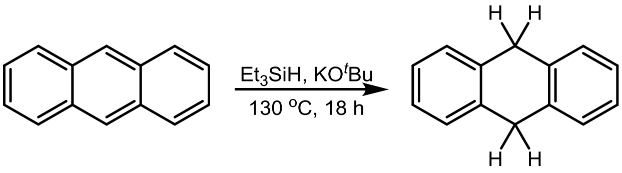

51 **56**, 85 %

The compound was synthesised according to the **General Procedure F – Reduction of Arenes** from anthracene **51**. Anthracene **51** (89 mg, 0.5 mmol, 1 equiv.), Et_3SiH (2.4 mL, 15 mmol, 30 equiv.) and KO^{t}Bu (1.68 g, 15 mmol, 30 equiv.) were sealed in a pressure tube in a glovebox under nitrogen. The tube was removed and heated at 130 °C for 18 h behind a safety shield. Purification by flash column chromatography (hexane) gave 9,10-dihydroanthracene **56** as a white solid (77.9 mg, 85 %). **mp** = 116-118 °C (lit. mp = 108-109 °C)[38]. **^{1}H-NMR** (400 MHz, $CDCl_3$) δ 3.96 (s, 4H, CH_2), 7.21 (dd, *J* = 5.6, 3.3 Hz, 4H, ArH), 7.31 (dd, *J* = 5.5, 3.4 Hz, 4H, ArH). **^{13}C-NMR** (101 MHz, $CDCl_3$) δ 36.3, 126.2, 127.5, 136.8. **GC-MS** [*m/z* (%)]: 180 (M$^+$, 100), 179 (95), 178 (55), 176 (10), 165 (25), 152 (10), 89 (10), 76 (5).

9,10-Dihydrophenanthrene (57)[39]

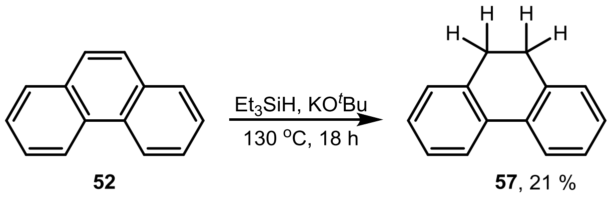

The compound was synthesised according to the **General Procedure F – Reduction of Arenes** from phenanthrene **52**. Phenanthrene **52** (89 mg, 0.5 mmol, 1 equiv.), Et_3SiH (2.4 mL, 15 mmol, 30 equiv.) and KO^{t}Bu (1.68 g, 15 mmol, 30 equiv.) were sealed in a pressure tube in a glovebox under nitrogen. The tube was removed and heated at 130 °C for 18 h behind a safety shield. Purification by flash column chromatography (hexane) gave 9,10-dihydrophenanthrene **57** as a colourless oil (24.6 mg, 27 %). **^{1}H-NMR** (400 MHz, $CDCl_3$) δ 2.89 (s, 4H), 7.21 - 7.28 (m, 4H), 7.32 (ddd, *J* = 7.7, 5.4, 3.5 Hz, 2H), 7.77 (dd, *J* = 7.6, 0.9 Hz, 2H). **^{13}C-NMR** (101 MHz, $CDCl_3$) δ 29.2, 123.8, 127.1, 127.5, 128.3, 134.6, 137.5. [*m/z* (%)]: 180 (M^+, 100), 179 (75), 178 (50), 176 (15), 165 (40), 152 (15), 151 (10), 89 (20), 88 (5), 76 (10).

1,2-Diphenylethane (59)[40]

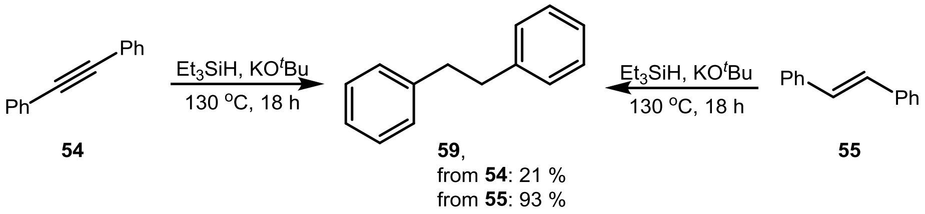

The compound **59** was synthesised according to the **General Procedure F – Reduction of Arenes** from 1,2-diphenylethyne **54** or from (E)-1,2-diphynelethene **55**. (E)-1,2-Diphenylethyne **54** (90 mg, 0.5 mmol, 1 equiv.) or 1,2-diphenylethene **55** (89 mg, 0.5 mmol, 1 equiv.), Et_3SiH (2.4 mL, 15 mmol, 30 equiv.) and KO^{t}Bu (1.68 g, 15 mmol, 30 equiv.) were sealed in a pressure tube in a glovebox under nitrogen. The tube was removed and heated at 130 °C for 18 h behind a safety shield. Purification by flash column chromatography (hexane) gave **59** as a white solid (from **54**: 24.5 mg, 23 %; from **55**: 83.4 mg, 93 %). **mp** = 46-47 °C (lit. mp = 50.5 -51 °C).[41] **^{1}H-NMR** (400 MHz, $CDCl_3$) δ 2.93 (s, 4H, CH_2), 7.17 - 7.23 (m, 6H, ArH), 7.26 - 7.32 (m, 4H, ArH). **^{13}C-NMR** (101 MHz, $CDCl_3$) δ 38.1, 126.1, 128.5, 128.6, 141.9. **GC-MS** [*m/z* (%)]: 182 (M^+, 30), 91 (100), 65 (25), 63 (5), 51 (5)

Suggested Mechanism for the Formation of 29

A suggested mechanism for the formation of **29** is shown below. The 2-methyl group of the indole must be activated, and KO^{t}Bu, or a more complex base formed in situ from Et_3SiH and KO^{t}Bu could achieve this, The reaction involved breaking the *N*-benzyl bond and forming a *C*-aryl bond to the *ortho* position of the benzyl unit. If the *N*-benzyl group were liberated during this reaction, it would be expected to use the benzylic carbon to form a new C-C bond. The fact that this has not happened and

that it forms a new bond through the *ortho* carbon suggests that an intramolecular migration has occurred that is regiospecific with respect to the benzyl unit. The reaction is not triggered in the absence of silane, so it must be the case that the reaction is triggered by electron transfer to the neutral benzyl group. 3-Electron bond formation between the radical component and the indolylmethyl anion gives a very electron-rich species, which could donate an electron to a molecule of the substrate. Fragmentation of the C-N bond, prototropy and work-up should then yield the product **29**.

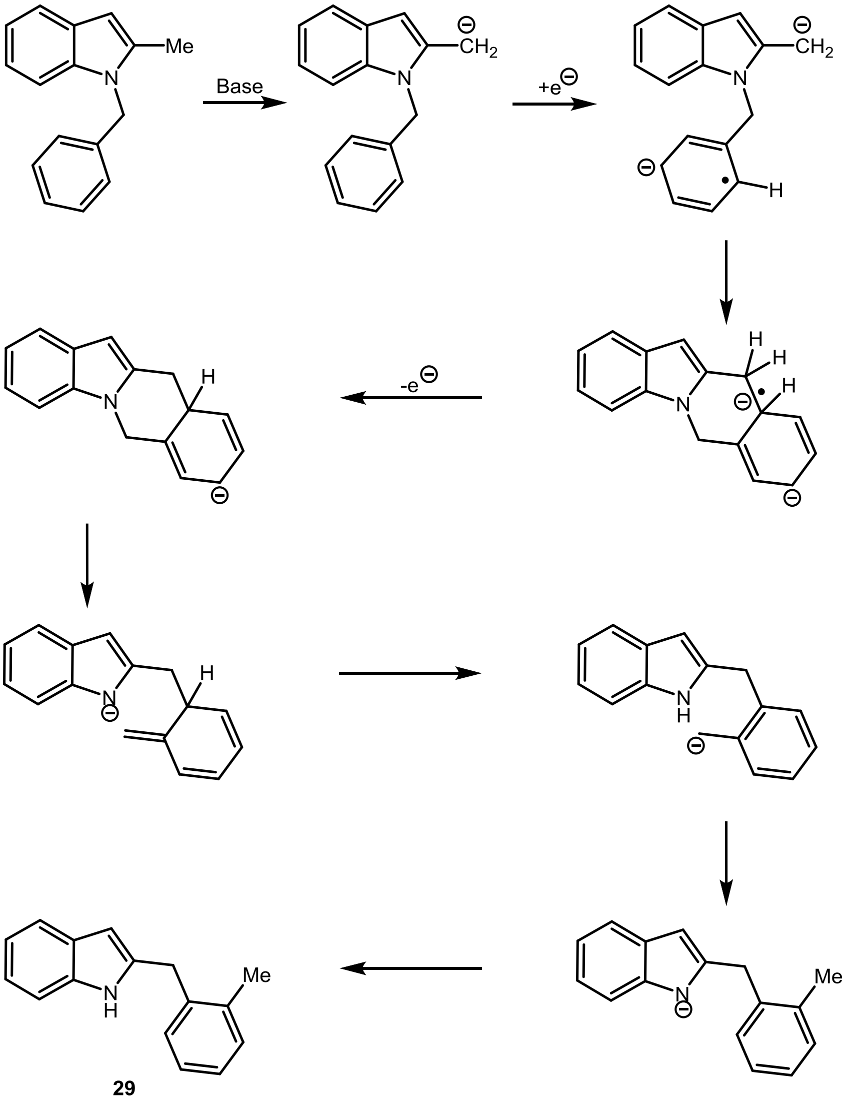

29

References

1 G. W. T. M. J. Frisch, H. B. Schlegel, G. E. Scuseria, M. A. Robb, J. R. Cheeseman, G. Scalmani, V. Barone, B. Mennucci, G. A. Petersson, H. Nakatsuji, M. Caricato, X. Li, H. P. Hratchian, A. F. Izmaylov, J. Bloino, G. Zheng, J. L. Sonnenberg, M. Hada, M. Ehara, K. Toyota, R. Fukuda, J. Hasegawa, M. Ishida, T. Nakajima, Y. Honda, O. Kitao, H. Nakai, T. Vreven, J. A. Montgomery, Jr., J. E. Peralta, F. Ogliaro, M. Bearpark, J. J. Heyd, E.

Brothers, K. N. Kudin, V. N. Staroverov, R. Kobayashi, J. Normand, K. Raghavachari, A. Rendell, J. C. Burant, S. S. Iyengar, J. Tomasi, M. Cossi, N. Rega, J. M. Millam, M. Klene, J. E. Knox, J. B. Cross, V. Bakken, C. Adamo, J. Jaramillo, R. Gomperts, R. E. Stratmann, O. Yazyev, A. J. Austin, R. Cammi, C. Pomelli, J. W. Ochterski, R. L. Martin, K. Morokuma, V. G. Zakrzewski, G. A. Voth, P. Salvador, J. J. Dannenberg, S. Dapprich, A. D. Daniels, O. Farkas, J. B. Foresman, J. V. Ortiz, J. Cioslowski, and D. J. Fox, Gaussian, Inc., Wallingford CT, **2009**.

2 Y. Zhao and D. G. Truhlar, *Theor. Chem. Acc.*, 2008, **120**, 215–241.

3 V. A. Rassolov, M. A. Ratner, J. A. Pople, P. C. Redfern and L. A. Curtiss, *J. Comput. Chem.*, 2001, **22**, 976–984.

4 M. Cossi, N. Rega, G. Scalmani and V. Barone, *J. Comput. Chem.*, 2003, **24**, 669–681.

5 A. P. Altshuller and L. Rosenblum, *J. Am. Chem. Soc.*, 1955, **77**, 272–274.

6 R. A. Marcus, *J. Chem. Phys.*, 1965, **43**, 679–701.

7 S. F. Nelsen, S. C. Blackstock and Y. Kim, *J. Am. Chem. Soc.*, 1987, **109**, 677–682.

8 H. G. Roth, N. A. Romero and D. A. Nicewicz, *Synlett*, 2016, **27**, 714–723.

9 K. Nemoto, S. Tanaka, M. Konno, S. Onozawa, M. Chiba, Y. Tanaka, Y. Sasaki, R. Okubo and T. Hattori, *Tetrahedron*, 2016, **72**, 734–745.

10 I. Buder, G. Schwitzgebel, S. Samsoniya, E. Gogritchiani and I. Chikvaidze, *Chem. Heterocycl. Compd.*, 2005, **41**, 1121–1129.

11 Y.-M. Su, Y. Hou, F. Yin, Y.-M. Xu, Y. Li, X. Zheng and X.-S. Wang, *Org. Lett.*, 2014, **16**, 2958–2961.

12 Y.-Q.-Q. Yi, W.-C. Yang, D.-D. Zhai, X.-Y. Zhang, S.-Q. Li and B.-T. Guan, *Chem. Commun.*, 2016, **52**, 10894–10897.

13 S. R. Kandukuri, J. A. Schiffner and M. Oestreich, *Angew. Chem. Int. Ed.*, 2012, **51**, 1265–1269.

14 R. Yang, J.-T. Yu, S. Sun, Q. Zheng and J. Cheng, *Tetrahedron Lett.*, 2017,

58, 445–448.

15 J. Ghorai, A. C. S. Reddy and P. Anbarasan, *Chem. Eur. J.*, 2016, **22**, 16042–16046.

16 M. Taddei, M. G. Mura, S. Rajamäki, L. De Luca and A. Porcheddu, *Adv. Synth. Catal.*, 2013, **355**, 3002–3013.

17 T. Hensel, D. Trpcevski, C. Lind, R. Grosjean, P. Hammershøj, C. B. Nielsen, T. Brock-Nannestad, B. E. Nielsen, M. Schau-Magnussen, B. Minaev, G. V. Baryshnikov and M. Pittelkow, *Chem. Eur. J.*, 2013, **19**, 17097–17102.

18 E. Tayama, M. Ishikawa, H. Iwamoto and E. Hasegawa, *Tetrahedron Lett.*, 2012, **53**, 5159–5161.

19 J. Barluenga, F. J. Fañanás, R. Sanz and Y. Fernández, *Chem. Eur. J.*, 2002, **8**, 2034–2046.

20 W. Jian, L. Ge, Y. Jiao, B. Qian and H. Bao, *Angew. Chem. Int. Ed.*, 2017, **56**, 3650–3654.

21 M. Guo, S. Liu, S. Chen, Y. Wen, H. Liang and M. Lv, *Synth. Commun.*, 2015, **45**, 767–777.

22 A. R. Hajipour, S. M. Hosseini and F. Mohammadsaleh, *New J. Chem.*, 2016, **40**, 6939–6945.

23 F. Nador, Y. Moglie, C. Vitale, M. Yus, F. Alonso and G. Radivoy, *Tetrahedron*, 2010, **66**, 4318–4325.

24 V. Kanchupalli, D. Joseph and S. Katukojvala, *Org. Lett.*, 2015, **17**, 5878–5881.

25 C. M. Griffiths-Jones (née Haskins) and D. W. Knight, *Tetrahedron*, 2011, **67**, 8515–8528.

26 M. D. L. Tonin, D. Zell, V. Müller and L. Ackermann, *Synthesis (Stuttg).*, 2017, **49**, 127–134.

27 D.-S. Wang, Q.-A. Chen, W. Li, C.-B. Yu, Y.-G. Zhou and X. Zhang, *J. Am. Chem. Soc.*, 2010, **132**, 8909–8911.

28 I. Choi, H. Chung, J. W. Park and Y. K. Chung, *Org. Lett.*, 2016, **18**, 5508–5511.

29 T. Chatterjee, G. Roh, M. A. Shoaib, C.-H. Suhl, J. S. Kim, C.-G. Cho and E. J. Cho, *Org. Lett.*, 2017, **19**, 1906–1909.

30 Q. Yan, E. Gin, M. Wasinska-Kalwa, M. G. Banwell and P. D. Carr, *J. Org. Chem.*, 2017, **82**, 4148–4159.

31 *http//sdbs.db.aist.go.jp/sdbs/cgi-bin/direct_frame_top.cgi, April 2017*.

32 T. You, Z. Wang, J. Chen and Y. Xia, *J. Org. Chem.*, 2017, **82**, 1340–1346.

33 C. Lombardi, J. Day, N. Chandrasoma, D. Mitchell, M. J. Rodriguez, J. L. Farmer and M. G. Organ, *Organometallics*, 2017, **36**, 251–254.

34 H.-G. Cheng, L.-Q. Lu, T. Wang, Q.-Q. Yang, X.-P. Liu, Y. Li, Q.-H. Deng, J.-R. Chen and W.-J. Xiao, *Angew. Chem. Int. Ed.*, 2013, **52**, 3250–3254.

35 S. Gore, S. Baskaran and B. König, *Org. Lett.*, 2012, **14**, 4568–4571.

36 A. Fürstner, R. Martin, H. Krause, G. Seidel, R. Goddard and C. W. Lehmann, *J. Am. Chem. Soc.*, 2008, **130**, 8773–8787.

37 S. Kitagaki, M. Kajita, S. Narita and C. Mukai, *Org. Lett.*, 2012, **14**, 1366–1369.

38 N. J. Findlay, S. R. Park, F. Schoenebeck, E. Cahard, S. Zhou, L. E. A. Berlouis, M. D. Spicer, T. Tuttle and J. A. Murphy, *J. Am. Chem. Soc.*, 2010, **132**, 15462–15464.

39 O. Lebedev and U. Mäeorg, *Organometallics*, 2014, **33**, 188–193.

40 Y. Zhu, T. Xiong, W. Han and Y. Shi, *Org. Lett.*, 2014, **16**, 6144–6147.

41 K. Sato, Y. Inoue, T. Mori, A. Sakaue, A. Tarui, M. Omote, I. Kumadaki and A. Ando, *Org. Lett.*, 2014, **16**, 3756–3759.

Spectra

Substrates

1-Benzyl-1*H*-indole (15)

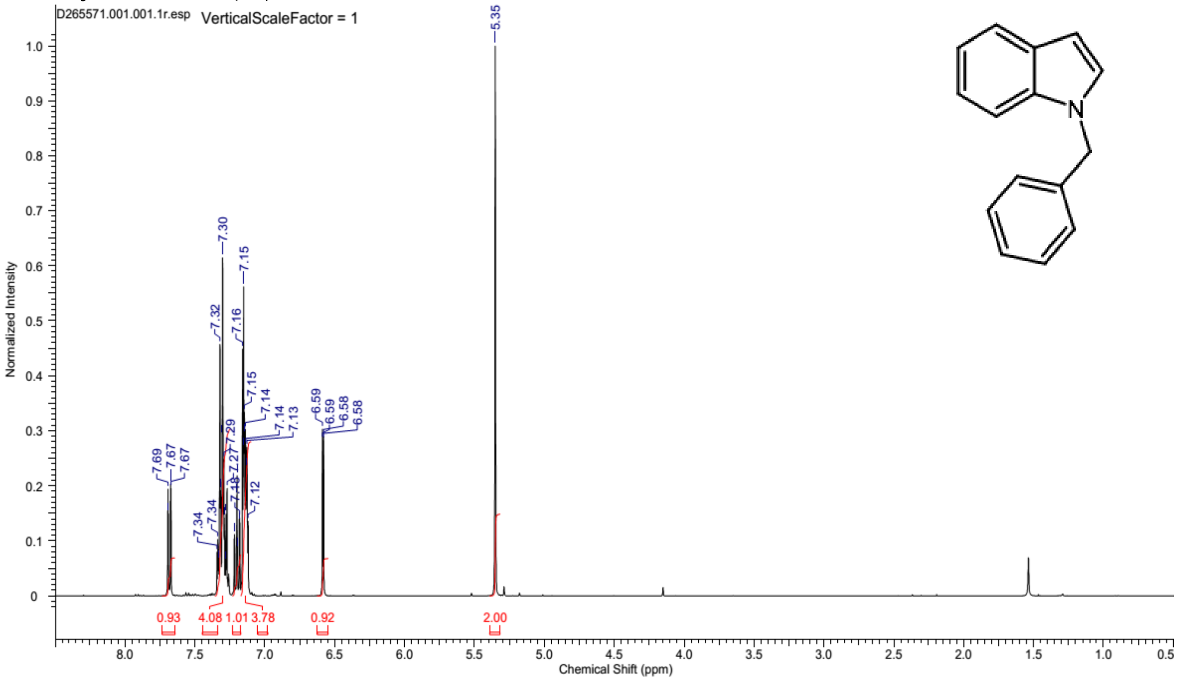

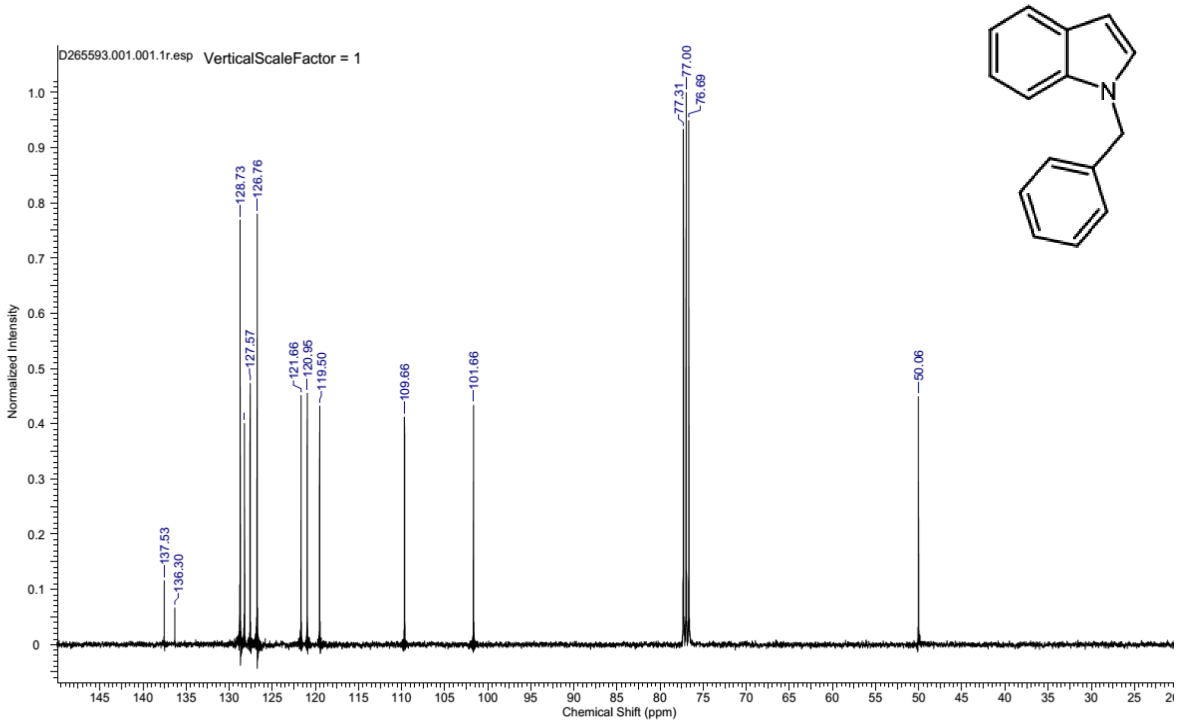

1-Benzyl-2-methyl-1*H*-indole (16)

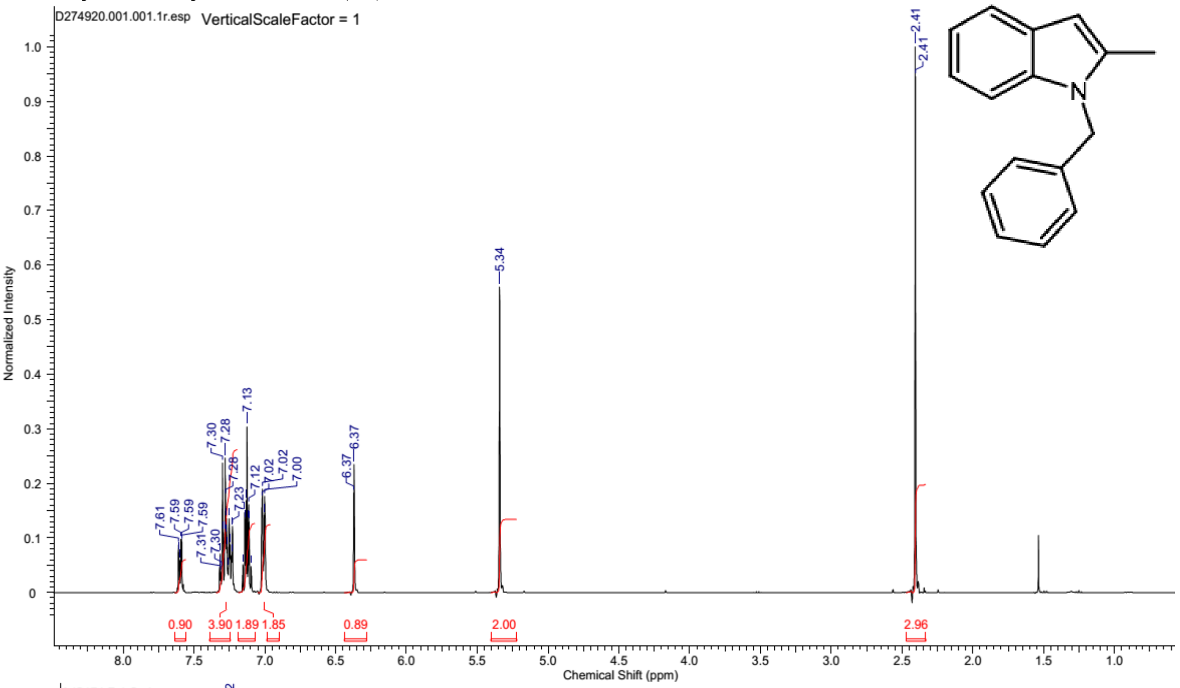

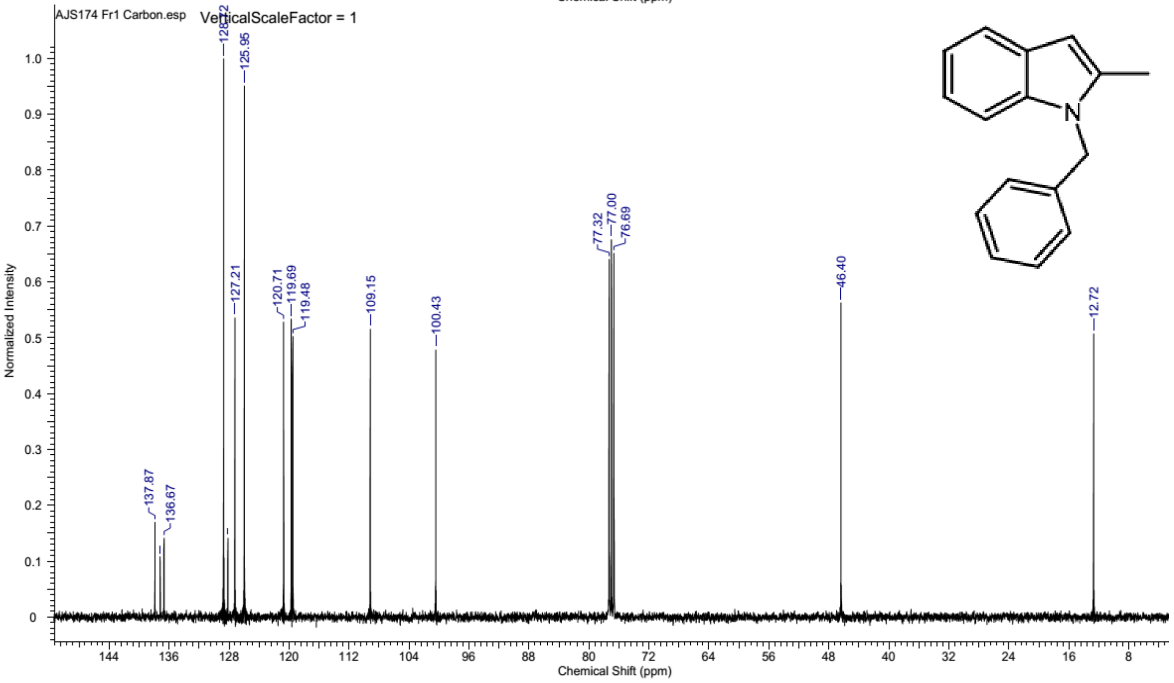

1-Benzyl-3-methyl-1*H*-indole (17)

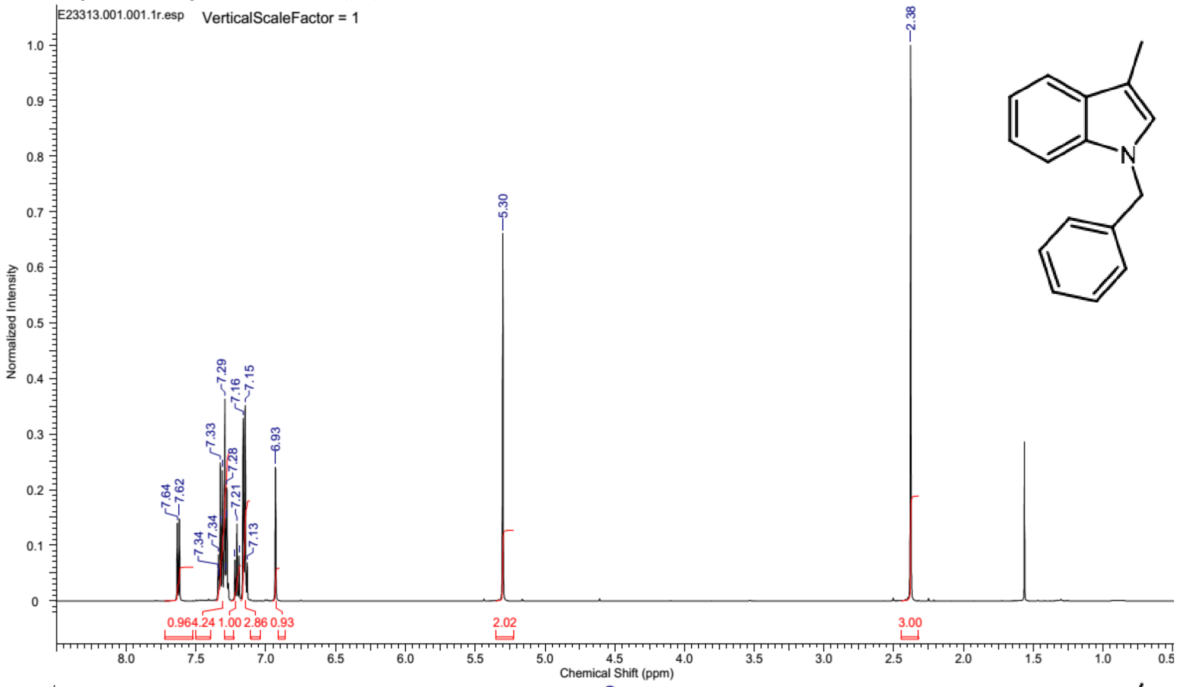

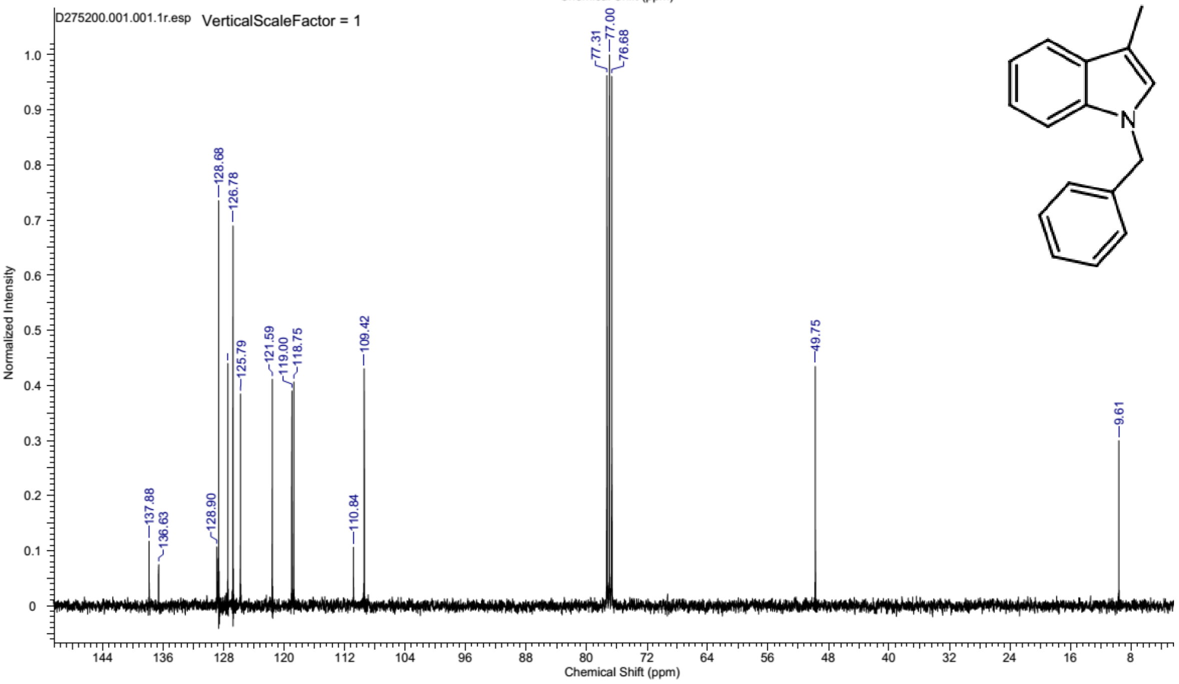

1-(4-Methoxybenzyl)-3-methyl-1*H*-indole (18)

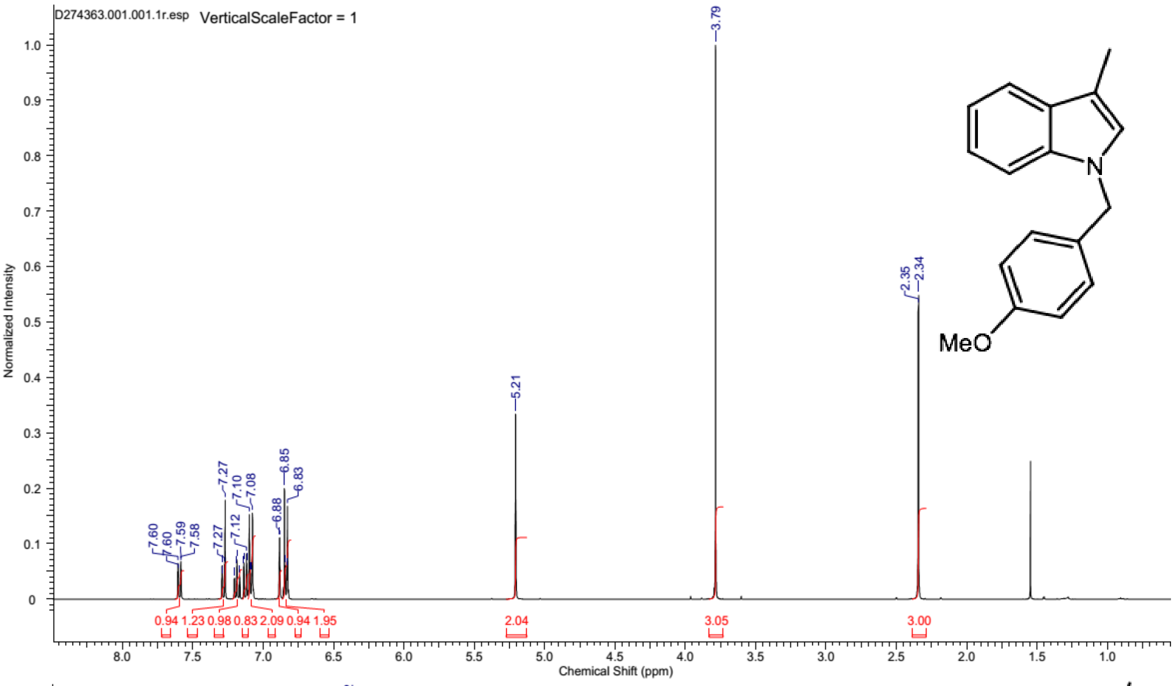

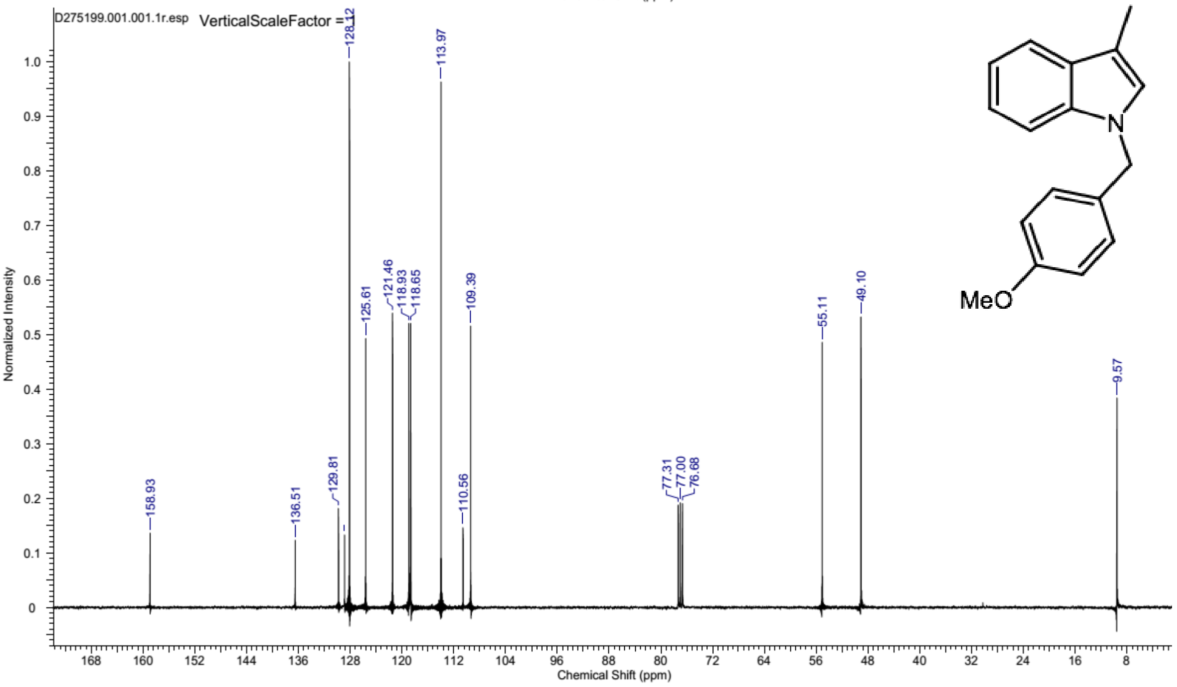

3-Methyl-1-(4-methylbenzyl)-1*H*-indole (19)

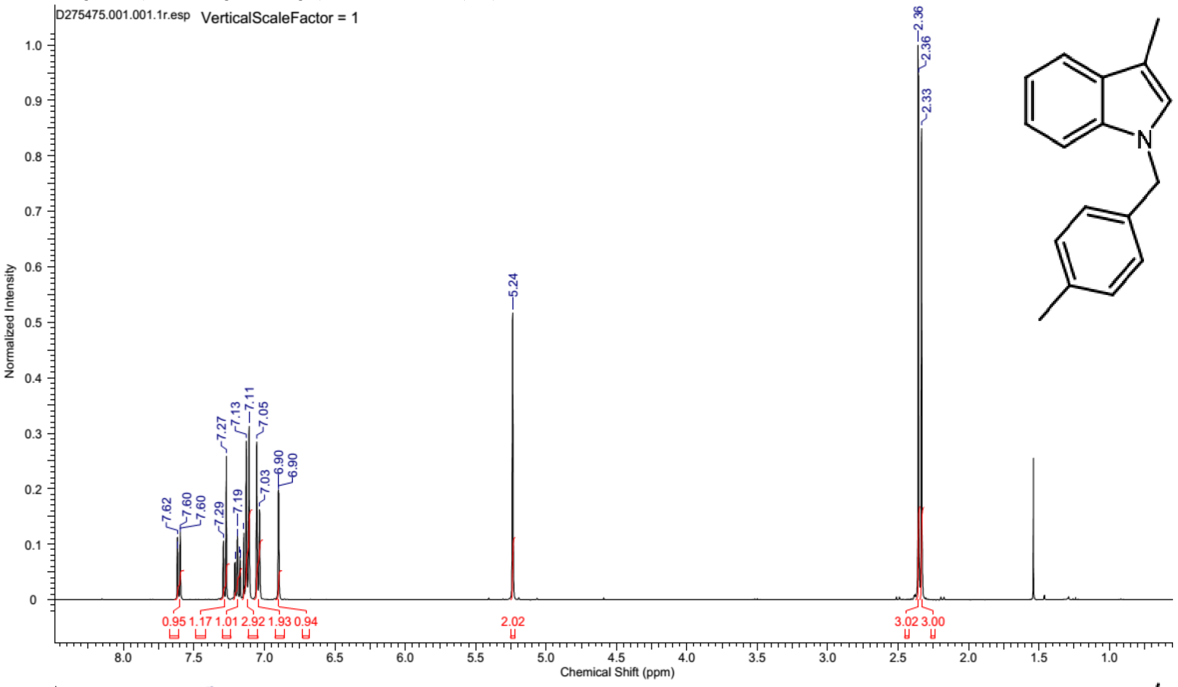

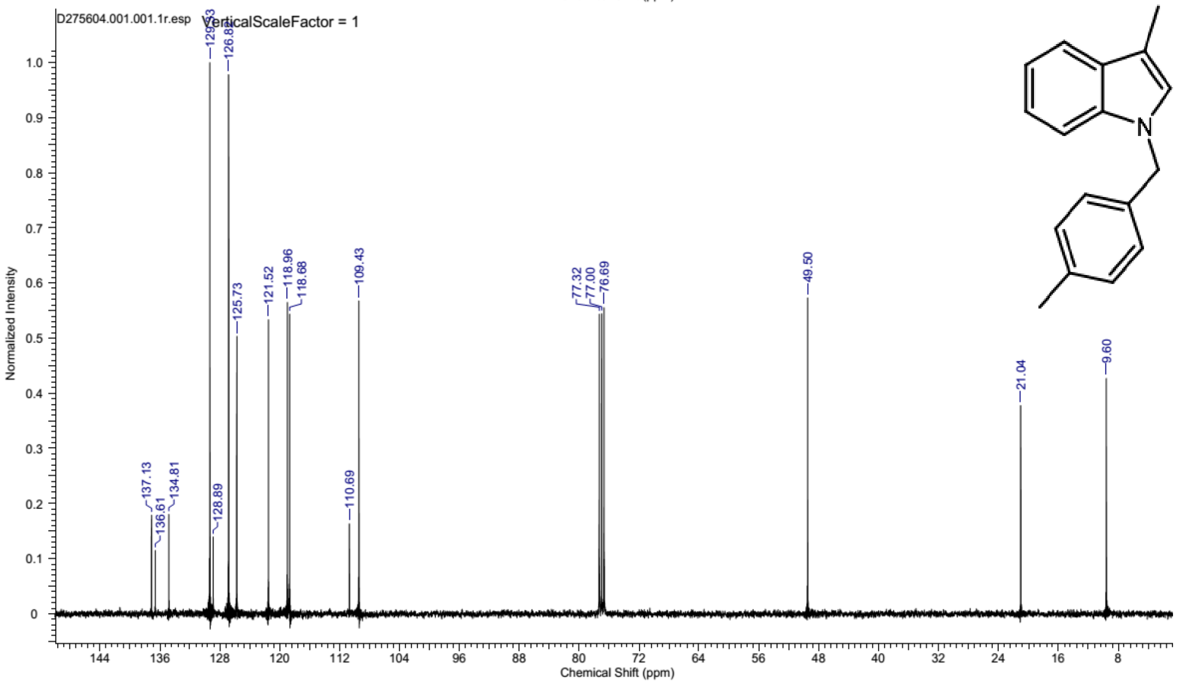

3-Methyl-1-(1-phenylethyl)-1*H*-indole (20)

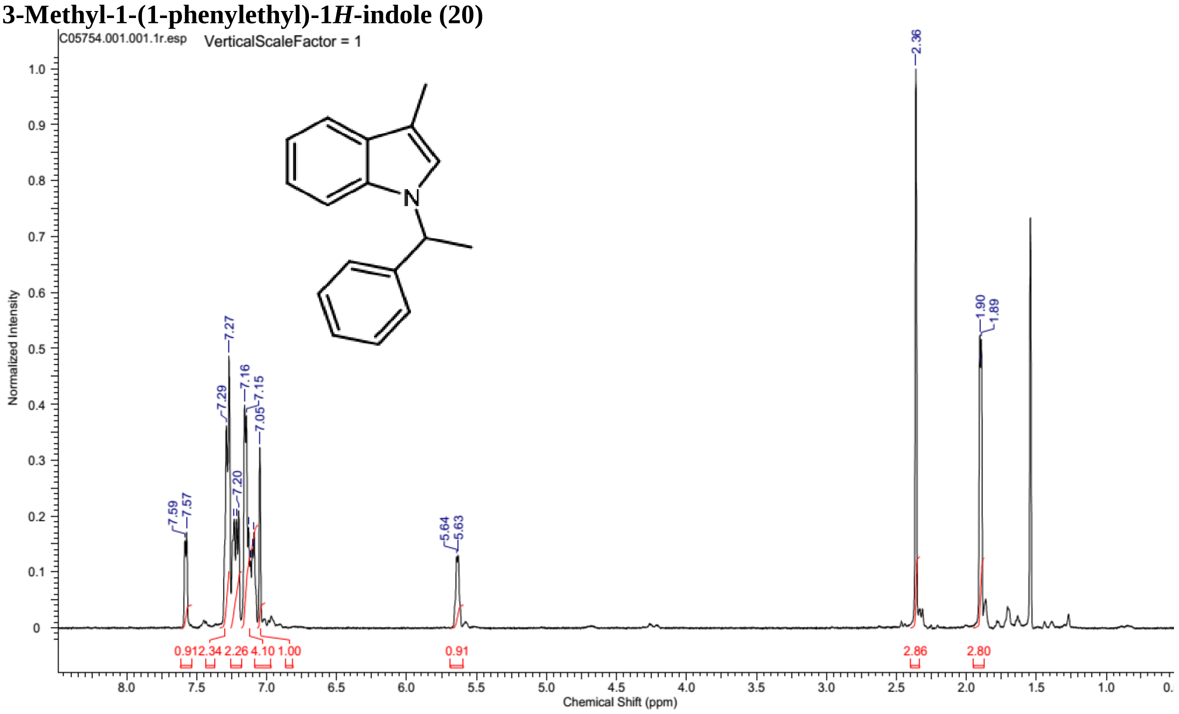

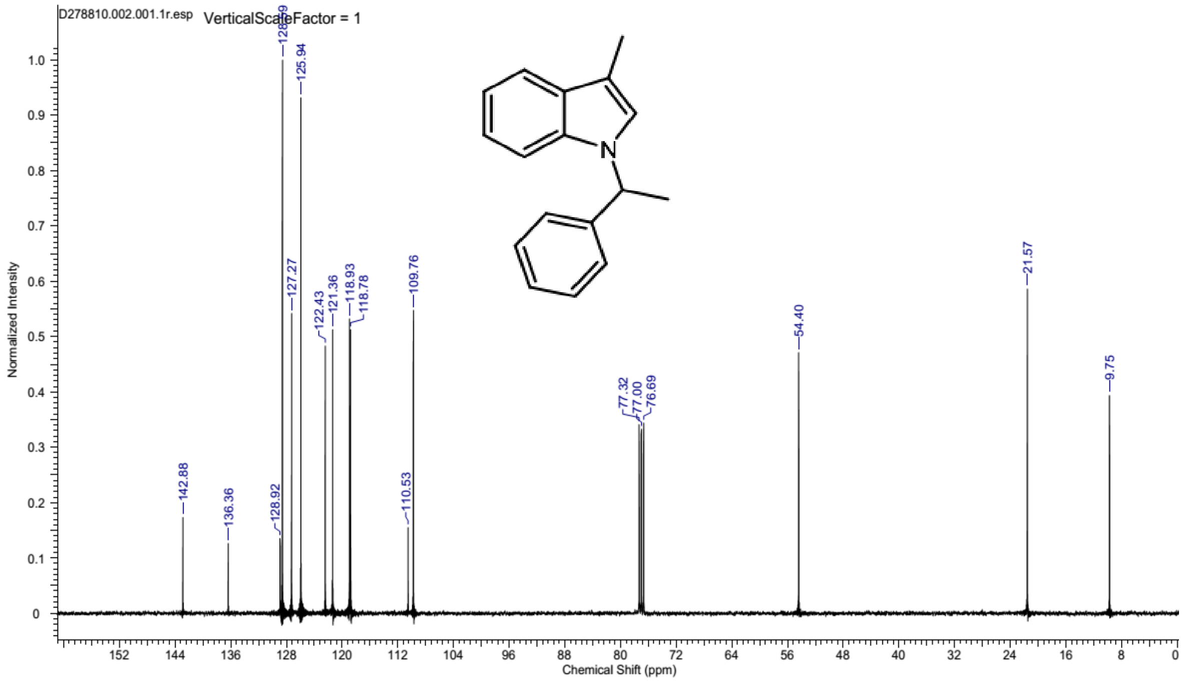

3-Phenyl-1*H*-indole (27)

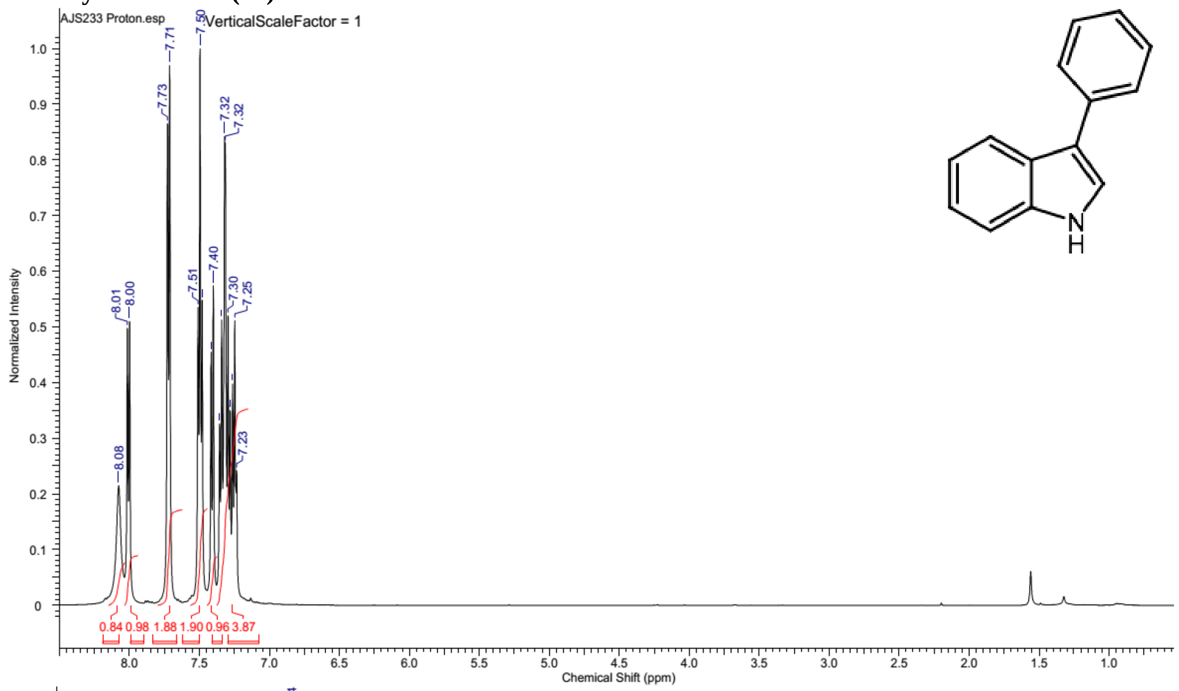

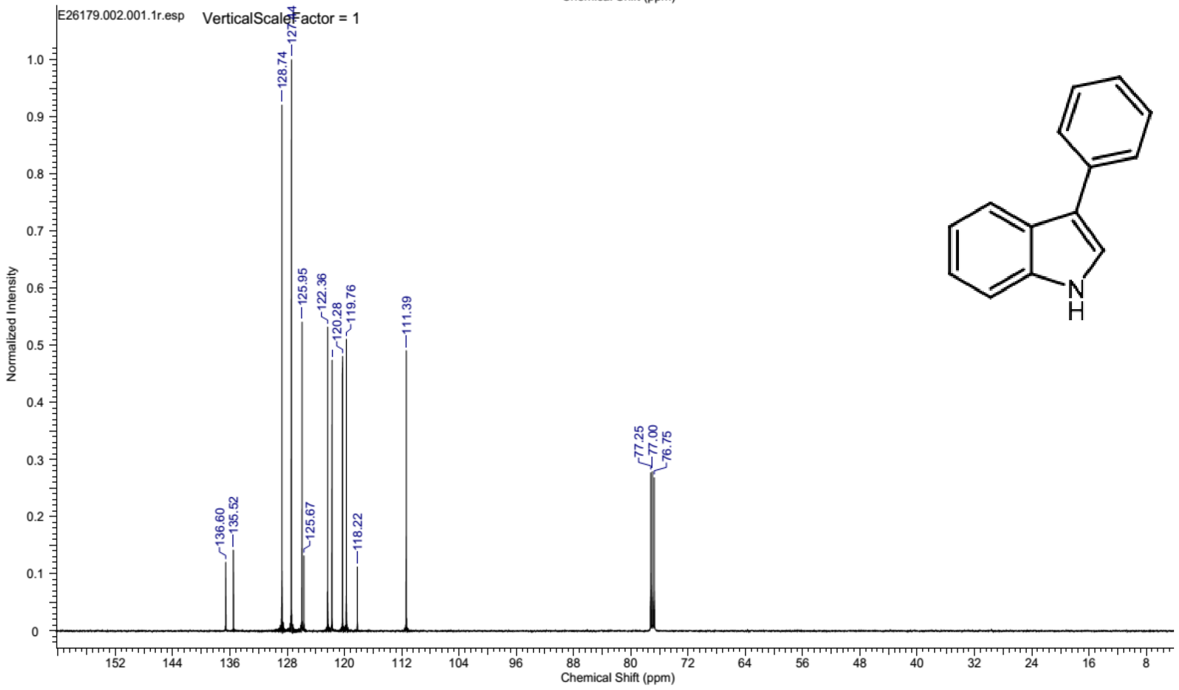

1-Benzyl-3-phenyl-1*H*-indole (21)

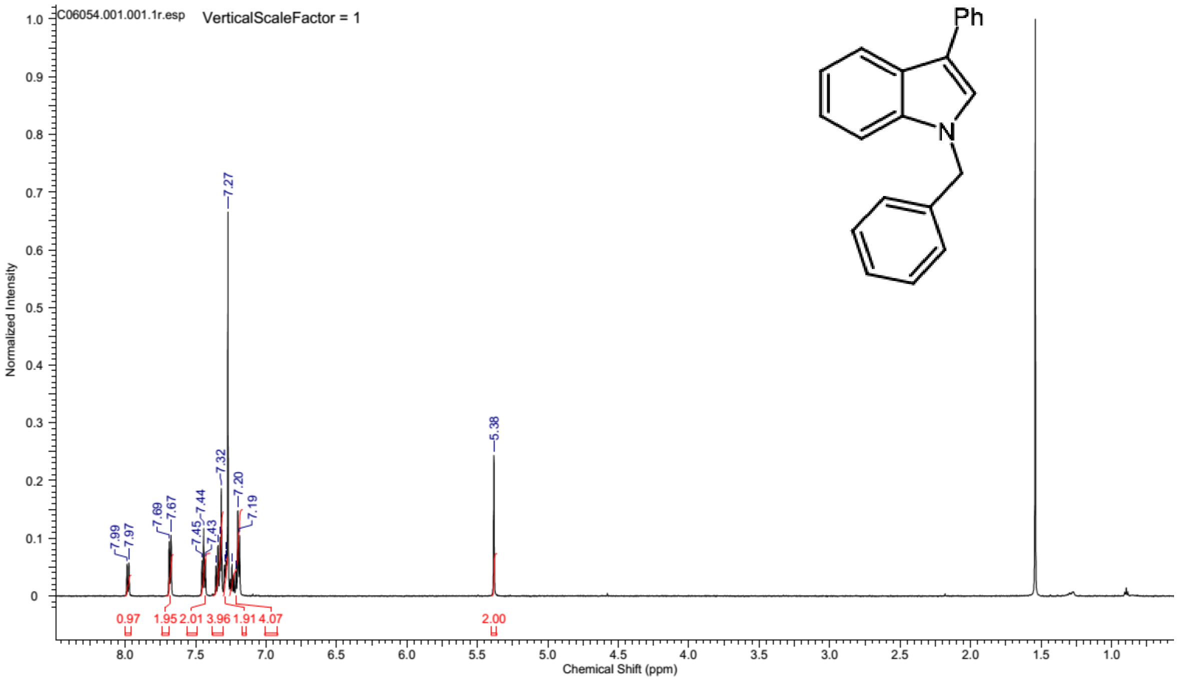

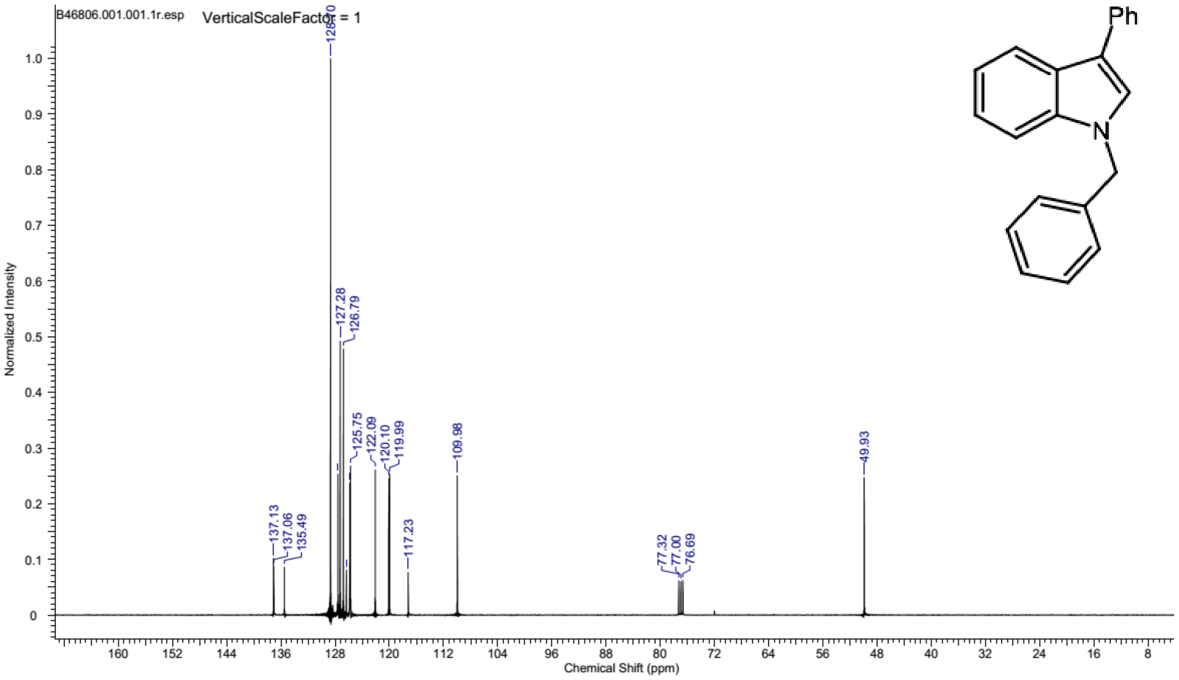

9-Benzyl-9*H*-carbazole (22)

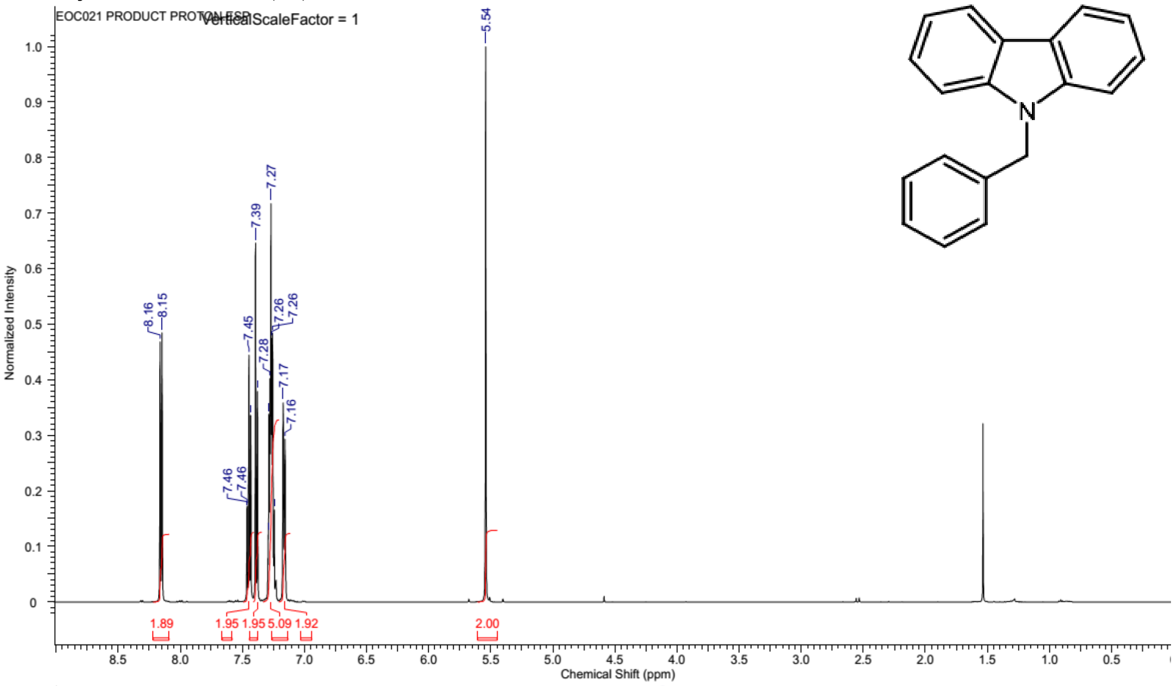

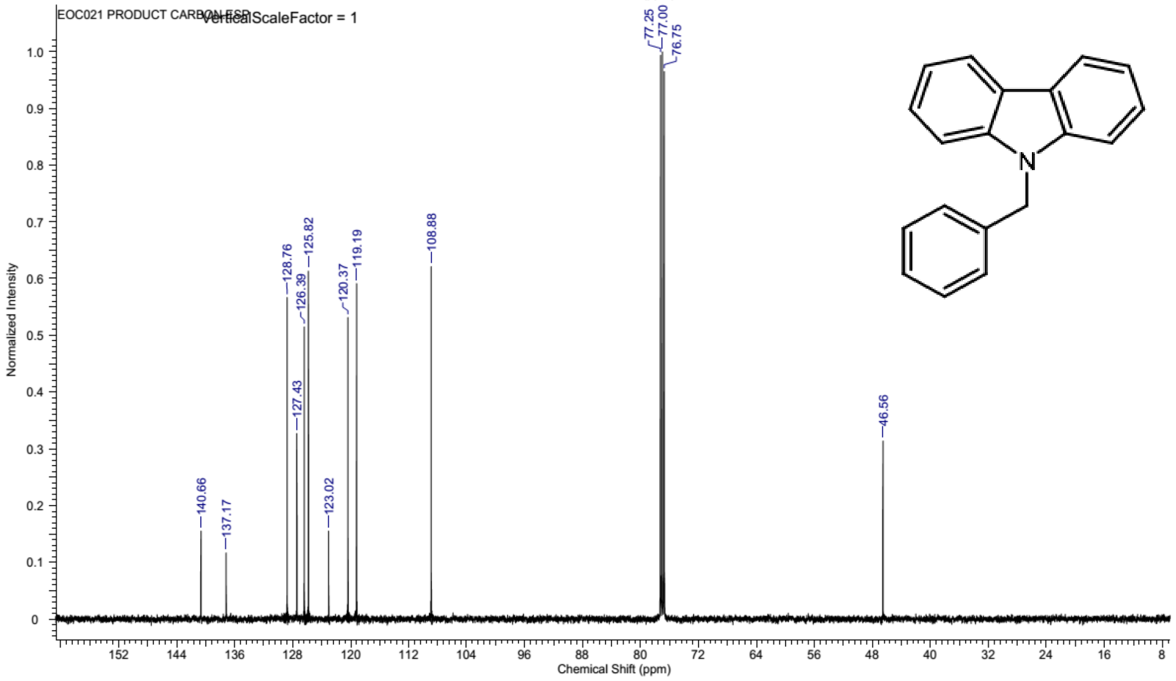

3-Methyl-1-(naphthalen-1-ylmethyl)-1*H*-indole (23)

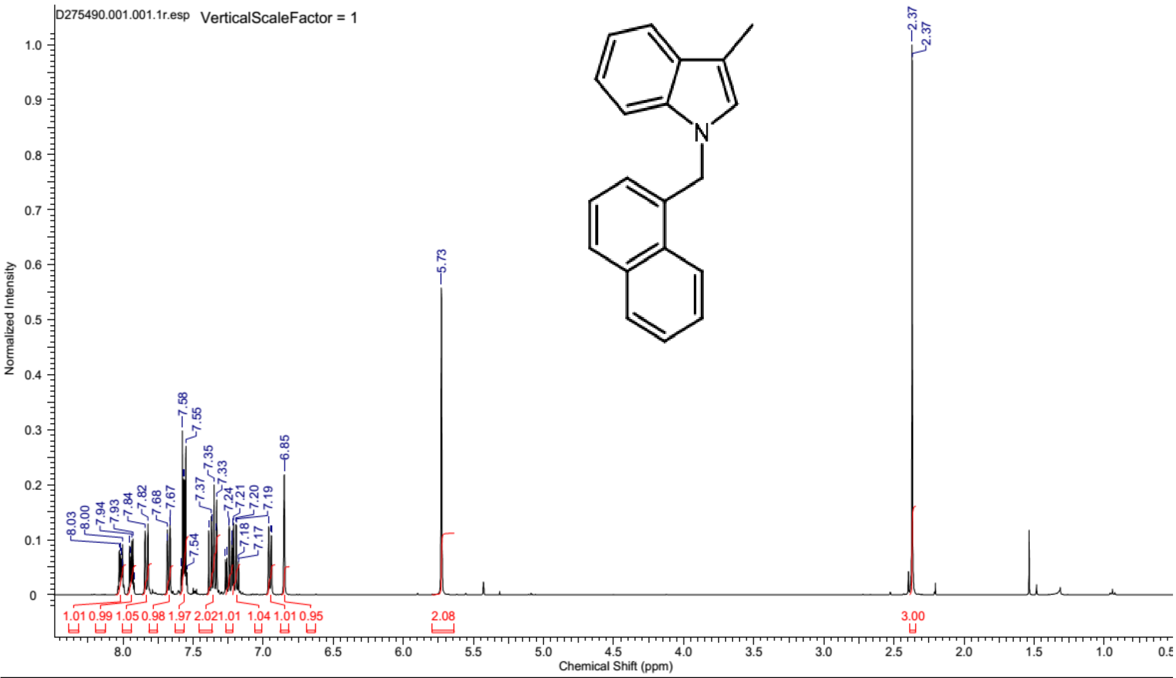

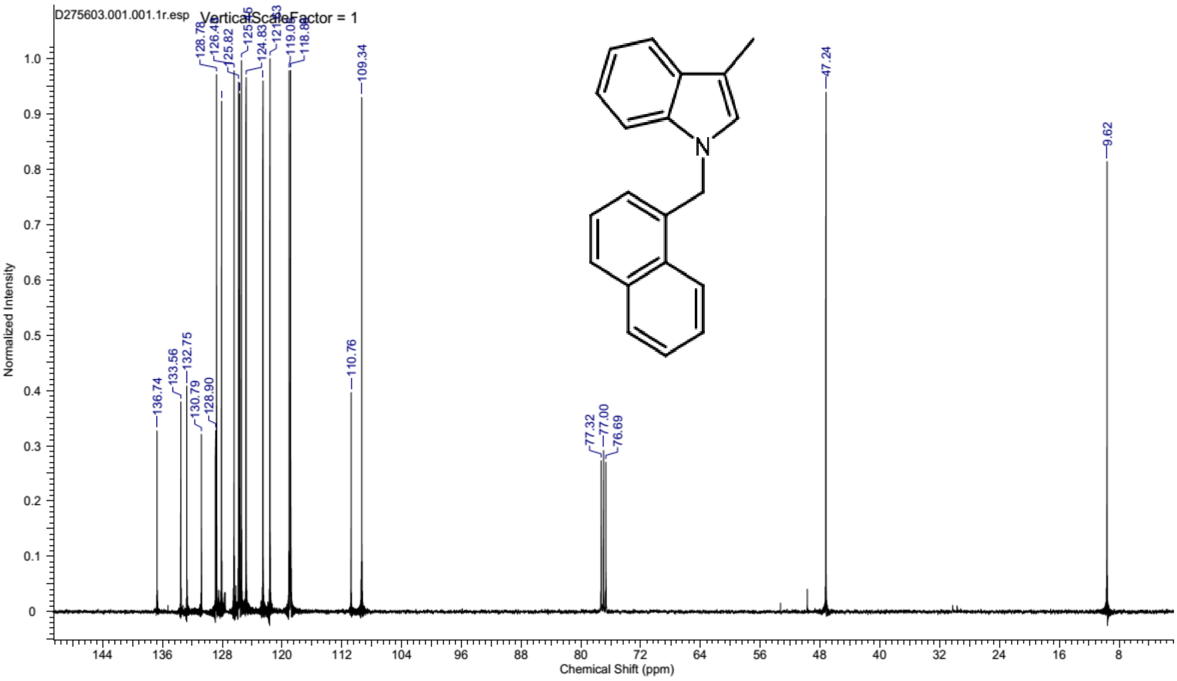

N-Benzyl-N-methylaniline (40)

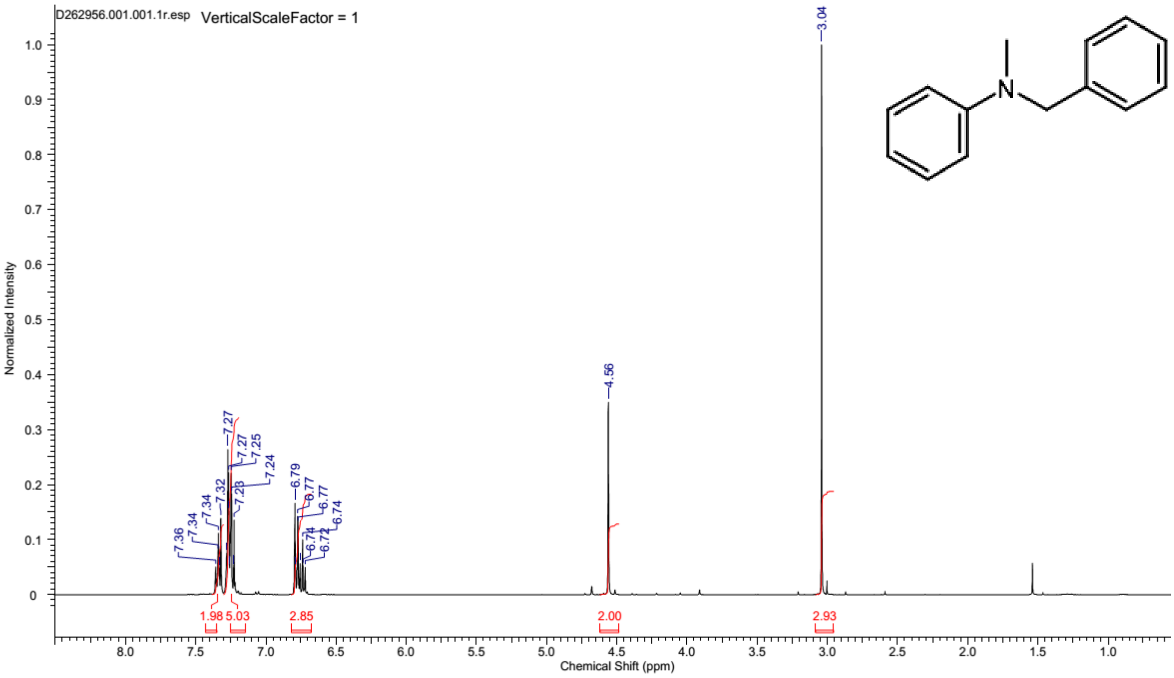

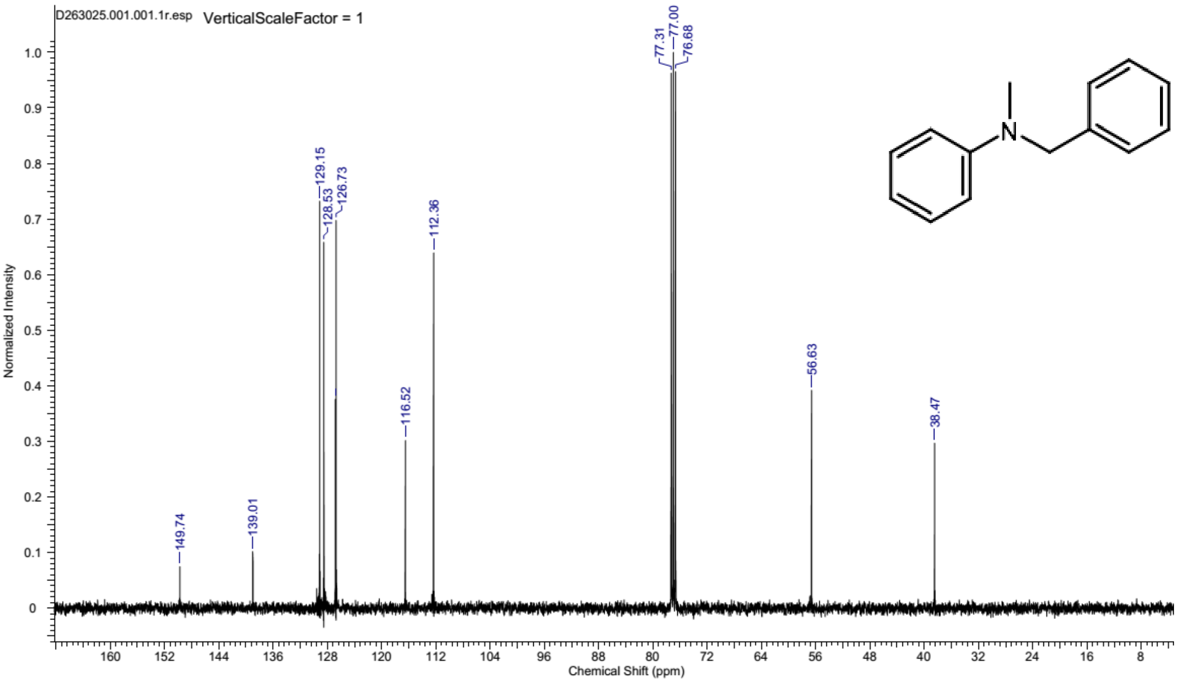

1-Allyl-3-methyl-1*H*-indole (43)

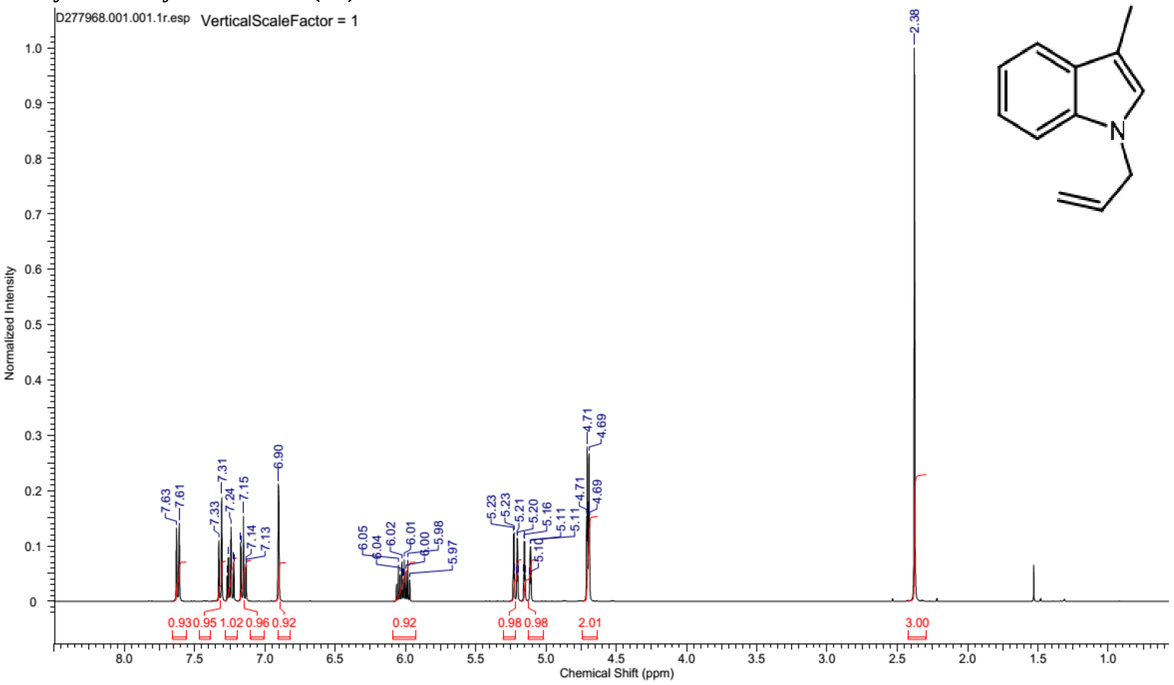

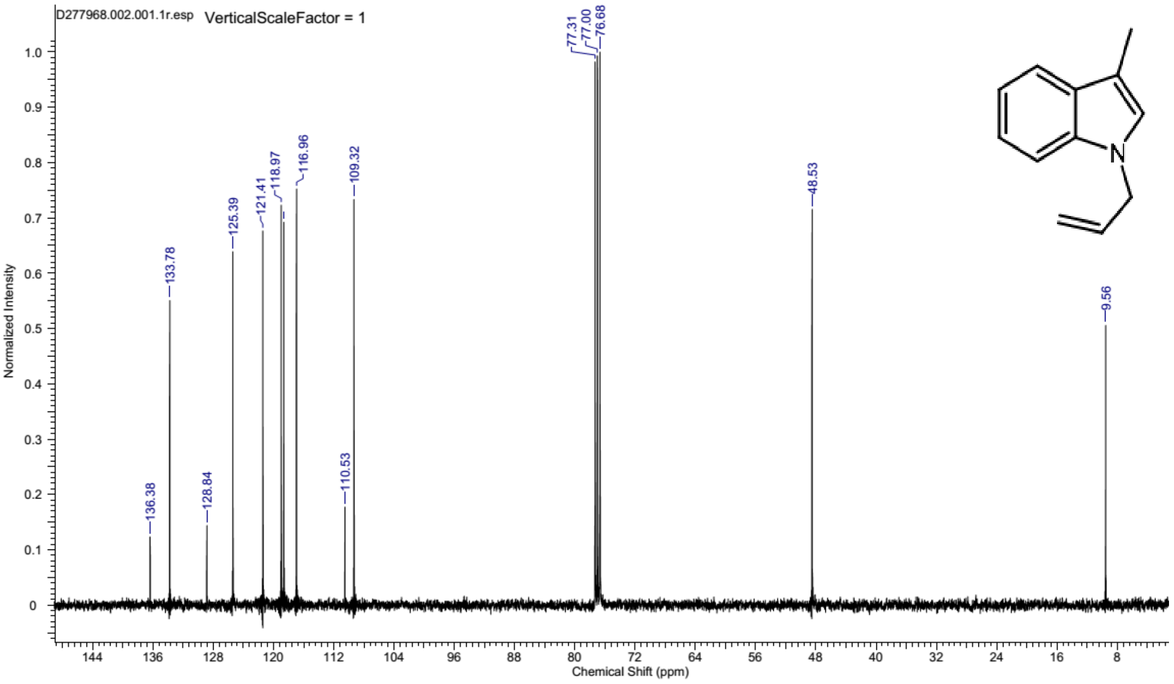

1-Allyl-2,3-dimethyl-1*H*-indole (45)

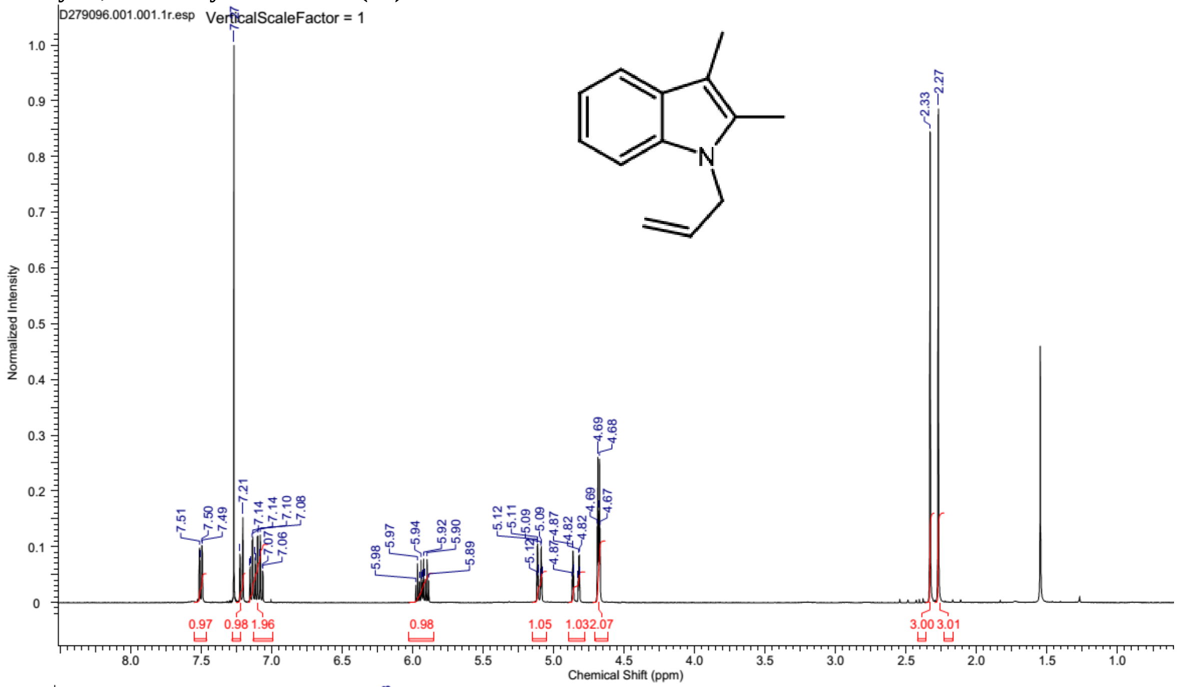

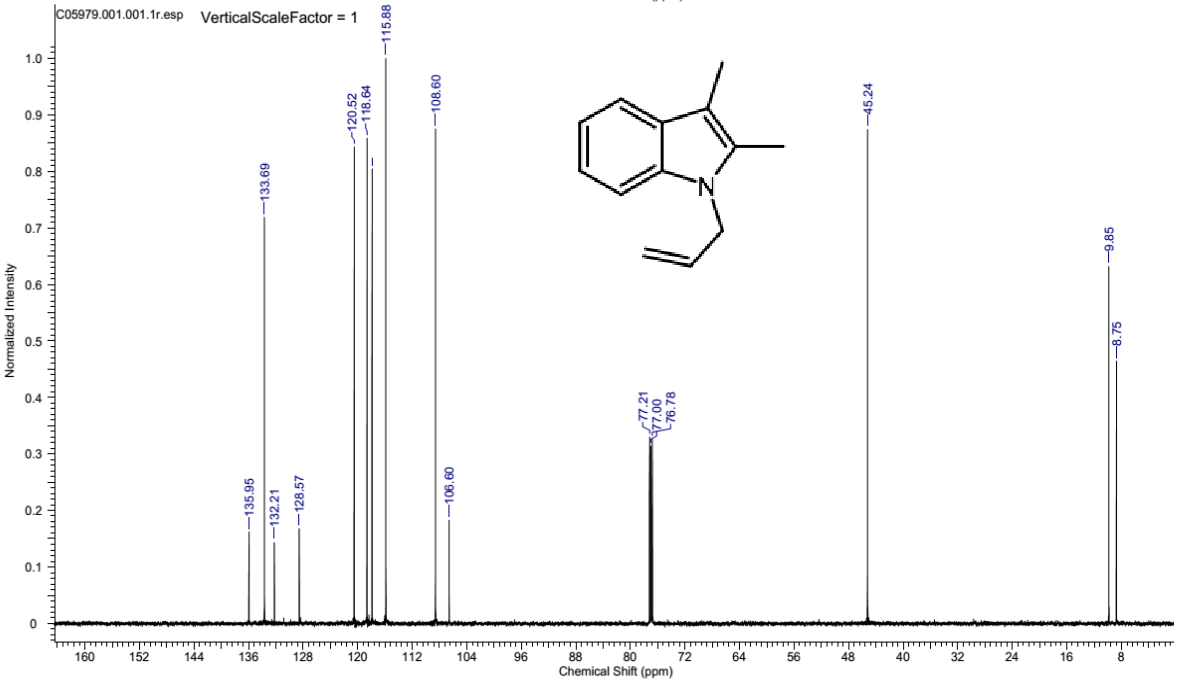

2-Phenyloctan-2-ol

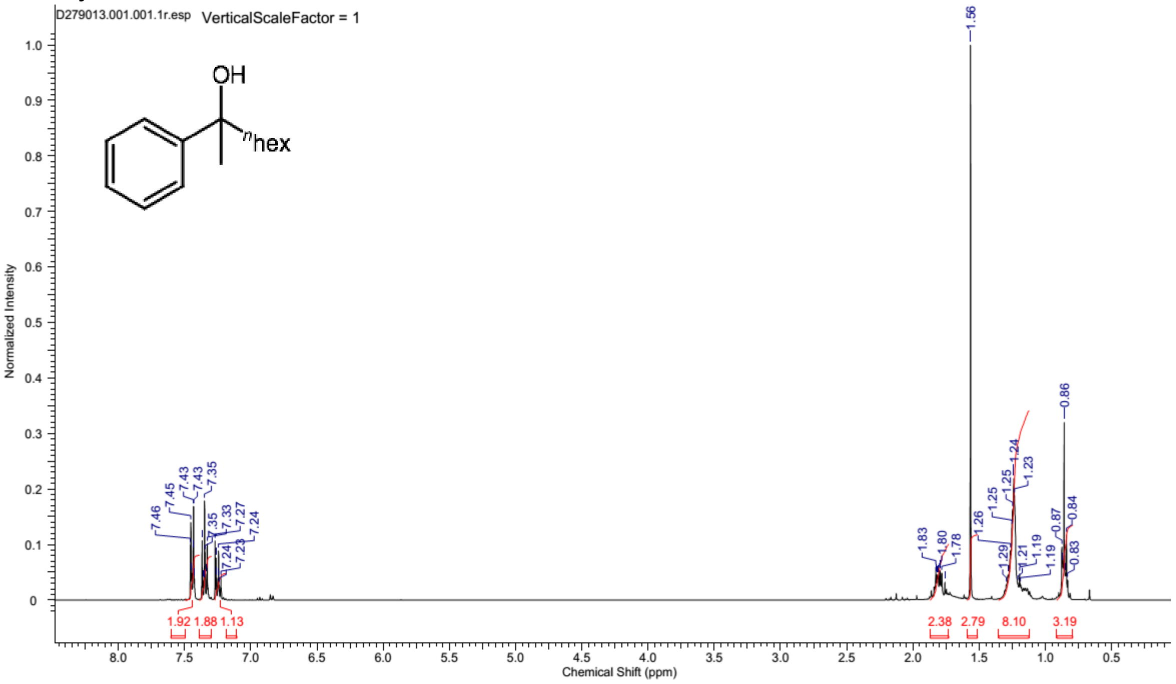

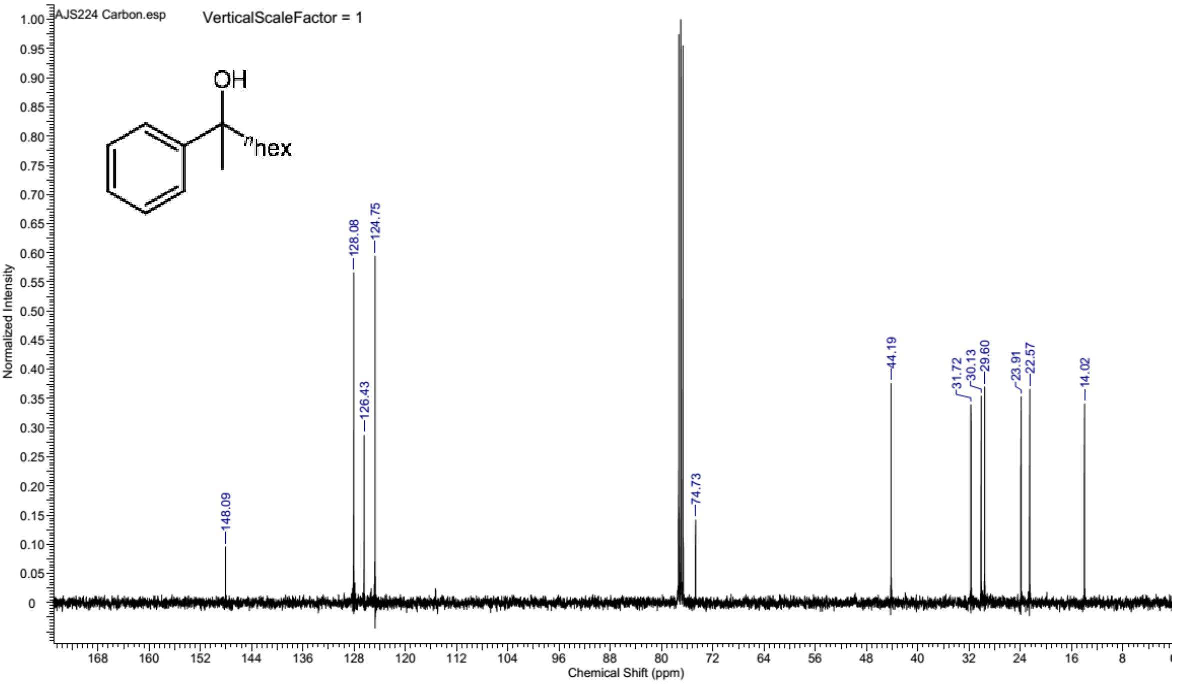

(2-Methoxyoctan-2-yl)benzene (47)

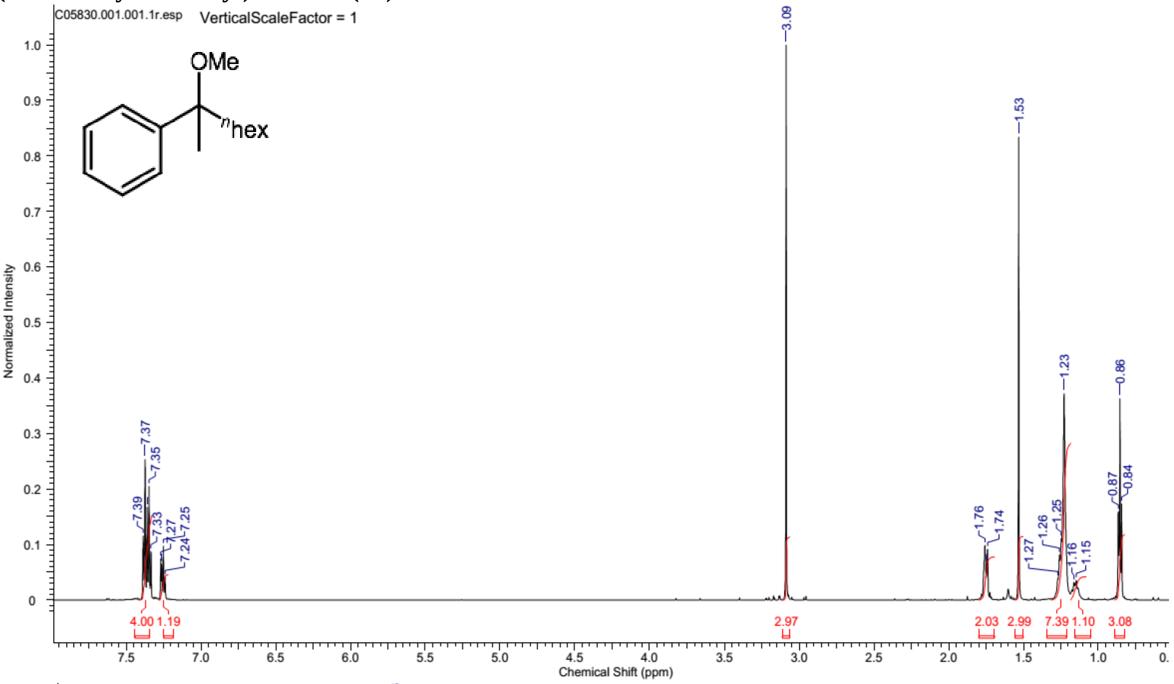

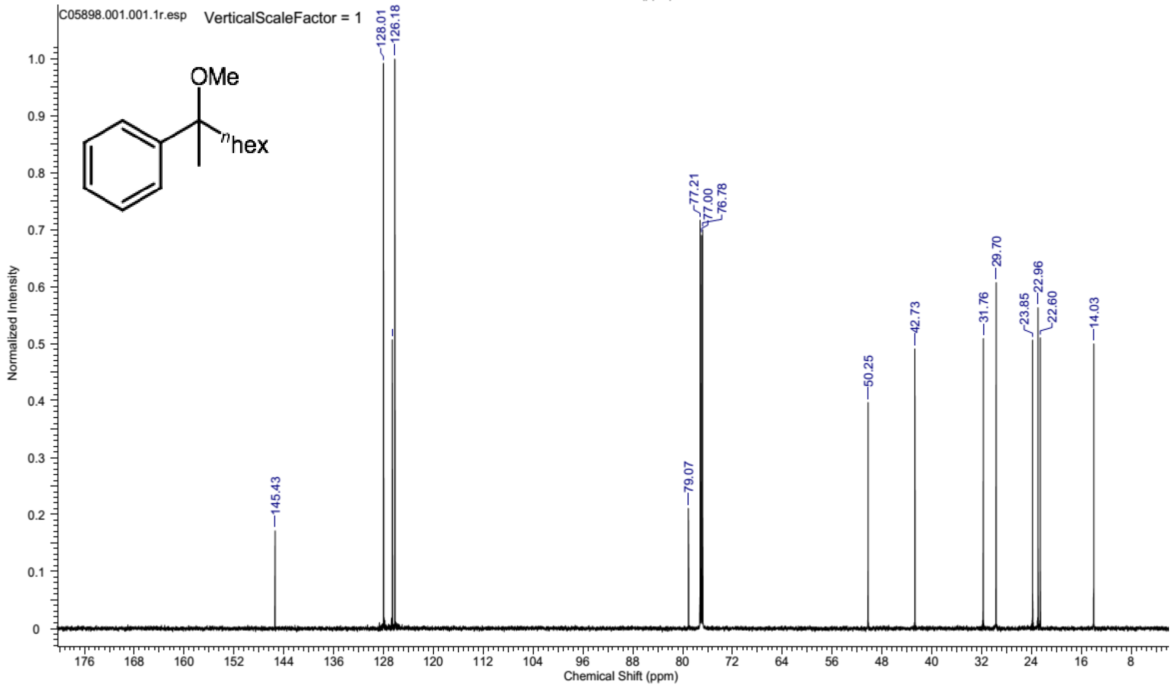

2-Phenyl-2-propylpentanenitrile (49)

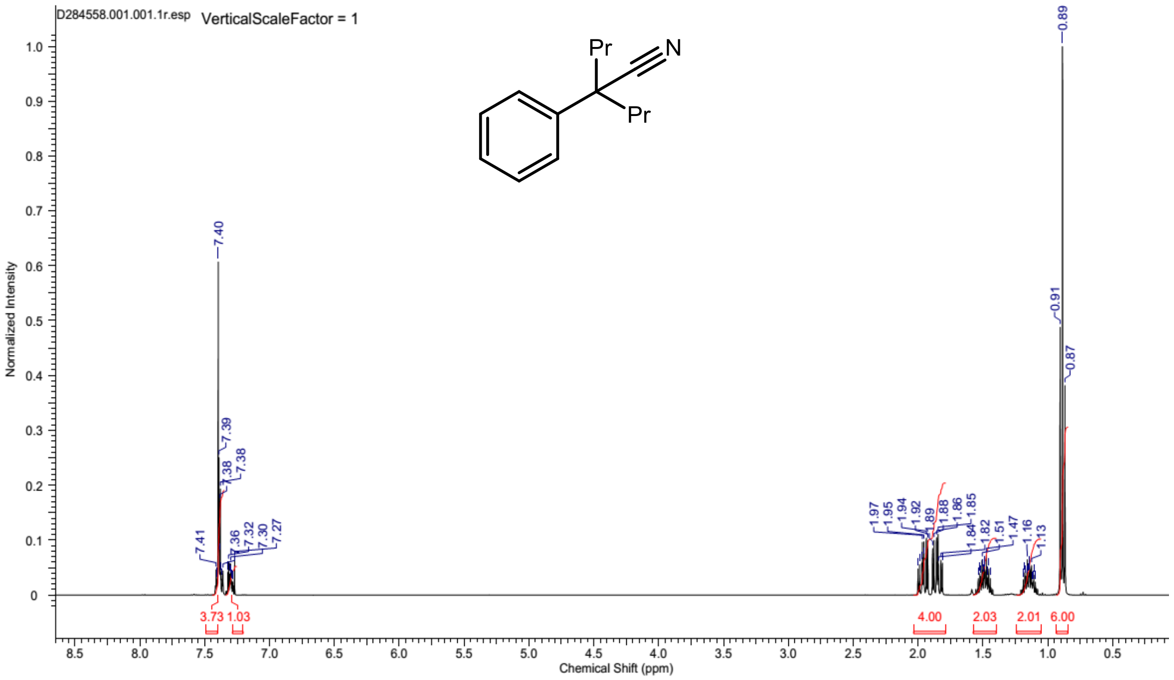

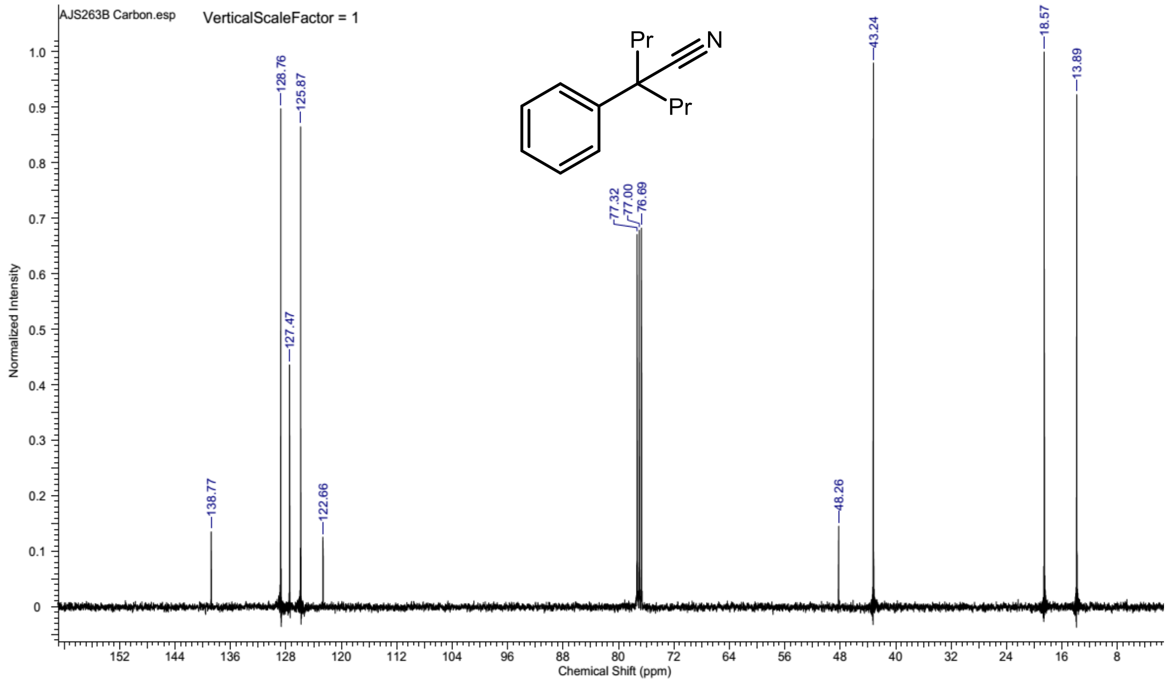

1,2-Diphenylethyne (54)

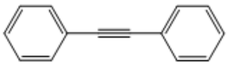

^{1}H NMR (400 MHz, Chloroform-*d*) δ 7.60 – 7.52 (m, 4H), 7.42 – 7.31 (m, 6H).

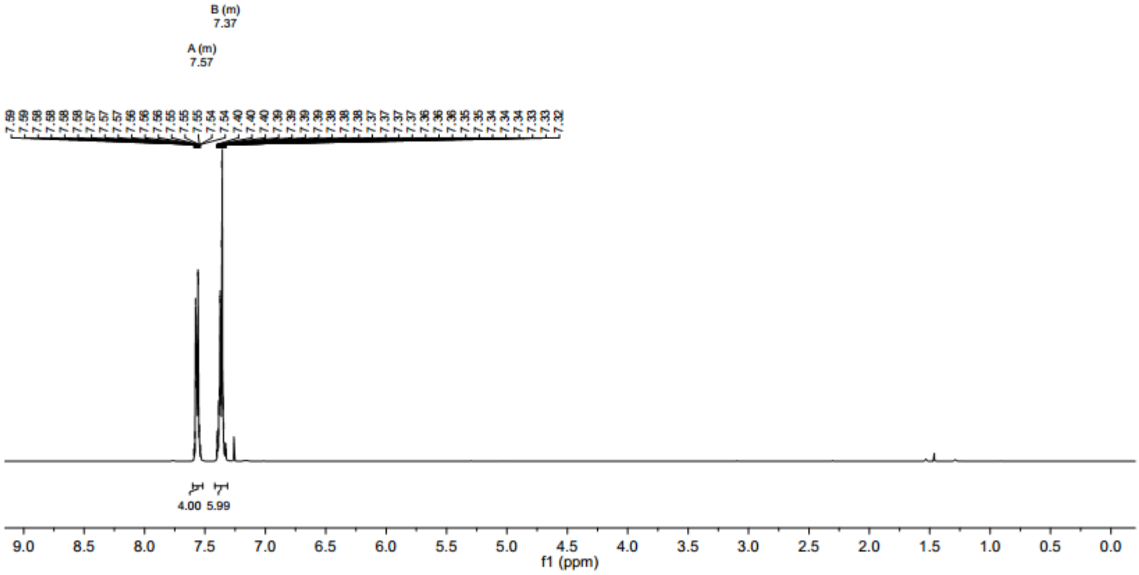

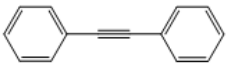

^{13}C NMR (101 MHz, $CDCl_3$) δ 131.74, 128.47, 128.38, 123.42, 89.52.

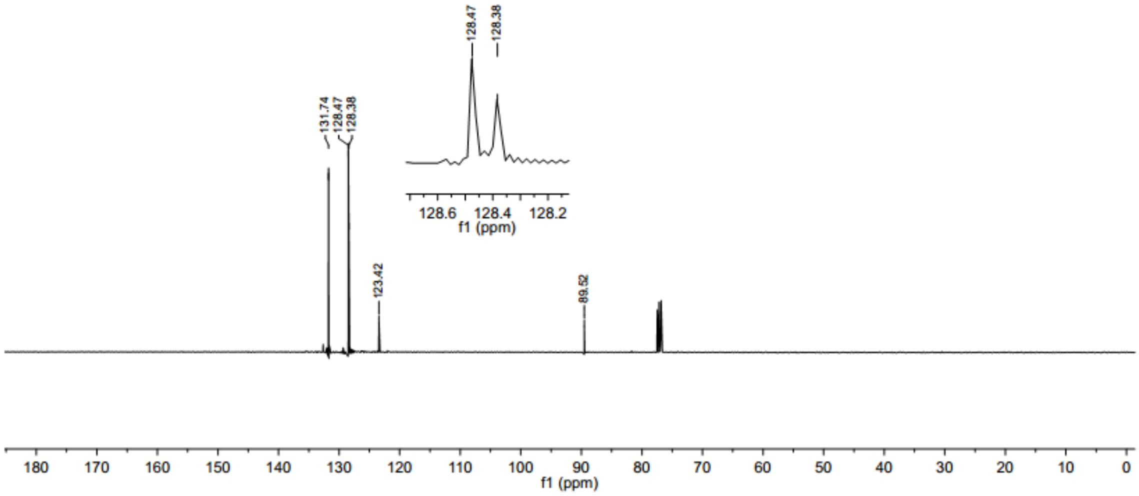

1,4-Dihydronaphthalene (58)

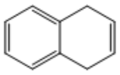

^{1}H NMR (400 MHz, Chloroform-*d*) δ 7.19 – 7.11 (m, 5H), 5.94 (q, *J* = 1.5 Hz, 2H), 3.41 (d, *J* = 1.4 Hz, 5H).

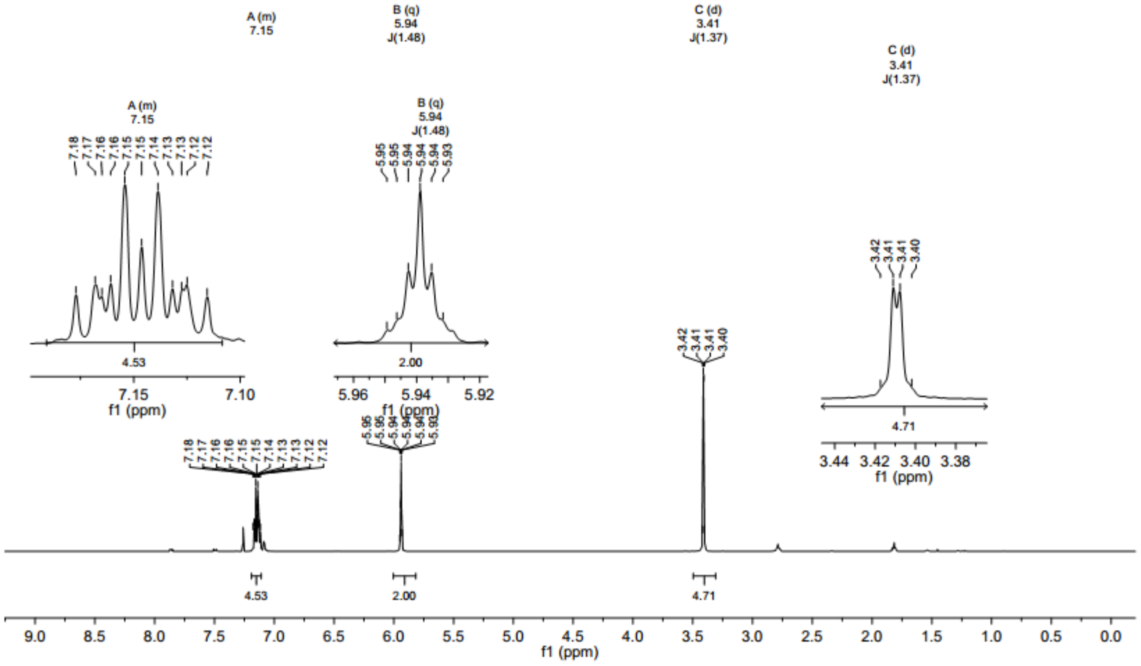

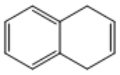

^{13}C NMR (101 MHz, $CDCl_3$) δ 134.35, 128.55, 126.02, 124.91, 29.90.

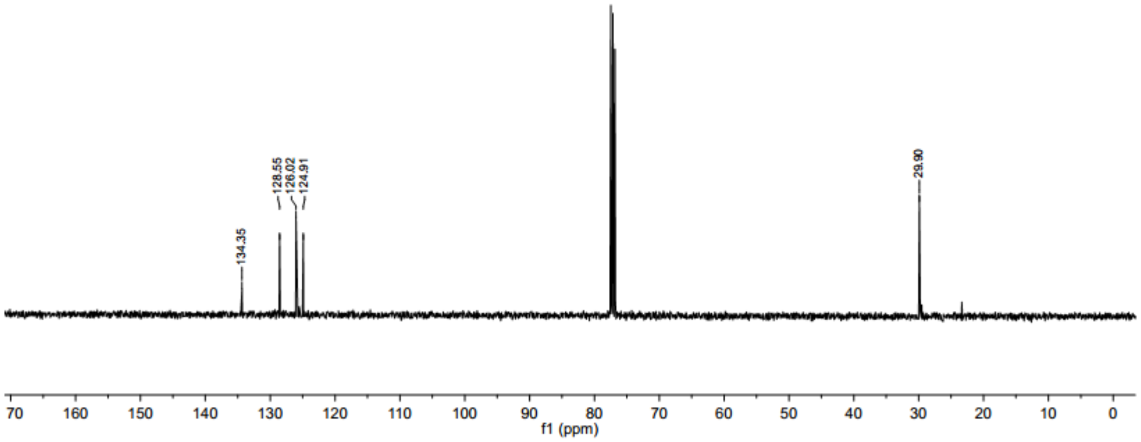

Products

1*H*-indole (24)

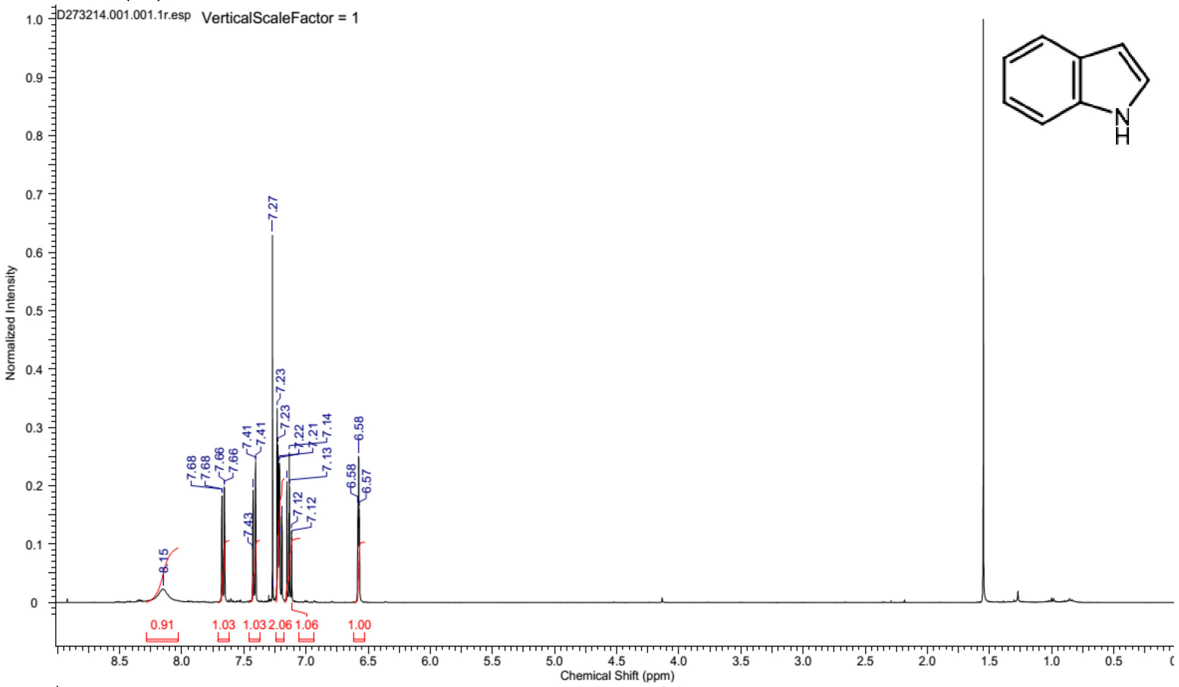

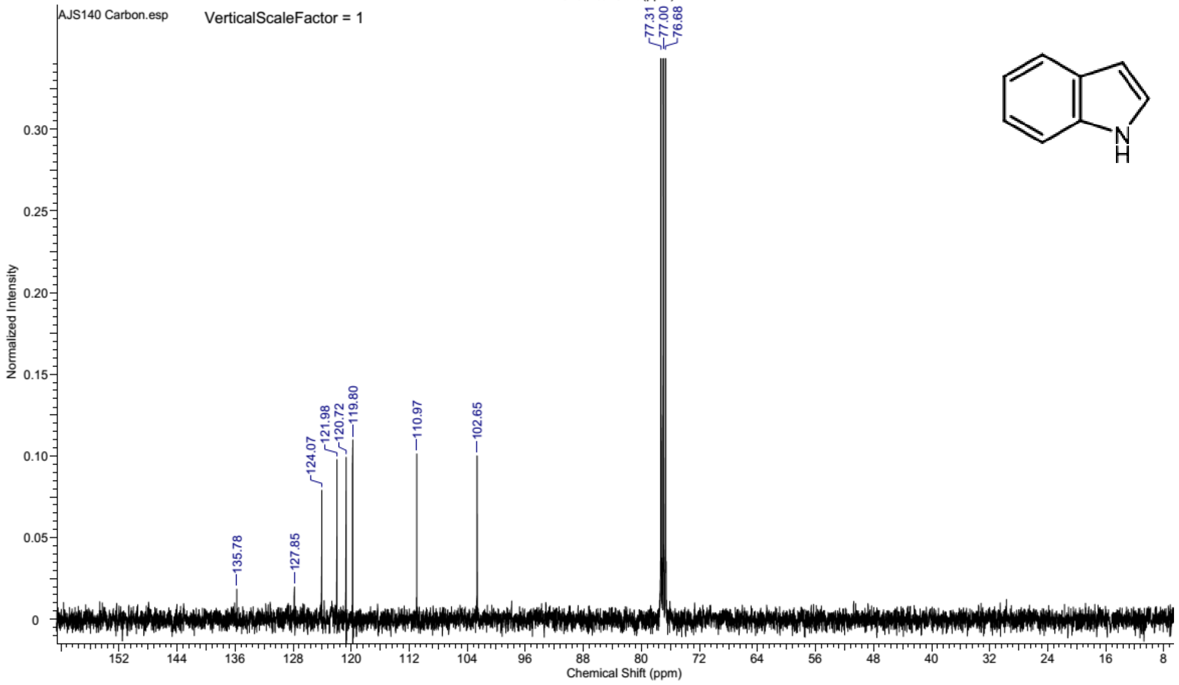

2-Methyl-1*H*-indole (25)

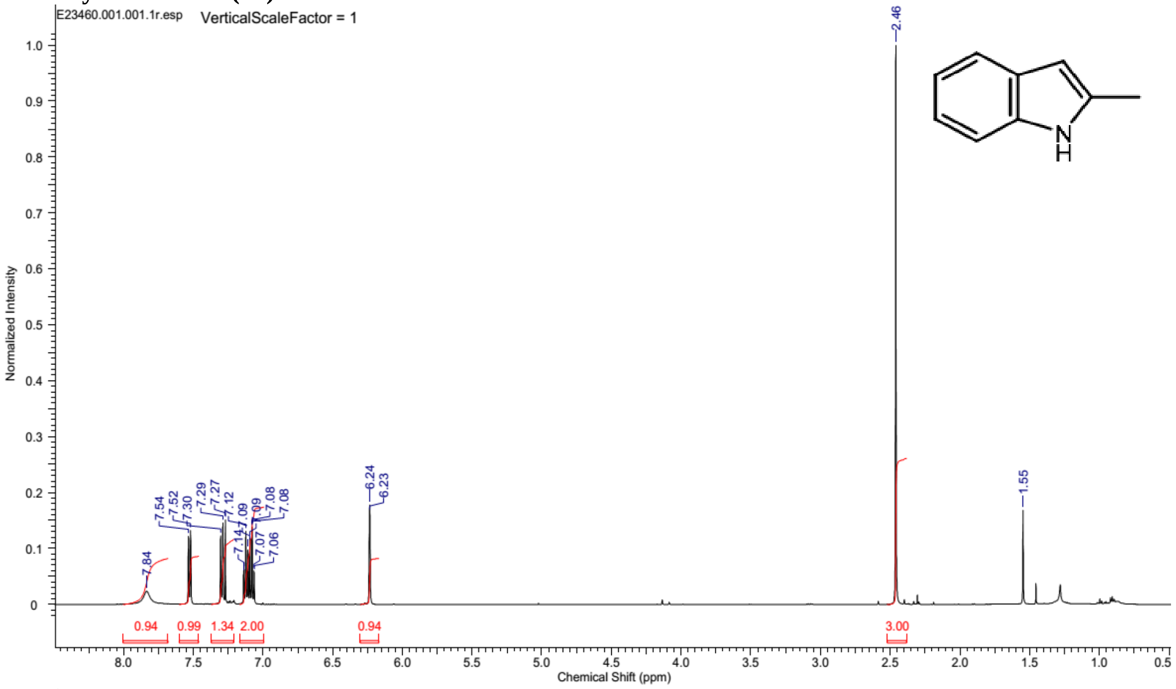

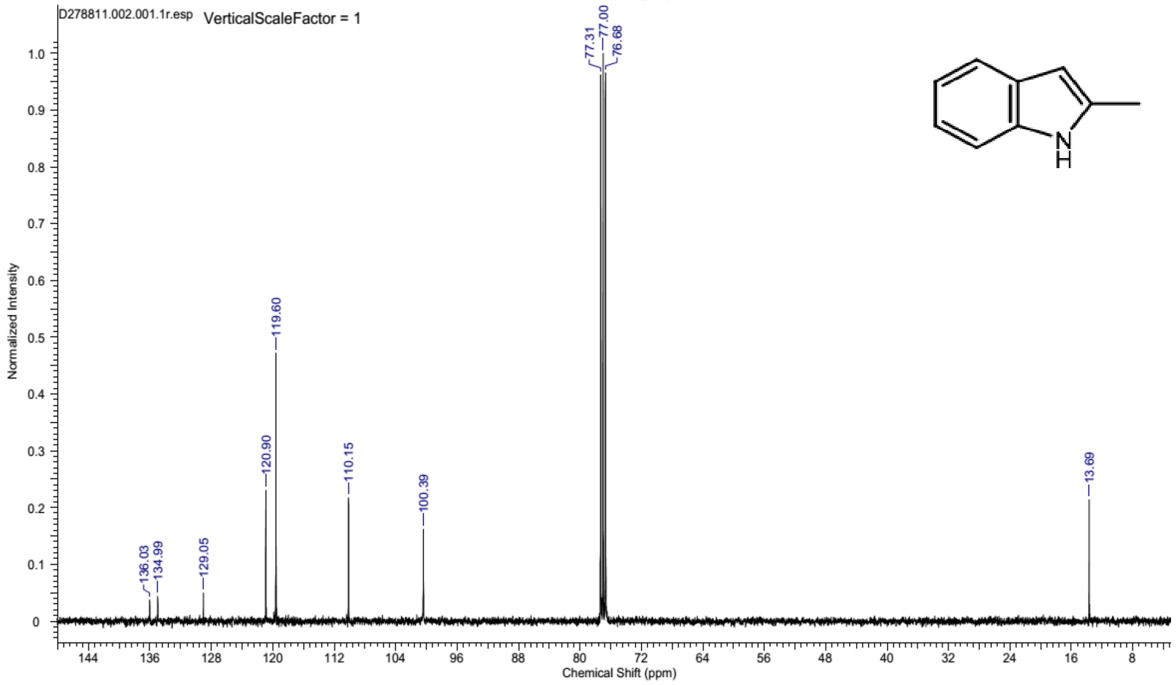

3-Methyl-1*H*-indole (26)

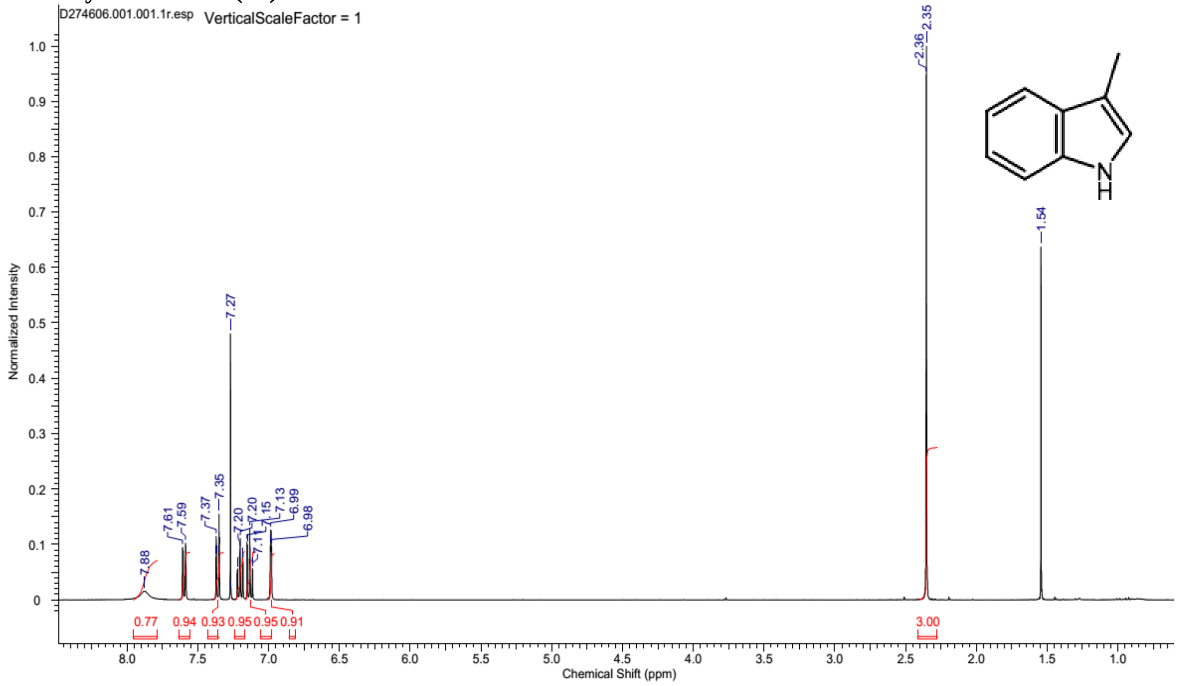

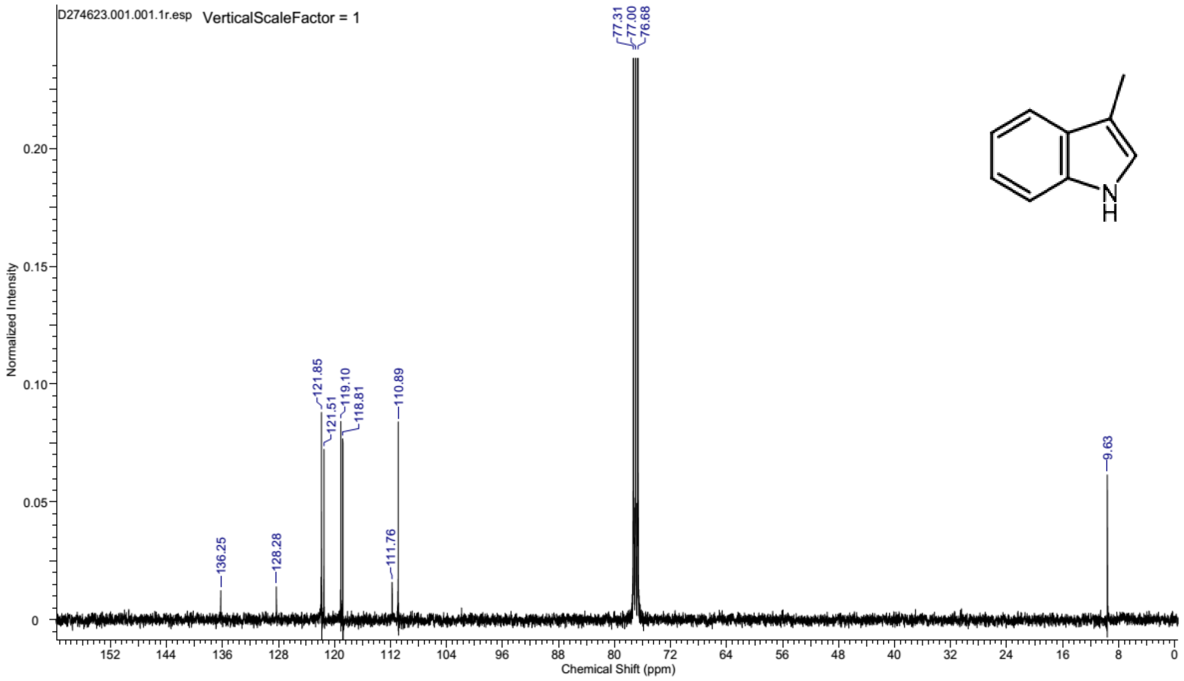

9*H*-carbazole (28)

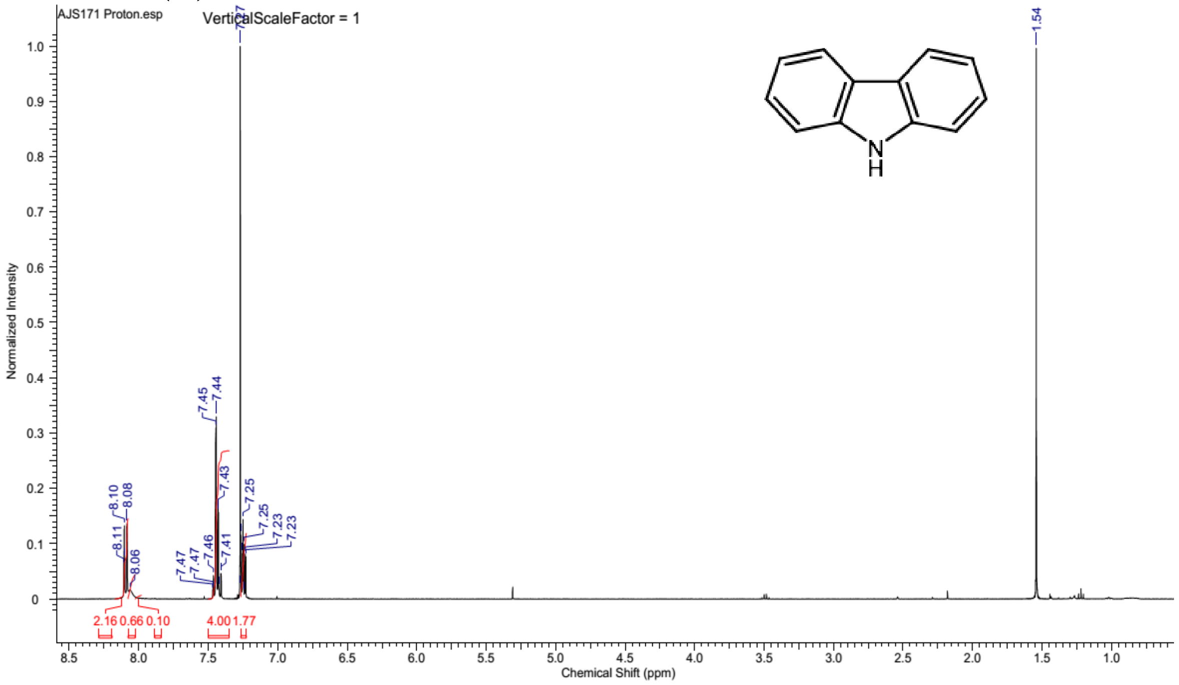

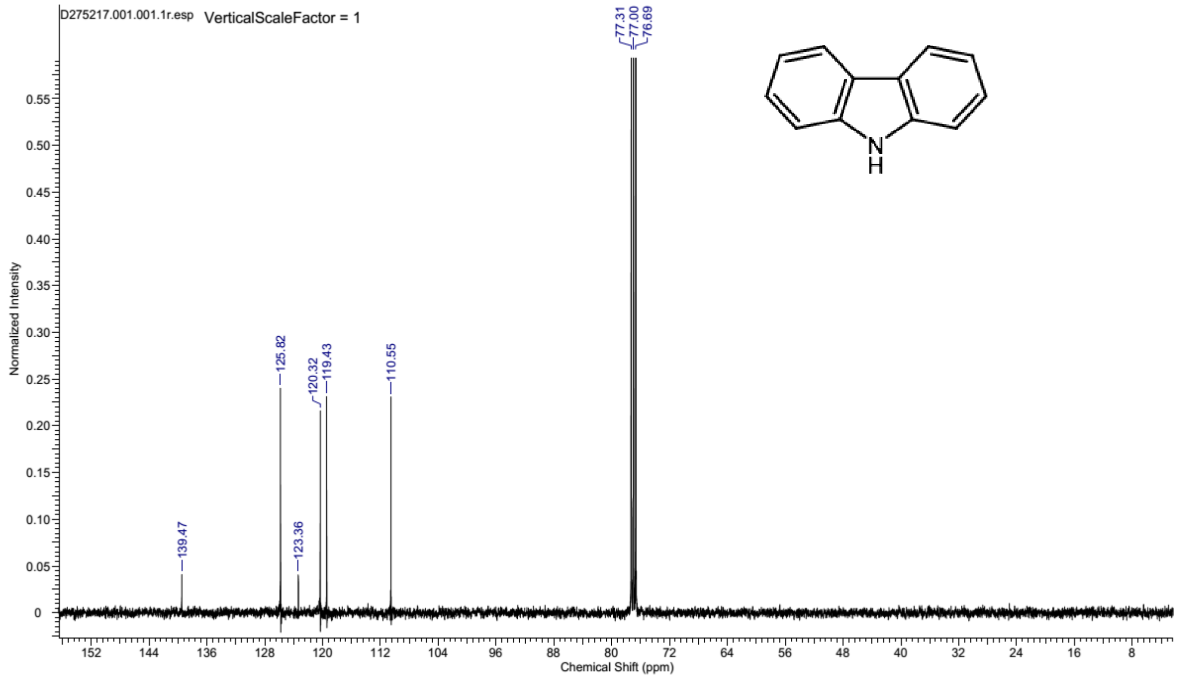

2-(2-Methylbenzyl)-1*H*-indole (29)

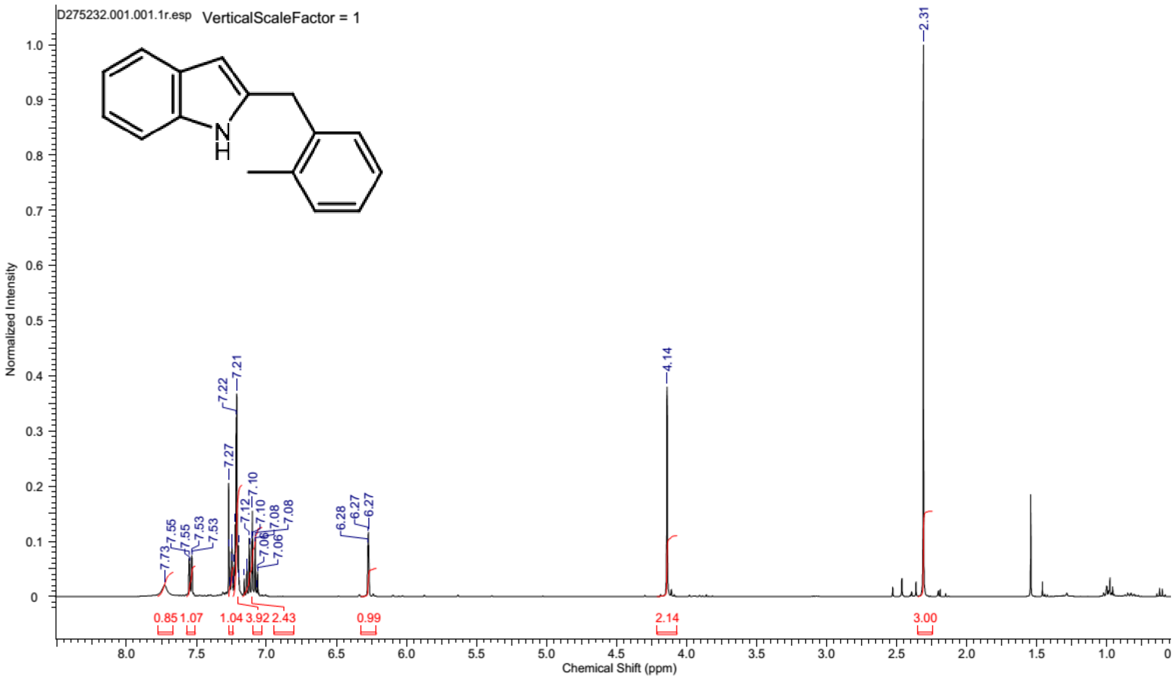

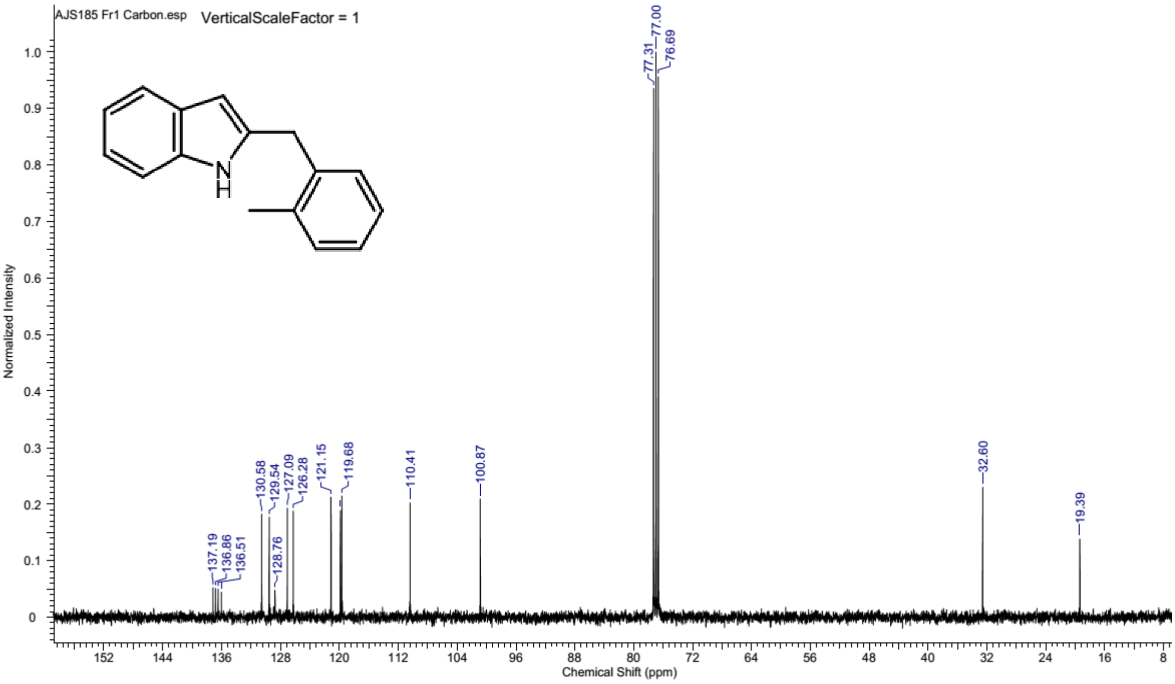

1-Methylnaphthalene (30)

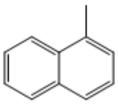

^{1}H NMR (600 MHz, Chloroform-*d*) δ 8.02 (d, *J* = 8.3 Hz, 1H), 7.86 (d, *J* = 7.8 Hz, 1H), 7.72 (d, *J* = 8.1 Hz, 1H), 7.54 (ddd, *J* = 8.5, 6.8, 1.6 Hz, 1H), 7.52 – 7.48 (m, 1H), 7.41 – 7.36 (m, 1H), 7.34 (d, *J* = 7.0 Hz, 1H), 2.72 (s, 3H).

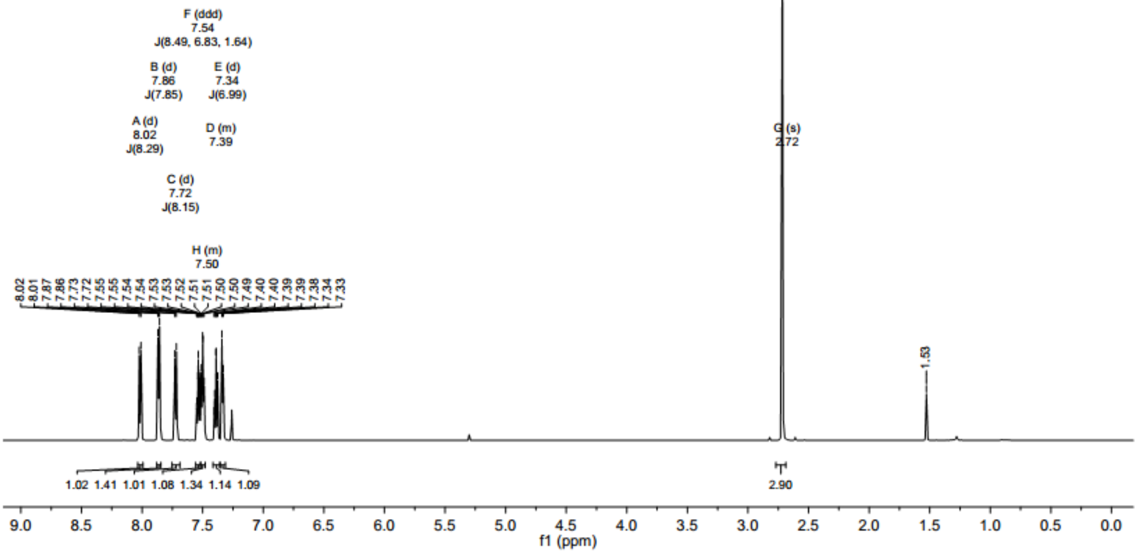

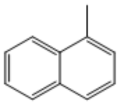

^{13}C NMR (101 MHz, $CDCl_3$) δ 134.39, 133.68, 132.74, 128.65, 126.69, 126.50, 125.84, 125.70, 125.66, 124.24, 19.52.

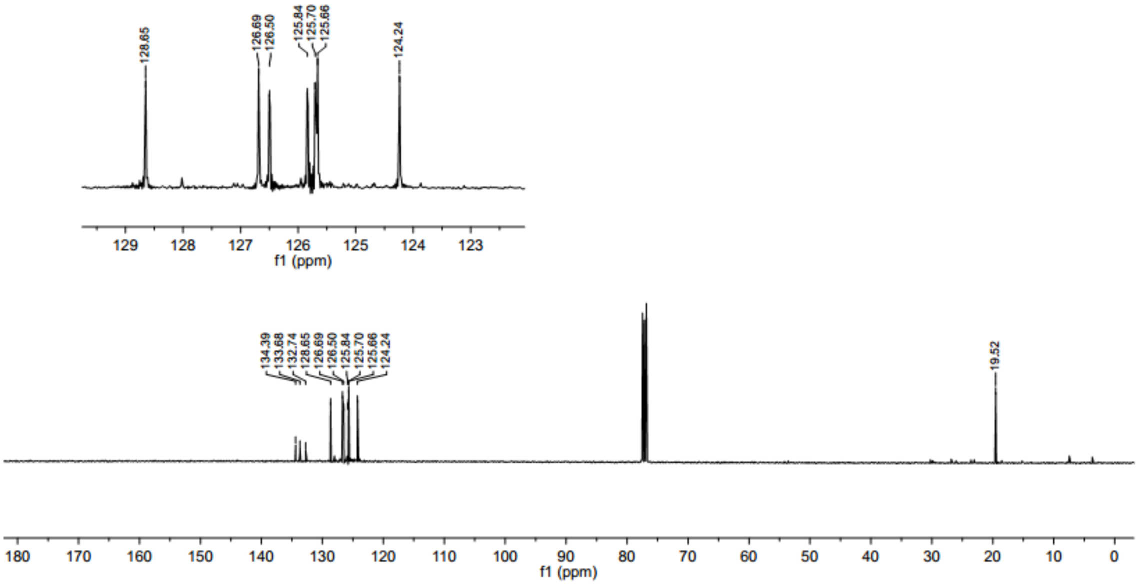

***N*-methyl-*N*-phenylacetamide (42)**

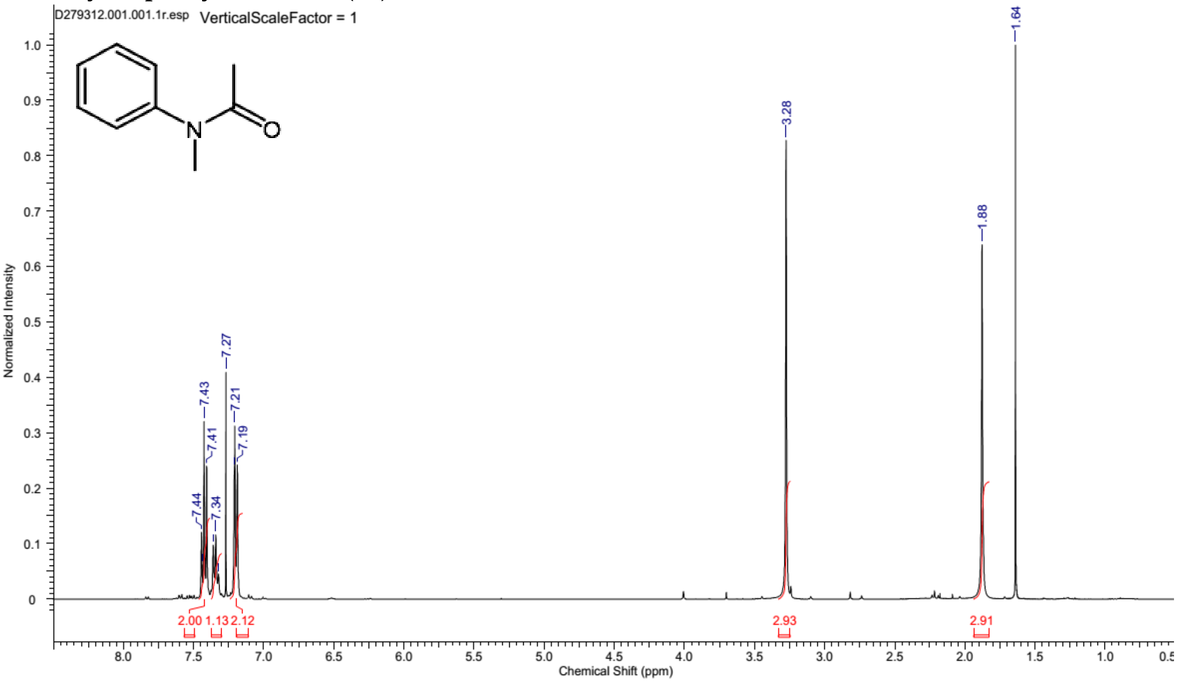

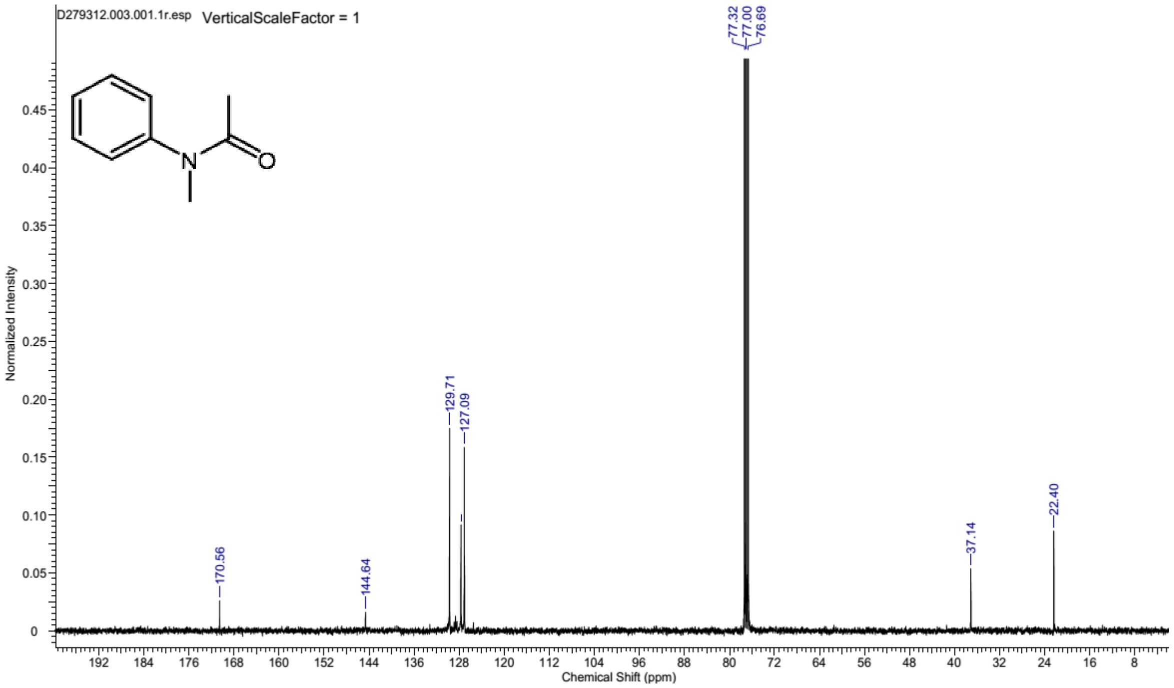

2-Isopropylaniline (44)

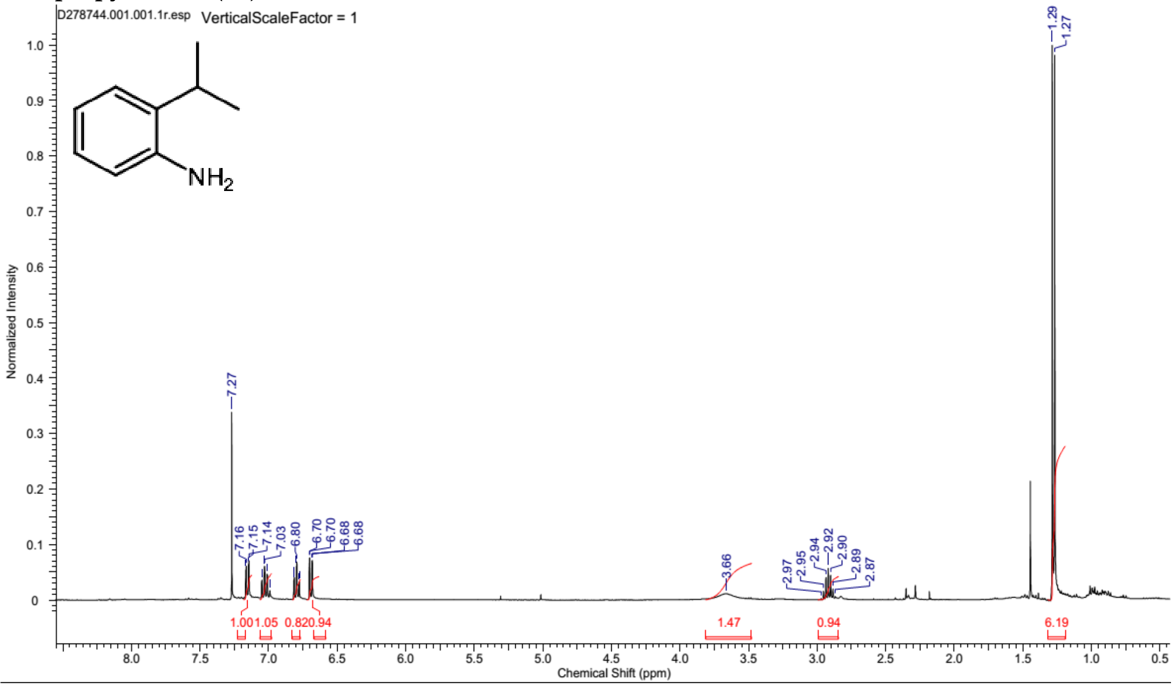

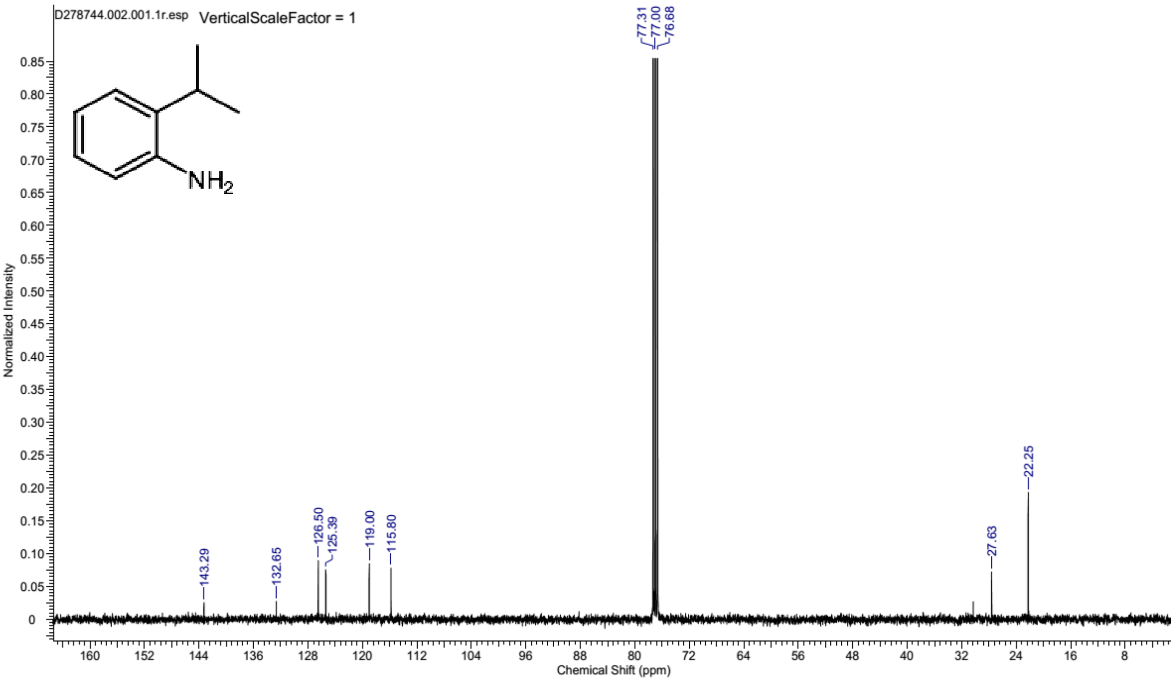

2,3-Dimethyl-1*H*-indole (46)

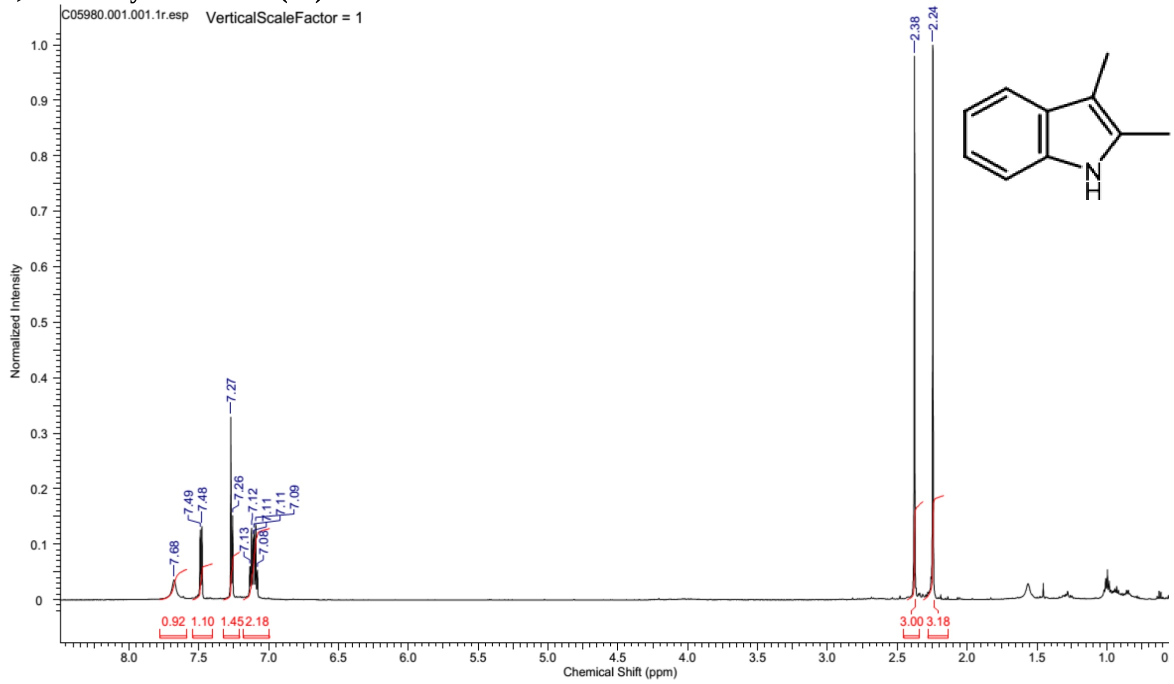

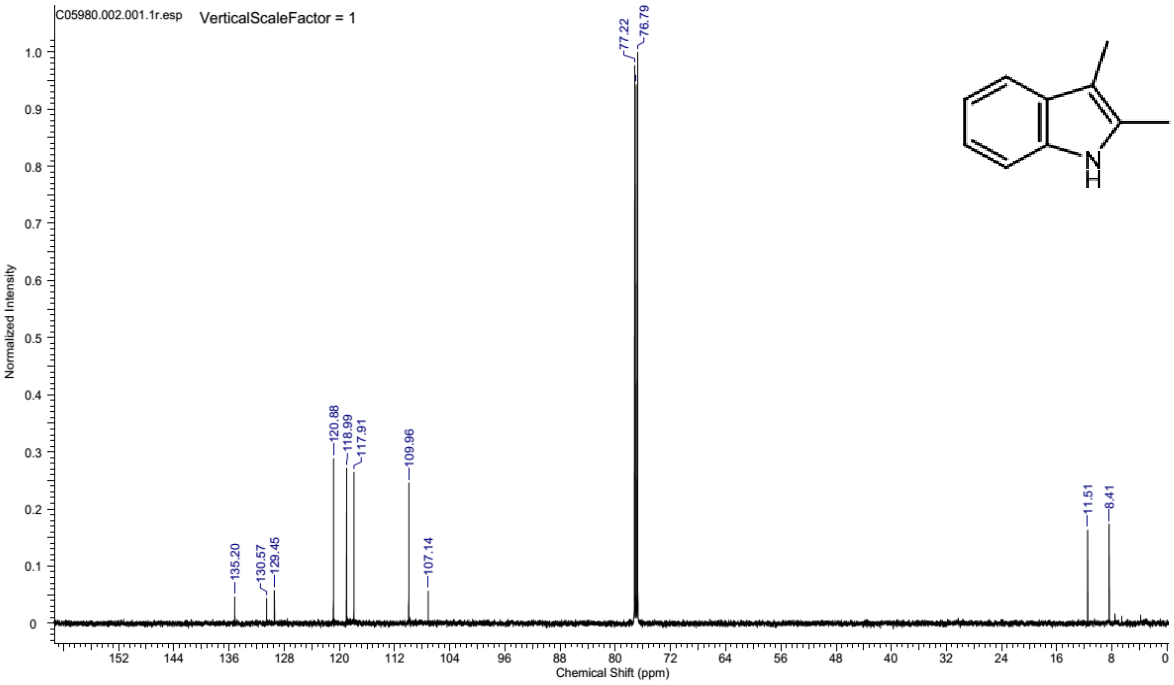

Octan-2-ylbenzene (48)

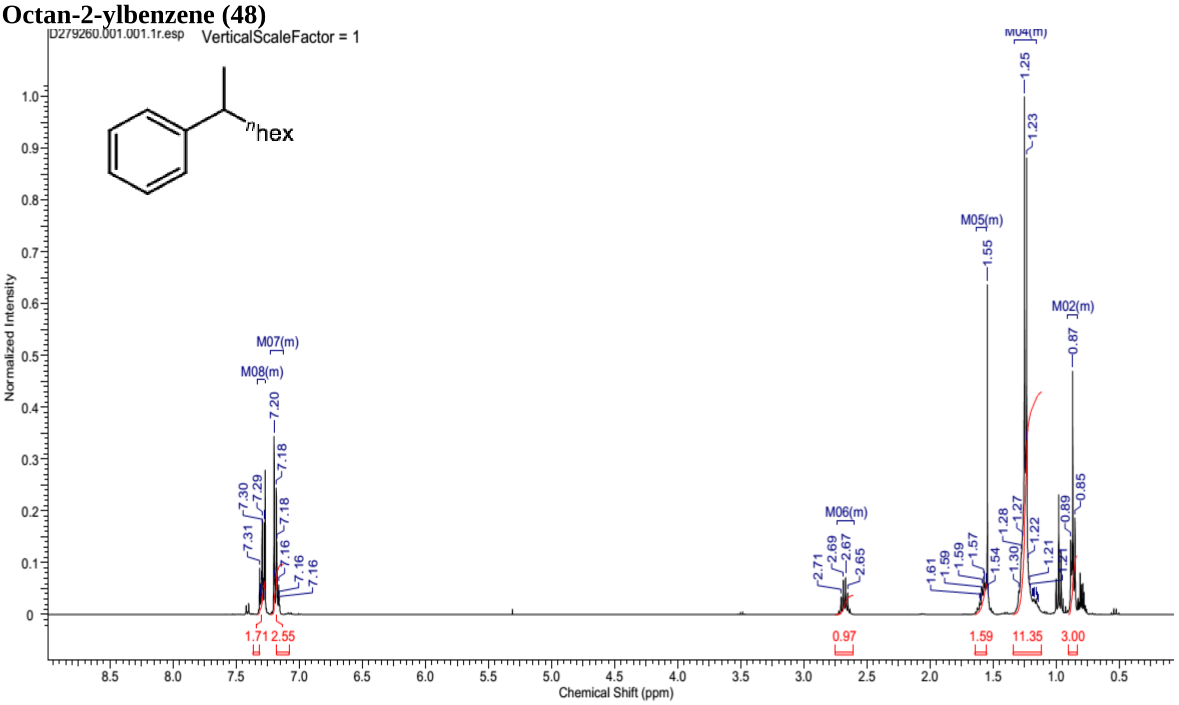

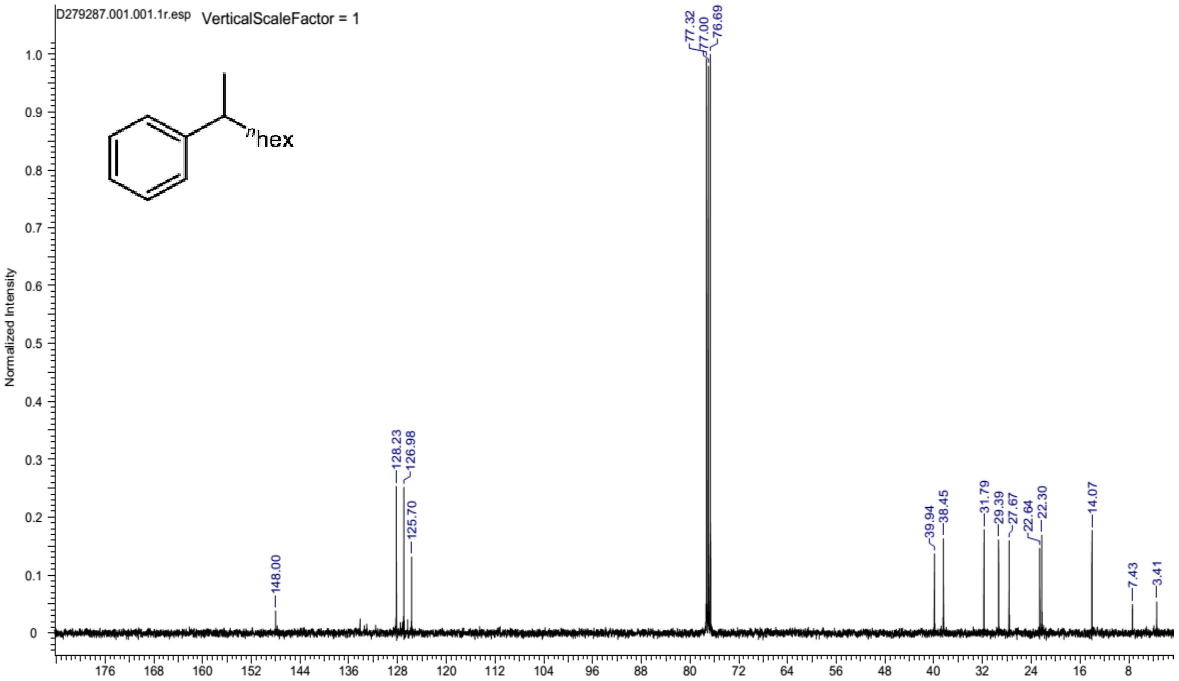

4-Phenylheptane (50)

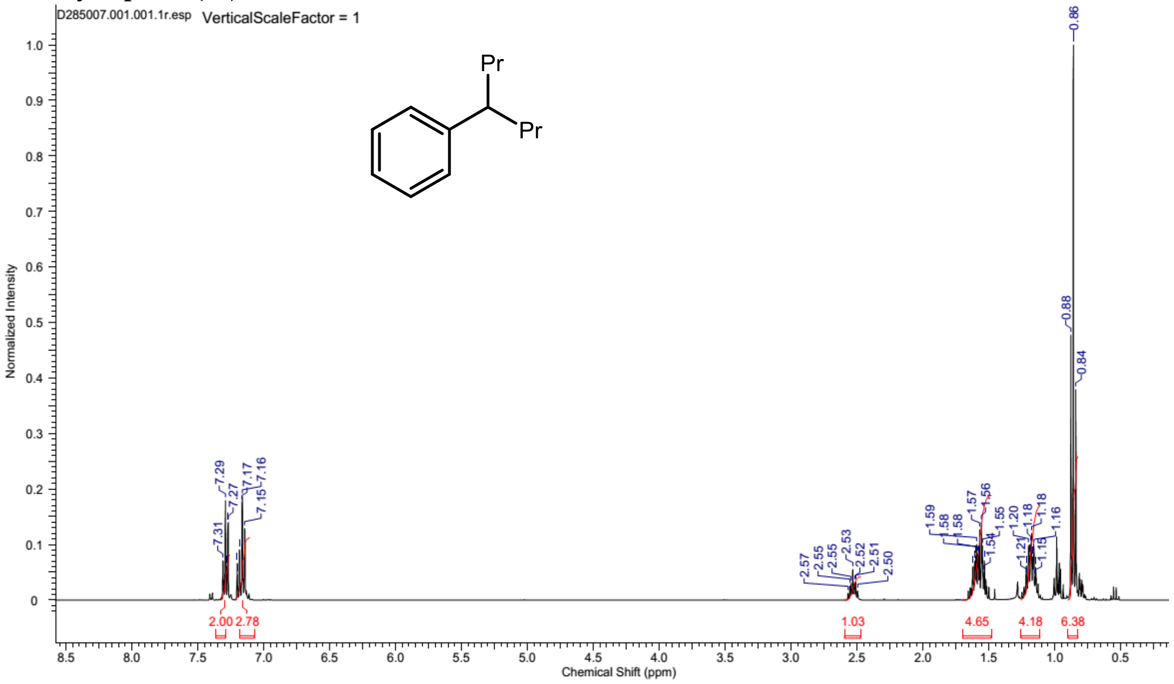

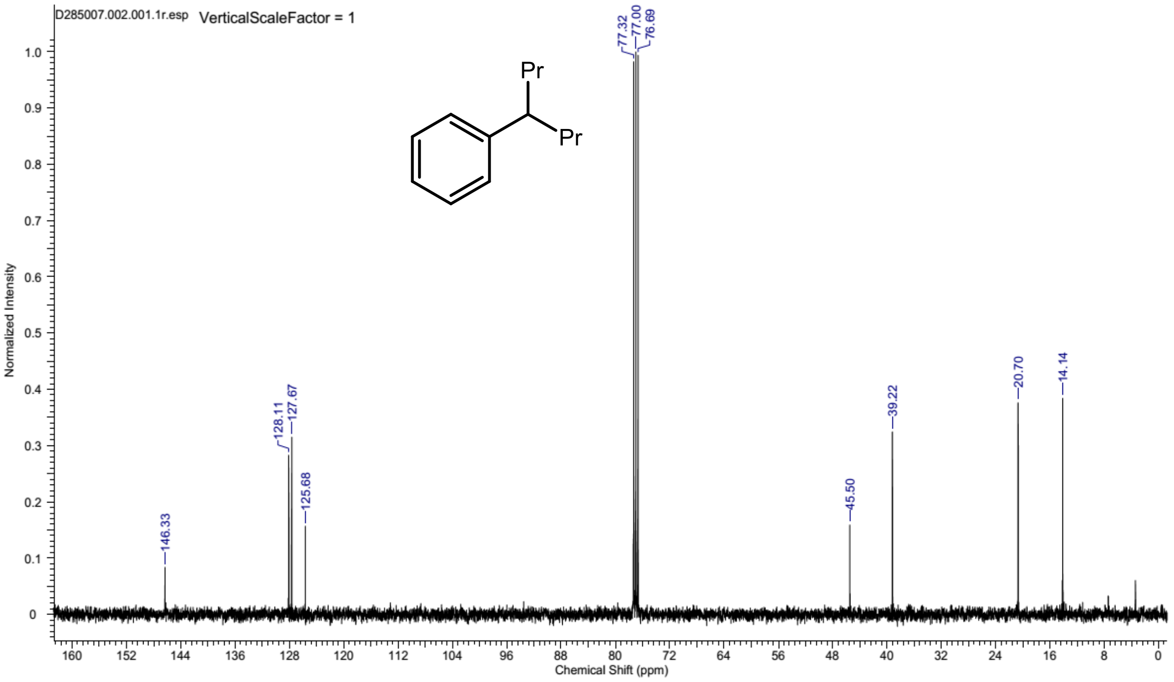

9,10-Dihydroanthracene (56)

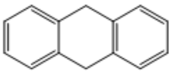

^{1}H NMR (400 MHz, Chloroform-*d*) δ 7.31 (dd, *J* = 5.5, 3.4 Hz, 4H), 7.21 (dd, *J* = 5.6, 3.3 Hz, 4H), 3.96 (s, 4H).

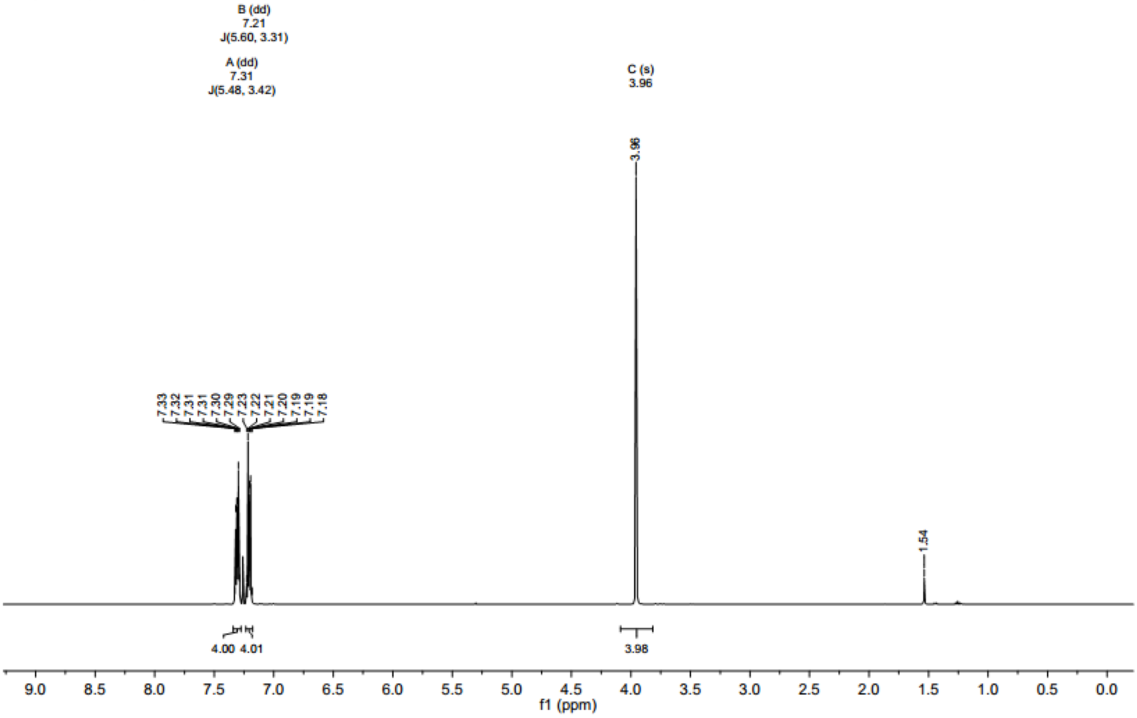

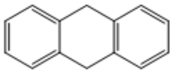

^{13}C NMR (101 MHz, $CDCl_3$) δ 136.82, 127.52, 126.22, 36.31.

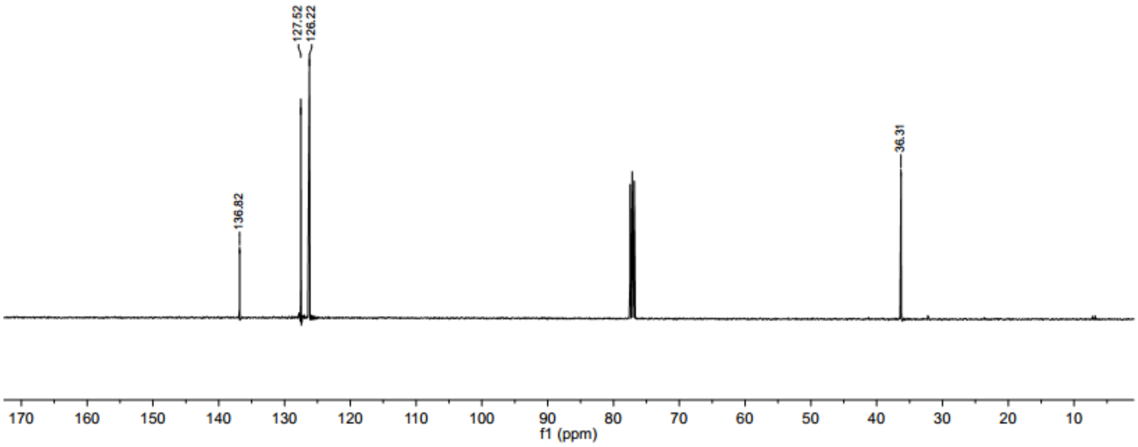

9,10-Dihydrophenanthrene (57)

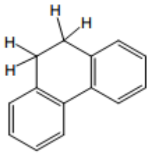

^{1}H NMR (400 MHz, Chloroform-*d*) δ 7.77 (dt, *J* = 7.6, 0.9 Hz, 2H), 7.32 (ddd, *J* = 7.7, 5.4, 3.5 Hz, 2H), 7.28 – 7.21 (m, 4H), 2.89 (s, 3H).

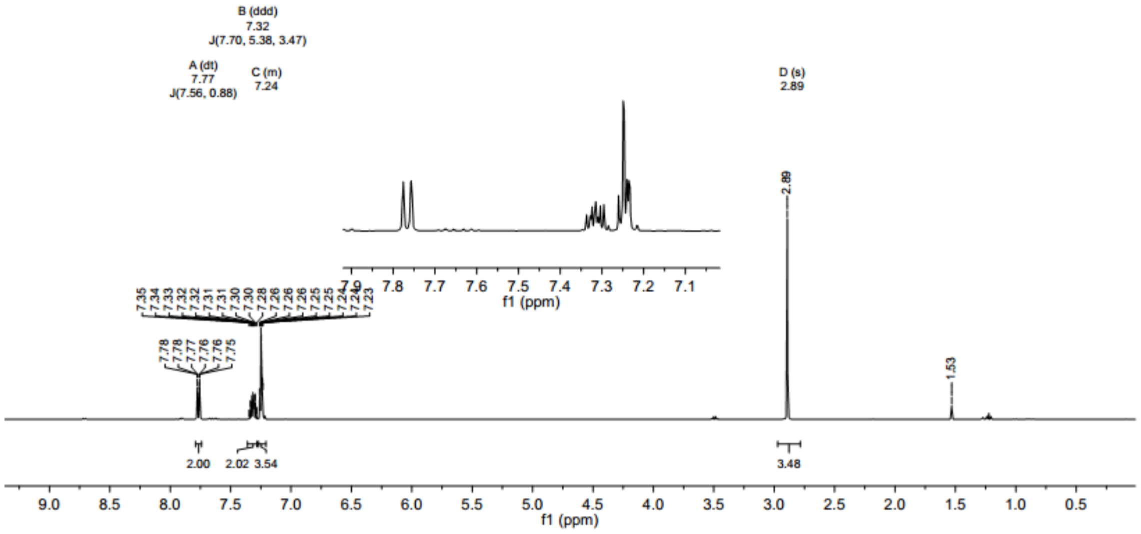

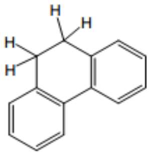

^{13}C NMR (101 MHz, $CDCl_3$) δ 137.53, 134.63, 128.26, 127.51, 127.08, 123.84, 29.20.

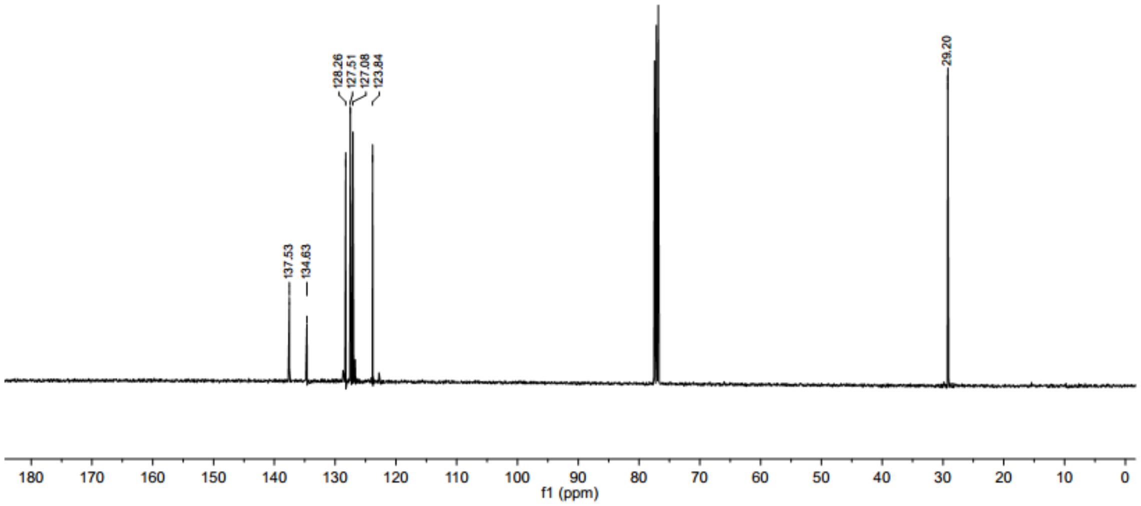

1,2-Diphenylethane (59)

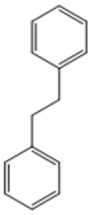

^{1}H NMR (400 MHz, Chloroform-*d*) δ 7.32 – 7.26 (m, 4H), 7.23 – 7.17 (m, 6H), 2.93 (s, 4H).

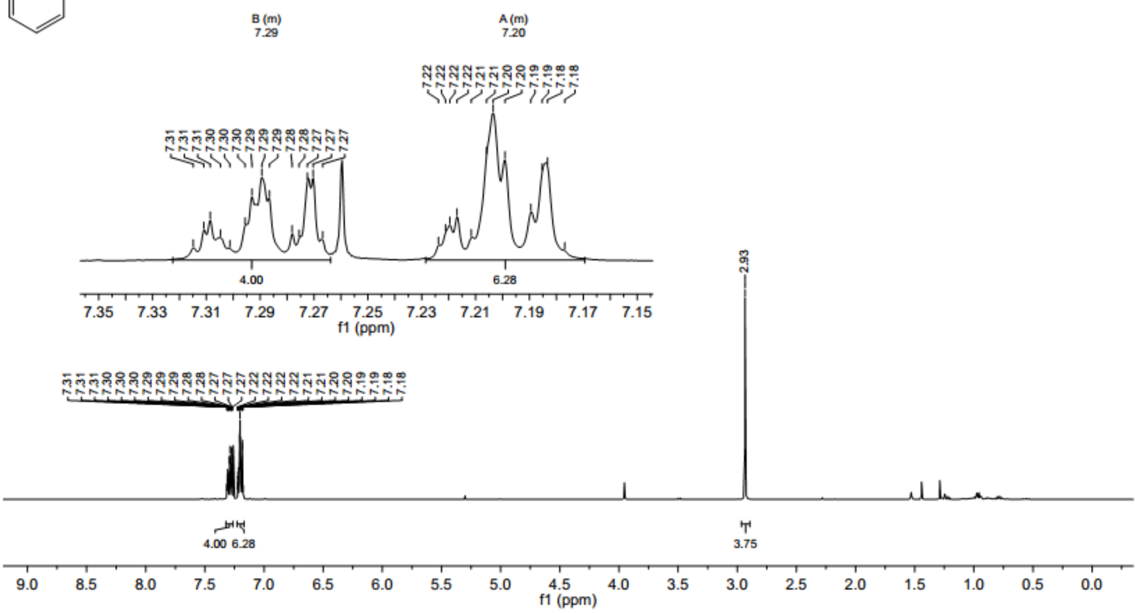

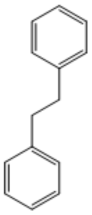

^{13}C NMR (101 MHz, $CDCl_3$) δ 141.94, 128.59, 128.47, 126.06, 38.10.

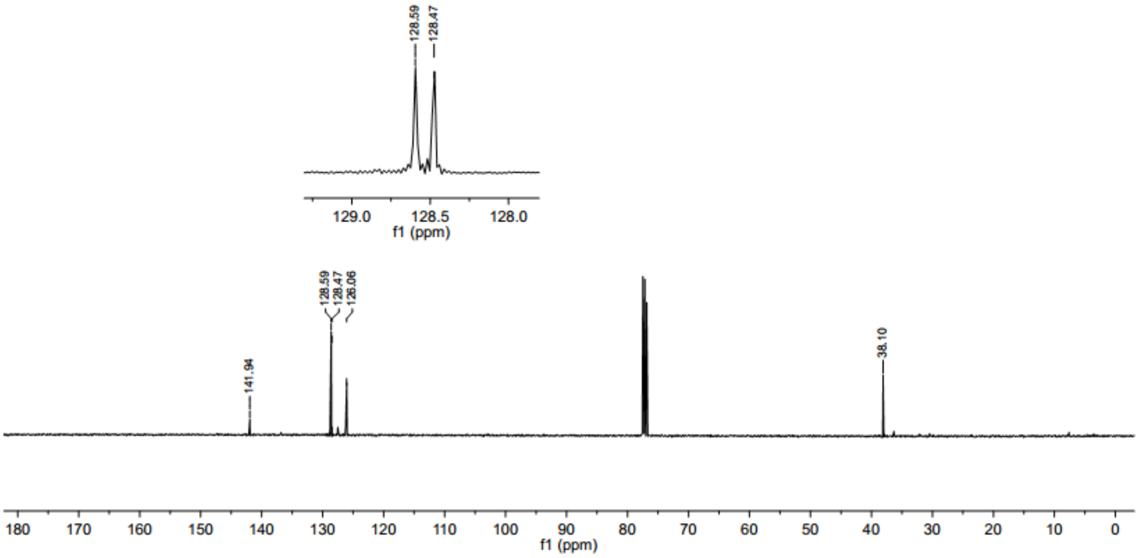

XYZ Coordinates

[Me_3SiO^tBu] $^{\bullet -}$

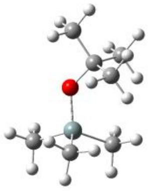

27

-642.1956462

Si	1.24702	-0.00146	0.05218
C	1.67711	1.65539	-0.72056
H	1.19799	1.80820	-1.69511
H	1.40133	2.48777	-0.05877
C	2.32651	-0.29430	1.55511
H	2.08371	-1.25148	2.03141
H	2.19108	0.50608	2.29226
C	1.49046	-1.38427	-1.19698
H	2.54901	-1.41041	-1.48893
H	1.22720	-2.36341	-0.77525
O	-0.30418	-0.00003	0.70233
C	-1.58733	0.00494	0.08259
C	-1.63018	0.98045	-1.09467
C	-1.93092	-1.41112	-0.38483
C	-2.56765	0.45086	1.16688
H	-1.32272	1.98133	-0.77307
H	-0.96267	0.65853	-1.90290
H	-2.64531	1.03906	-1.50184
H	-1.85000	-2.10986	0.45426
H	-2.95274	-1.44894	-0.77788
H	-1.24762	-1.73631	-1.17483
H	-3.59341	0.46147	0.78438
H	-2.51569	-0.23287	2.02009
H	-2.30932	1.45653	1.51275
H	0.91224	-1.22589	-2.11680
H	3.38283	-0.31337	1.26341
H	2.76131	1.70262	-0.88757

[Me_3SiO^tBu] $^{\bullet -}$ (Acetonitrile as Solvent)

27

-642.2194072

Si	1.24337	-0.01029	0.06123
C	1.72095	1.74903	-0.40395
H	1.19833	2.09739	-1.30094
H	1.50238	2.44306	0.41518
C	2.32669	-0.60907	1.46921
H	2.04673	-1.63163	1.76128

H	2.22999	0.04074	2.35099
C	1.45311	-1.11016	-1.44997
H	2.51333	-1.09068	-1.74135
H	1.20305	-2.15330	-1.21811
O	-0.30510	-0.05078	0.70509
C	-1.59130	-0.00414	0.08275
C	-1.63452	1.09029	-0.98440
C	-1.91317	-1.36870	-0.52927
C	-2.57987	0.31113	1.20298
H	-1.36707	2.05810	-0.54731
H	-0.93768	0.87121	-1.80121
H	-2.63985	1.16563	-1.41072
H	-1.80465	-2.15192	0.22991
H	-2.94040	-1.38424	-0.90913
H	-1.23617	-1.59935	-1.35709
H	-3.60451	0.34108	0.81981
H	-2.51662	-0.45815	1.97978
H	-2.34379	1.28050	1.65296
H	0.85648	-0.78139	-2.31039
H	3.38381	-0.63657	1.16793
H	2.79900	1.78959	-0.60363

[Me_3SiO^tBu] $^{\bullet-}$ Optimised Geometry - Single Point as Neutral Singlet

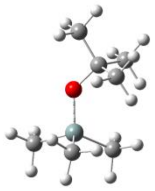

27

-642.1948130

Si	1.24701700	-0.00146500	0.05217900
C	1.67710600	1.65539000	-0.72056400
H	1.19799300	1.80819800	-1.69511100
H	1.40132900	2.48777300	-0.05877300
C	2.32650900	-0.29429700	1.55510800
H	2.08370800	-1.25147700	2.03140600
H	2.19108400	0.50607700	2.29226100
C	1.49045600	-1.38427500	-1.19697600
H	2.54900800	-1.41041500	-1.48893200
H	1.22719800	-2.36341100	-0.77525200
O	-0.30417800	-0.00003000	0.70233300
C	-1.58732700	0.00494300	0.08258500
C	-1.63018400	0.98044800	-1.09466700
C	-1.93091700	-1.41111900	-0.38482900
C	-2.56764700	0.45086100	1.16688300
H	-1.32272300	1.98133200	-0.77307500

H	-0.96267000	0.65852600	-1.90290000
H	-2.64531400	1.03905600	-1.50183600
H	-1.84999900	-2.10986300	0.45426400
H	-2.95273800	-1.44893500	-0.77788300
H	-1.24761800	-1.73631300	-1.17483400
H	-3.59340800	0.46146800	0.78438000
H	-2.51568900	-0.23286600	2.02008700
H	-2.30932100	1.45653300	1.51274500
H	0.91224000	-1.22588600	-2.11679700
H	3.38282700	-0.31337400	1.26341200
H	2.76131000	1.70261700	-0.88757000

Me_3SiO^tBu

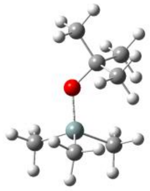

27

-642.1951837

Si	1.23710	-0.00363	0.06301
O	-0.30285	-0.01102	0.71352
C	-1.58713	0.00267	0.08614
C	-1.60160	0.95632	-1.10927
C	-1.94268	-1.41769	-0.35492
C	-2.56668	0.48483	1.15339
H	-1.30215	1.96236	-0.79880
H	-0.91936	0.61568	-1.89614
H	-2.60672	1.00954	-1.53850
H	-1.88625	-2.09774	0.50031
H	-2.95821	-1.45084	-0.76229
H	-1.25235	-1.77138	-1.12625
H	-3.58889	0.50072	0.76311
H	-2.53139	-0.18107	2.02049
H	-2.29872	1.49392	1.48030
C	1.68671	1.68160	-0.63996
H	2.76619	1.72574	-0.82427
H	1.43478	2.47745	0.06873
C	1.46047	-1.30791	-1.27462
H	2.51131	-1.32485	-1.58604
H	0.85627	-1.10371	-2.16459
C	2.34612	-0.38550	1.52103
H	3.39878	-0.39108	1.21929
H	2.10700	-1.36476	1.94705
H	1.17764	1.89270	-1.58479
H	2.22172	0.36608	2.30687

H	1.20252	-2.30575	-0.90552

Me_3SiO^tBu (Acetonitrile as Solvent)

27

-642.1966460

Si	1.23834	-0.00340	0.06138
O	-0.30243	-0.00805	0.71952
C	-1.58737	0.00299	0.08697
C	-1.59693	0.95133	-1.11233
C	-1.94125	-1.41917	-0.34877
C	-2.57111	0.48996	1.14793
H	-1.29885	1.95892	-0.80545
H	-0.91261	0.60711	-1.89558
H	-2.60089	1.00201	-1.54420
H	-1.89323	-2.09588	0.50990
H	-2.95400	-1.45134	-0.76266
H	-1.24651	-1.77739	-1.11384
H	-3.59067	0.50392	0.75119
H	-2.54344	-0.17271	2.01819
H	-2.30671	1.50150	1.47090
C	1.68427	1.67955	-0.64681
H	2.76340	1.72020	-0.83384
H	1.43457	2.47817	0.05965
C	1.45709	-1.31477	-1.26793
H	2.50767	-1.33103	-1.58021
H	0.85024	-1.11407	-2.15678
C	2.34869	-0.37812	1.52122
H	3.39966	-0.38406	1.21370
H	2.11307	-1.35665	1.95139
H	1.17184	1.88536	-1.59093
H	2.22844	0.37792	2.30376
H	1.20038	-2.31054	-0.89236

Me_3SiO^tBu Optimised Geometry - Single Point as Radical Anion

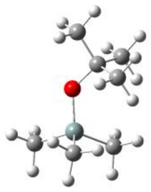

27

-642.1953560

Si	1.23709600	-0.00362500	0.06301300
O	-0.30284700	-0.01102400	0.71351500
C	-1.58713000	0.00267000	0.08613800
C	-1.60160400	0.95631700	-1.10926700
C	-1.94268100	-1.41768500	-0.35492400

C	-2.56668000	0.48482900	1.15339200
H	-1.30215500	1.96235700	-0.79880200
H	-0.91935600	0.61568000	-1.89613800
H	-2.60672000	1.00954400	-1.53849800
H	-1.88625200	-2.09773800	0.50030900
H	-2.95820700	-1.45084200	-0.76229200
H	-1.25235200	-1.77138000	-1.12625100
H	-3.58888900	0.50072100	0.76310900
H	-2.53138700	-0.18107300	2.02048700
H	-2.29872400	1.49391900	1.48029600
C	1.68671400	1.68159600	-0.63995700
H	2.76619100	1.72574100	-0.82426800
H	1.43477800	2.47745400	0.06873400
C	1.46047000	-1.30790500	-1.27461900
H	2.51131400	-1.32484900	-1.58603700
H	0.85627300	-1.10371200	-2.16458700
C	2.34612200	-0.38549900	1.52102700
H	3.39877500	-0.39108200	1.21928700
H	2.10699800	-1.36475600	1.94705000
H	1.17763800	1.89270200	-1.58479500
H	2.22171800	0.36607900	2.30687400
H	1.20251700	-2.30575400	-0.90551600

Me_3SiH

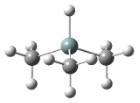

14

-409.7572729

Si	0.00007200	-0.00029400	0.38340600
H	-0.00014400	-0.00032900	1.87436600
C	-0.35645700	1.74518800	-0.22455700
H	-0.36026400	1.77139800	-1.31936300
H	-1.33278600	2.09429300	0.12550900
H	0.40360500	2.44913700	0.12820300
C	-1.33309900	-1.18101800	-0.22470900
H	-1.35029300	-1.20187300	-1.31960800
H	-1.15105900	-2.20000300	0.13021800
H	-2.32335300	-0.87128200	0.12316600
C	1.68951900	-0.56376200	-0.22473900
H	2.48001800	0.10513500	0.12917100
H	1.91793500	-1.57544400	0.12427000
H	1.71555700	-0.56935800	-1.31958600

O^{t}Bu Anion

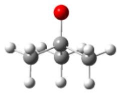

14

-233.0233916

C	-0.00012	0.00003	0.13526
C	0.52734	1.34520	-0.43205
H	-0.10559	2.16181	-0.06397
H	1.54590	1.51511	-0.06233
H	0.54198	1.37997	-1.53160
C	-1.42824	-0.21596	-0.43337
H	-1.46500	-0.22193	-1.53294
H	-1.81966	-1.17183	-0.06435
H	-2.08492	0.58165	-0.06500
C	0.90169	-1.12873	-0.43262
H	1.92497	-0.98967	-0.06297
H	0.53908	-2.09618	-0.06439
H	0.92595	-1.15762	-1.53219
O	-0.00084	-0.00056	1.49455

Me_3SiH + O^{t}Bu Anion Transition State

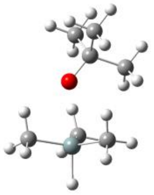

28

-642.7889046

Si	-1.94862	0.00563	-0.12335
C	-2.17298	0.04789	1.74808
H	-2.79813	-0.79360	2.06932
H	-1.19711	-0.00465	2.23276
C	-1.38241	1.61488	-0.94035
H	-0.41776	1.93051	-0.54278
H	-2.13417	2.39447	-0.76766
C	-1.44345	-1.66196	-0.85840
H	-1.28546	-1.56699	-1.93977
H	-0.51929	-2.01081	-0.39687
H	-3.41557	0.02861	-0.57779
O	0.48060	-0.03413	0.70813
C	1.71418	-0.00483	0.11494

C	1.63847	-0.19025	-1.42308
C	2.42762	1.34309	0.38884
C	2.61393	-1.13468	0.67299
H	1.19579	-1.16396	-1.66037
H	1.00009	0.58587	-1.86004
H	2.62771	-0.13491	-1.89913
H	2.50790	1.49191	1.47227
H	3.43437	1.39586	-0.05009
H	1.82750	2.16493	-0.02033
H	3.61963	-1.14680	0.22805
H	2.71143	-1.01517	1.75871
H	2.13468	-2.10247	0.48145
H	-2.24712	-2.39141	-0.70259
H	-1.29564	1.47671	-2.02522
H	-2.68517	0.97118	2.04386

$[(Me)_3(H)SiO^tBu]^-$

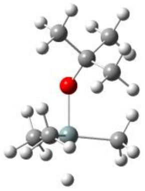

28

-642.8007830

Si	-1.47268	0.00004	-0.10047
C	-2.24565	-0.00010	1.65152
H	-2.89312	-0.88024	1.75420
H	-1.50963	0.00014	2.46042
C	-1.22208	1.70088	-0.97324
H	-0.29188	2.22156	-0.72180
H	-2.06966	2.34107	-0.69824
C	-1.22227	-1.70071	-0.97347
H	-1.26967	-1.57006	-2.06314
H	-0.29229	-2.22166	-0.72176
H	-2.98469	0.00020	-0.73768
O	0.21440	-0.00012	0.67872
C	1.50422	-0.00003	0.13714
C	1.56383	-0.00008	-1.40419
C	2.24266	1.24964	0.64764
C	2.24283	-1.24957	0.64772
H	1.07797	-0.88678	-1.81962
H	1.07735	0.88624	-1.81971
H	2.61008	0.00026	-1.73350
H	2.23048	1.25775	1.74274
H	3.28511	1.27827	0.30565
H	1.73432	2.15332	0.29475

H	3.28523	-1.27817	0.30560
H	2.23078	-1.25757	1.74281
H	1.73449	-2.15332	0.29499
H	-2.07012	-2.34073	-0.69890
H	-1.26995	1.57044	-2.06293
H	-2.89376	0.87956	1.75419

[(Me)$_3$(H)SiO^{t}Bu]$^-$ Optimised Geometry - Single Point as Radical

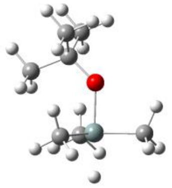

28

-642.6370549

Si	-1.47267700	0.00004200	-0.10047200
C	-2.24564700	-0.00010300	1.65152300
H	-2.89312100	-0.88024000	1.75420400
H	-1.50962800	0.00014300	2.46042100
C	-1.22208200	1.70087700	-0.97324300
H	-0.29187900	2.22155800	-0.72180000
H	-2.06966200	2.34106700	-0.69823500
C	-1.22226900	-1.70071000	-0.97346500
H	-1.26966500	-1.57006000	-2.06314300
H	-0.29228800	-2.22166000	-0.72175900
H	-2.98468600	0.00019600	-0.73767900
O	0.21439500	-0.00012200	0.67872200
C	1.50422100	-0.00003200	0.13714500
C	1.56383000	-0.00007900	-1.40419200
C	2.24266300	1.24963900	0.64764300
C	2.24282800	-1.24957400	0.64771600
H	1.07797100	-0.88677600	-1.81962000
H	1.07734800	0.88623900	-1.81970900
H	2.61008000	0.00026500	-1.73350200
H	2.23047900	1.25775500	1.74273600
H	3.28510500	1.27826700	0.30564600
H	1.73431800	2.15331700	0.29475400
H	3.28523000	-1.27817200	0.30559500
H	2.23077700	-1.25756500	1.74281100
H	1.73448600	-2.15331800	0.29498900
H	-2.07011600	-2.34073000	-0.69889700
H	-1.26994900	1.57044300	-2.06292600
H	-2.89375700	0.87956000	1.75418900

[(Me)$_3$(H)SiO^{t}Bu]$^\bullet$

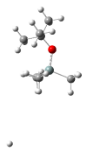

28

-642.6934994

Si	-1.13662	-0.26542	-0.05019
C	-1.76425	1.13758	-1.13383
H	-1.38178	2.11329	-0.82047
H	-1.47978	0.97826	-2.17921
C	-2.03366	-1.84600	-0.49733
H	-1.69669	-2.67786	0.12870
H	-1.84776	-2.11115	-1.54289
C	-1.44676	0.16135	1.75487
H	-2.52465	0.27352	1.91918
H	-1.08662	-0.63212	2.41759
H	-6.74591	3.56340	0.38525
O	0.47040	-0.61255	-0.35569
C	1.66598	0.09979	-0.02960
C	1.47471	1.60498	-0.22018
C	2.05526	-0.21452	1.41534
C	2.73423	-0.41863	-0.98951
H	1.14064	1.82008	-1.24012
H	0.73330	2.00253	0.48193
H	2.41779	2.13110	-0.04387
H	2.14279	-1.29681	1.54970
H	3.01578	0.24844	1.66331
H	1.30251	0.16311	2.11352
H	3.69916	0.05785	-0.79111
H	2.84573	-1.50065	-0.87378
H	2.44390	-0.20860	-2.02322
H	-0.96928	1.10035	2.05324
H	-3.11430	-1.73163	-0.36221
H	-2.85849	1.17589	-1.08582

$[(Me)_3(H)SiO^tBu]^\bullet$ Optimised Geometry - Single Point as Anion

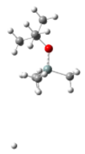

28

-642.7866389			
Si	-1.13661700	-0.26542400	-0.05019200
C	-1.76424600	1.13758100	-1.13383100
H	-1.38177800	2.11328600	-0.82046700
H	-1.47978400	0.97826100	-2.17920800
C	-2.03366300	-1.84600000	-0.49733200
H	-1.69669200	-2.67786200	0.12870200
H	-1.84776400	-2.11115200	-1.54289200
C	-1.44675800	0.16134900	1.75486800
H	-2.52464900	0.27352300	1.91918100
H	-1.08662400	-0.63212300	2.41759000
H	-6.74590700	3.56340100	0.38524600
O	0.47040300	-0.61255000	-0.35569200
C	1.66597900	0.09979300	-0.02960200
C	1.47471000	1.60498200	-0.22018100
C	2.05526000	-0.21452000	1.41533900
C	2.73423000	-0.41862800	-0.98950900
H	1.14064500	1.82008500	-1.24012500
H	0.73330000	2.00252600	0.48193300
H	2.41778800	2.13110200	-0.04386800
H	2.14279300	-1.29681000	1.54969800
H	3.01577800	0.24844400	1.66330800
H	1.30251400	0.16311300	2.11351700
H	3.69916100	0.05785200	-0.79110700
H	2.84573200	-1.50064800	-0.87377600
H	2.44390300	-0.20860300	-2.02322400
H	-0.96928200	1.10035000	2.05324100
H	-3.11430300	-1.73163400	-0.36220800
H	-2.85849300	1.17588500	-1.08582300

Me_3SiH + O^{t}Bu Deprotonation Transition State

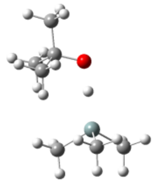

28			
-642.7657237			
Si	1.66708	-0.00864	-0.17398
C	1.85492	-0.97215	1.48108
H	2.87357	-0.88195	1.88293
H	1.15900	-0.59219	2.23970
H	1.63857	-2.03902	1.34601
C	2.31934	1.74137	0.28459
H	3.31628	1.68998	0.74381
H	2.38786	2.38243	-0.60228

H	1.64632	2.23711	0.99491
C	3.08440	-0.73010	-1.25476
H	2.89880	-1.78285	-1.49822
H	3.17573	-0.18554	-2.20191
H	4.05114	-0.66888	-0.73587
H	0.05443	-0.01044	-0.78339
O	-1.24633	0.04922	-1.20382
C	-2.12366	-0.00284	-0.13229
C	-1.81652	1.11343	0.89140
C	-3.56427	0.18483	-0.63798
C	-2.02774	-1.36520	0.58829
H	-0.81009	0.98009	1.30491
H	-1.84851	2.08693	0.38865
H	-2.53373	1.12230	1.72334
H	-3.79999	-0.59820	-1.36756
H	-4.30055	0.14088	0.17683
H	-3.65323	1.15487	-1.14014
H	-2.72649	-1.44179	1.43267
H	-2.24756	-2.16904	-0.12370
H	-1.00875	-1.51349	0.96366

HO^{t}Bu

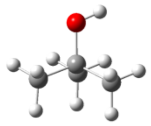

15
-233.5788851

C	-0.00580	-0.00002	0.01546
C	0.67402	1.25900	-0.52194
H	0.19636	2.15110	-0.10656
H	1.73479	1.27282	-0.24606
H	0.60870	1.30188	-1.61360
C	-1.49013	-0.00180	-0.32737
H	-1.63320	-0.00228	-1.41167
H	-1.97189	-0.89027	0.09113
H	-1.97382	0.88587	0.09059
C	0.67726	-1.25700	-0.52252
H	1.73795	-1.26842	-0.24620
H	0.20155	-2.15054	-0.10795
H	0.61251	-1.29929	-1.61423
O	0.04771	-0.00030	1.44751
H	0.97326	0.00044	1.72264

$[SiMe_3]^-$

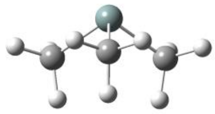

13

-409.1849033

Si	0.00024	-0.00016	-0.68327
C	-0.68852	1.55089	0.28821
H	-0.62595	1.39897	1.37871
H	-0.12288	2.45966	0.04318
H	-1.73924	1.74658	0.03623
C	1.68748	-0.17936	0.28869
H	1.52520	-0.16203	1.37933
H	2.19353	-1.12137	0.03921
H	2.38027	0.63511	0.03898
C	-0.99942	-1.37128	0.28836
H	-2.06821	-1.34006	0.03829
H	-0.63931	-2.37879	0.04143
H	-0.90401	-1.23729	1.37879

$[SiMe_3]^-$ Optimised Geometry - Single Point as Radical

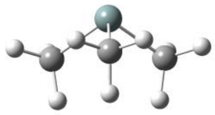

13

-409.0890178

Si	0.00023800	-0.00016300	-0.68326800
C	-0.68851600	1.55088900	0.28821500
H	-0.62595100	1.39896900	1.37871400
H	-0.12288200	2.45965600	0.04317600
H	-1.73924200	1.74658400	0.03622500
C	1.68747600	-0.17936100	0.28869200
H	1.52520500	-0.16202600	1.37932700
H	2.19352600	-1.12137400	0.03920900
H	2.38027200	0.63511300	0.03898300
C	-0.99941500	-1.37127800	0.28836100
H	-2.06820600	-1.34005700	0.03829300
H	-0.63931100	-2.37879400	0.04142700
H	-0.90401100	-1.23729100	1.37879400

[SiMe$_3$]$^{\bullet}$

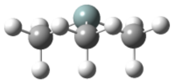

13
-409.1077933

Si	-0.00007	-0.00038	-0.43654
C	0.01239	1.78529	0.18119
H	-0.86868	2.33249	-0.16638
H	0.01046	1.80137	1.27850
C	1.54056	-0.90315	0.18134
H	1.55348	-0.91255	1.27864
H	2.45450	-0.41078	-0.16318
C	-1.55283	-0.88182	0.18139
H	-1.58591	-1.91910	-0.16407
H	-1.56691	-0.88896	1.27869
H	1.56046	-1.94015	-0.16595
H	0.90269	2.31927	-0.16321
H	-2.45988	-0.37818	-0.16500

[SiMe$_3$]$^{\bullet}$ Optimised Geometry - Single Point as Anion

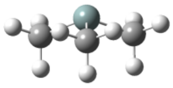

13
-409.1653795

Si	-0.00006800	-0.00037900	-0.43654000
C	0.01238900	1.78529100	0.18119300
H	-0.86867600	2.33248500	-0.16638200
H	0.01045700	1.80136500	1.27849800
C	1.54056000	-0.90314900	0.18134100
H	1.55348400	-0.91254800	1.27863700
H	2.45450200	-0.41078300	-0.16318500
C	-1.55282600	-0.88182300	0.18138900
H	-1.58590900	-1.91909800	-0.16407200
H	-1.56690800	-0.88896200	1.27868900
H	1.56046000	-1.94015400	-0.16595300
H	0.90268600	2.31926700	-0.16321400
H	-2.45988400	-0.37818000	-0.16500000

N-Benzylcarbazole

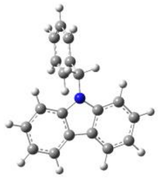

Optimised Geometry

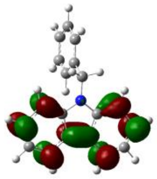

LUMO

35

-787.5458075

C	-2.69143	-3.02704	0.48011
C	-1.49075	-3.41362	-0.14050
C	-0.58935	-2.47408	-0.62845
C	-0.92366	-1.12471	-0.48392
C	-2.11989	-0.71716	0.15246
C	-3.01022	-1.68268	0.63221
H	-3.37092	-3.78787	0.84972
H	-1.25790	-4.46945	-0.23781
H	0.34184	-2.78355	-1.09269
H	-3.93409	-1.38527	1.12009
C	-0.91113	1.12641	-0.48450
C	-0.56164	2.47181	-0.63006
C	-1.45255	3.42176	-0.14306
C	-2.65748	3.04901	0.47778
C	-2.99125	1.70842	0.63102
C	-2.11179	0.73266	0.15207
H	0.37309	2.77022	-1.09444
H	-1.20797	4.47486	-0.24117
H	-3.32853	3.81768	0.84660
H	-3.91831	1.42180	1.11926
N	-0.21772	-0.00313	-0.88681
C	1.09287	-0.01007	-1.48979
H	1.17028	0.86287	-2.14697
H	1.16419	-0.88967	-2.13867
C	2.23770	-0.00928	-0.49026
C	3.55198	-0.00683	-0.96815
C	2.01294	-0.01148	0.88617
C	4.62636	-0.00683	-0.08308
H	3.73322	-0.00481	-2.04088
C	3.08986	-0.01136	1.77473
H	0.99521	-0.01314	1.26692
C	4.39702	-0.00908	1.29425
H	5.64212	-0.00488	-0.46635
H	2.90267	-0.01298	2.84403
H	5.23355	-0.00893	1.98582

***N*-Benzylcarbazole Radical Anion**

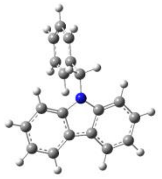

Optimised Geometry

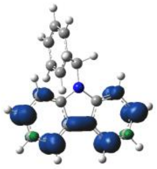

Spin Density

35

-787.5731107

C	-2.60752	-3.05283	0.51588
C	-1.44516	-3.44848	-0.15539
C	-0.56184	-2.46901	-0.69600
C	-0.90351	-1.13841	-0.53490
C	-2.07746	-0.70758	0.16455
C	-2.94772	-1.70840	0.68971
H	-3.26712	-3.81853	0.92012
H	-1.20988	-4.50274	-0.26416
H	0.35099	-2.76205	-1.20756
H	-3.85192	-1.42919	1.22316
C	-0.90325	1.13839	-0.53496
C	-0.56130	2.46890	-0.69619
C	-1.44442	3.44860	-0.15568
C	-2.60685	3.05326	0.51563
C	-2.94733	1.70891	0.68960
C	-2.07727	0.70786	0.16453
H	0.35160	2.76171	-1.20777
H	-1.20892	4.50280	-0.26456
H	-3.26629	3.81913	0.91981
H	-3.85153	1.42993	1.22317
N	-0.23082	-0.00010	-0.97833
C	1.08888	-0.00025	-1.53979
H	1.19171	0.87835	-2.18823
H	1.19159	-0.87897	-2.18807
C	2.20489	-0.00021	-0.50556
C	3.53806	-0.00045	-0.92771
C	1.92670	0.00012	0.86209
C	4.57798	-0.00038	-0.00092
H	3.76125	-0.00071	-1.99299
C	2.96717	0.00018	1.79211
H	0.89248	0.00032	1.19624
C	4.29403	-0.00007	1.36607
H	5.60862	-0.00058	-0.34397
H	2.73664	0.00043	2.85326
H	5.10213	-0.00002	2.09146

***N*-Benzylindole**

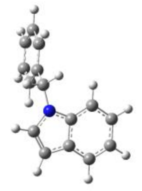

Optimised Geometry

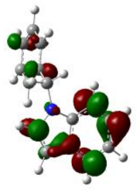

LUMO

29

-633.9479499

C	2.48693200	0.45478500	0.37991400
C	1.36960300	0.07643300	-0.40699300
C	1.19608300	-1.22633800	-0.89268400
C	2.18163900	-2.15335900	-0.58955700
C	3.31058100	-1.79737000	0.18108000
C	3.47054900	-0.50884000	0.66690700
C	2.30937000	1.84252800	0.70767200
C	1.13318400	2.23603100	0.12557300
H	0.32221900	-1.50083000	-1.47655500
H	2.08278700	-3.17277300	-0.94910700
H	4.06204100	-2.55064900	0.39638800
H	4.33988600	-0.24557000	1.26253600
H	2.96914900	2.46622100	1.29324900
H	0.63933100	3.19868800	0.13309200
N	0.56517800	1.18330100	-0.55927900
C	-0.72932300	1.18331800	-1.20745200
H	-0.62919000	0.74125100	-2.20506100
H	-1.02354500	2.22791300	-1.34905400
C	-1.79693600	0.44268800	-0.42121100
C	-1.66954400	0.22157300	0.95142200
C	-2.94441700	-0.00556500	-1.08086000
C	-2.68015100	-0.43325100	1.65613100
H	-0.77429500	0.55481600	1.47088600
C	-3.95677900	-0.65597100	-0.37728400
H	-3.04517000	0.15356200	-2.15205800
C	-3.82682800	-0.87172900	0.99511800
H	-2.56825600	-0.60261500	2.72266800
H	-4.84310600	-0.99962600	-0.90184900
H	-4.61187700	-1.38308700	1.54316900

_N_-Benzylindole Optimised Geometry - Single Point as Radical Anion

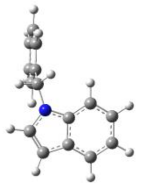

29

-633.9497529

C	2.48693200	0.45478500	0.37991400
C	1.36960300	0.07643300	-0.40699300
C	1.19608300	-1.22633800	-0.89268400
C	2.18163900	-2.15335900	-0.58955700
C	3.31058100	-1.79737000	0.18108000
C	3.47054900	-0.50884000	0.66690700
C	2.30937000	1.84252800	0.70767200
C	1.13318400	2.23603100	0.12557300
H	0.32221900	-1.50083000	-1.47655500
H	2.08278700	-3.17277300	-0.94910700
H	4.06204100	-2.55064900	0.39638800
H	4.33988600	-0.24557000	1.26253600
H	2.96914900	2.46622100	1.29324900
H	0.63933100	3.19868800	0.13309200
N	0.56517800	1.18330100	-0.55927900
C	-0.72932300	1.18331800	-1.20745200
H	-0.62919000	0.74125100	-2.20506100
H	-1.02354500	2.22791300	-1.34905400
C	-1.79693600	0.44268800	-0.42121100
C	-1.66954400	0.22157300	0.95142200
C	-2.94441700	-0.00556500	-1.08086000
C	-2.68015100	-0.43325100	1.65613100
H	-0.77429500	0.55481600	1.47088600
C	-3.95677900	-0.65597100	-0.37728400
H	-3.04517000	0.15356200	-2.15205800
C	-3.82682800	-0.87172900	0.99511800
H	-2.56825600	-0.60261500	2.72266800
H	-4.84310600	-0.99962600	-0.90184900
H	-4.61187700	-1.38308700	1.54316900

***N*-Benzylindole Radical Anion**

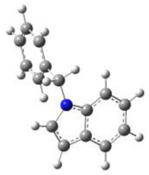

Optimised Geometry

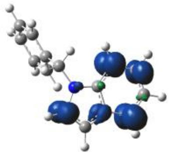

Spin Density

29

-633.9555543

C	2.40541	0.56614	0.44235
C	1.41051	0.09352	-0.45897
C	1.39887	-1.20368	-1.00432
C	2.47554	-2.06750	-0.60688
C	3.47077	-1.62057	0.25828
C	3.48507	-0.31973	0.79975
C	2.05376	1.89894	0.78114
C	0.88192	2.23236	0.10673
H	0.59630	-1.54910	-1.64860
H	2.51456	-3.08351	-0.99174
H	4.27195	-2.30817	0.52821
H	4.26941	-0.00047	1.47960
H	2.60617	2.56083	1.43690
H	0.29551	3.13910	0.07519
N	0.51789	1.12474	-0.66476
C	-0.74162	0.96951	-1.33309
H	-0.59125	0.37978	-2.24697
H	-1.08760	1.96163	-1.64787
C	-1.81378	0.31289	-0.47673
C	-1.60044	0.06381	0.88073
C	-3.04357	-0.02818	-1.04655
C	-2.60517	-0.52132	1.65289
H	-0.63861	0.31575	1.32041
C	-4.04898	-0.60889	-0.27590
H	-3.21286	0.16066	-2.10506
C	-3.83088	-0.85793	1.08018
H	-2.42502	-0.71870	2.70556
H	-4.99861	-0.87177	-0.73333
H	-4.60957	-1.31538	1.68340

N-Benzylindole Radical Anion Optimised Geometry - Single Point as Neutral

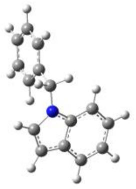

29

-633.9418920

C	2.40541300	0.56614200	0.44235500
C	1.41050600	0.09351900	-0.45896900
C	1.39886700	-1.20367900	-1.00432100
C	2.47553700	-2.06750400	-0.60688000
C	3.47077100	-1.62057300	0.25827600
C	3.48506800	-0.31973300	0.79974700
C	2.05375800	1.89894200	0.78113500
C	0.88191900	2.23235800	0.10673200
H	0.59630500	-1.54909900	-1.64859900
H	2.51456300	-3.08351200	-0.99173600
H	4.27195300	-2.30816900	0.52820800
H	4.26940700	-0.00046500	1.47960400
H	2.60616700	2.56082600	1.43690200
H	0.29551200	3.13909500	0.07518600
N	0.51789200	1.12473800	-0.66475600
C	-0.74161700	0.96951100	-1.33309500
H	-0.59124600	0.37977800	-2.24696700
H	-1.08760000	1.96163400	-1.64786600
C	-1.81378100	0.31288800	-0.47673100
C	-1.60044100	0.06380900	0.88073300
C	-3.04357100	-0.02817700	-1.04655400
C	-2.60517000	-0.52131700	1.65289200
H	-0.63861200	0.31574600	1.32041100
C	-4.04898300	-0.60888700	-0.27589900
H	-3.21285900	0.16065600	-2.10505900
C	-3.83088000	-0.85793200	1.08017600
H	-2.42501700	-0.71870200	2.70556400
H	-4.99861000	-0.87177500	-0.73332600
H	-4.60957000	-1.31537900	1.68340000

Indole Anion

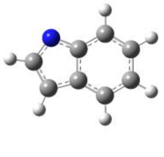

15

-363.1655337

C	-0.29612	0.74301	-0.00002
C	-0.30634	-0.69958	-0.00003
C	0.90897	-1.41169	-0.00000
C	2.10743	-0.71203	0.00004
C	2.12157	0.70418	0.00006
C	0.93686	1.42750	0.00003
C	-1.66881	1.11795	-0.00020
C	-2.36947	-0.09213	0.00033
H	0.89570	-2.49996	-0.00005
H	3.04981	-1.25454	0.00002
H	3.07443	1.22824	0.00004
H	0.96338	2.51622	0.00000
H	-2.08531	2.11782	-0.00032
H	-3.45137	-0.20436	0.00051
N	-1.57874	-1.19524	-0.00020

Toluene

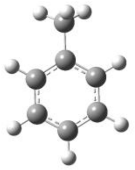

15

-271.4529487

C	-1.90214900	-0.00169200	0.00794000
C	-1.19713000	-1.20581800	0.00200000
C	0.19706300	-1.20072200	-0.00852600
C	0.91265100	0.00228700	-0.01096500
C	0.19415600	1.20250100	-0.00847300
C	-1.20102200	1.20365100	0.00198100
H	-2.98766200	-0.00339800	0.01323900
H	-1.73347100	-2.15013200	0.00188000
H	0.74027800	-2.14275000	-0.01701300
H	0.73437200	2.14610200	-0.01701100
H	-1.73972600	2.14660800	0.00186200
C	2.42085300	0.00091400	0.00872600
H	2.79493200	-0.10499800	1.03268700
H	2.82256700	-0.83124100	-0.57548500
H	2.82217600	0.93309300	-0.39625400

***N*-Benzylindole + [(Me)$_3$(H)SiO^{t}Bu]$^-$ Transition State**

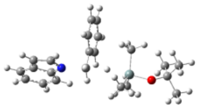

57

-1276.7068124

C	5.01704100	-0.64521100	-0.55071700
C	3.71270100	-0.79338700	0.02263900
C	3.55088100	-1.09800100	1.38634200
C	4.68681400	-1.25581700	2.16506600
C	5.97999500	-1.11692800	1.60950900
C	6.15065500	-0.81634200	0.26686800
C	4.80360700	-0.33869600	-1.92996000
C	3.42910500	-0.32320100	-2.09974900
H	2.56085800	-1.21219000	1.81960300
H	4.58344700	-1.49196100	3.22045700
H	6.84921600	-1.24900500	2.24790900
H	7.14966600	-0.71227800	-0.15051200
H	5.55260400	-0.16366300	-2.69059800
H	2.86905500	-0.12504300	-3.00849000
N	2.76734300	-0.60341700	-0.94447600
C	0.92191800	-0.44091000	-0.73579200
H	0.85390900	-1.26906000	-0.04274300
H	0.75372400	-0.64084400	-1.78672800
C	0.86897500	0.95695200	-0.22870600
C	0.94960100	2.03521200	-1.11657300
C	0.75849700	1.22299100	1.14065800
C	0.88531000	3.34623600	-0.65194300
H	1.05126600	1.84015100	-2.18151600
C	0.69758000	2.53346400	1.60774800
H	0.69283500	0.39372000	1.84025300
C	0.75488100	3.60376900	0.71345900
H	0.93758900	4.17006700	-1.35813700
H	0.60279200	2.71946600	2.67385000
H	0.70377900	4.62546000	1.07716900
Si	-2.46636000	-0.66949200	-0.48763800
C	-2.19210800	-1.94561600	-1.87462500
H	-1.43943000	-2.68037200	-1.56477100
H	-3.11286600	-2.47147800	-2.14404900
C	-2.49991300	1.18280200	-0.96808800
H	-3.50898600	1.57168900	-1.14437000
H	-1.90896800	1.32269500	-1.88135700
C	-1.99970700	-1.20739400	1.29135500
H	-1.69459100	-0.33546400	1.88330400
H	-2.82419700	-1.70649500	1.81313300
H	-0.72266300	-0.41688800	-0.63111400
O	-4.21490400	-0.97764000	-0.41583100
C	-5.24542500	-0.42247700	0.36804600
C	-4.76439800	0.58956300	1.42209500
C	-6.23660000	0.27217200	-0.57589500
C	-5.95425900	-1.58000900	1.08433400
H	-4.09556300	0.11803300	2.14688200

H	-4.23703800	1.42886100	0.96052100
H	-5.62617100	0.99001500	1.96790200
H	-6.58683500	-0.44124200	-1.32890000
H	-7.10353800	0.66468000	-0.03138600
H	-5.74500800	1.10201200	-1.09390200
H	-6.80719700	-1.22754900	1.67633200
H	-6.31256200	-2.30463700	0.34606200
H	-5.25135600	-2.08979000	1.75182900
H	-1.14433800	-1.89253900	1.26103100
H	-2.01429900	1.78965900	-0.19233000
H	-1.79918500	-1.44311900	-2.76684900

N-Benzylindole Radical Anion Fragmentation Transition State

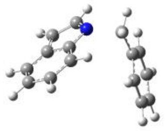

29

-633.9475339

C	-2.10272900	0.68365600	-0.69888300
C	-1.49111000	0.25735200	0.52269300
C	-1.83928700	-0.96551900	1.11809800
C	-2.79720800	-1.75432600	0.49737300
C	-3.41555000	-1.33925500	-0.70452900
C	-3.07827000	-0.13357600	-1.30115800
C	-1.50360700	1.94221400	-1.01638400
C	-0.59468200	2.19226400	-0.00378600
H	-1.36650400	-1.28946400	2.04151300
H	-3.07598600	-2.70710300	0.93893600
H	-4.16349400	-1.97871900	-1.16553400
H	-3.55931000	0.17494300	-2.22677100
H	-1.70985700	2.57152200	-1.87209700
H	0.07549900	3.03883300	0.10335500
N	-0.59447300	1.20887700	0.94922700
C	0.95468000	0.75892200	1.73620100
H	0.55404600	0.12816900	2.52831900
H	1.21520900	1.75859500	2.08534200
C	1.87683500	0.14215400	0.83688300
C	2.71173200	0.92655900	-0.03168700
C	1.85868200	-1.27231000	0.58905900
C	3.50014100	0.33668100	-1.00139700
H	2.72992400	2.00818500	0.09600100
C	2.65066400	-1.84597700	-0.38521200
H	1.20949100	-1.90118900	1.19650500
C	3.50102800	-1.06078100	-1.19855100

H	4.13159700	0.96650100	-1.62581400
H	2.61686200	-2.92436900	-0.52890500
H	4.11591600	-1.51639100	-1.96774800

Toluene Radical

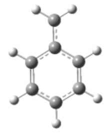

14
-270.8001515

C	0.00000	0.00000	-1.83684
C	0.00000	1.20985	-1.13292
C	0.00000	1.21516	0.25301
C	0.00000	0.00000	0.98908
C	0.00000	-1.21516	0.25301
C	0.00000	-1.20985	-1.13292
H	0.00000	0.00000	-2.92190
H	0.00000	2.15067	-1.67497
H	0.00000	2.15691	0.79526
H	0.00000	-2.15691	0.79526
H	0.00000	-2.15067	-1.67497
C	0.00000	0.00000	2.40194
H	0.00000	-0.93006	2.95759
H	0.00000	0.93006	2.95759

***N*-Benzylindole + [SiMe$_3$]$^{\bullet}$ Transition State**

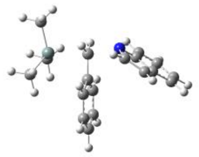

42
-1043.0014768

C	3.02288	-0.21448	-0.79201
C	2.00723	-1.04426	-0.24456
C	2.10681	-1.54835	1.05730
C	3.22891	-1.21428	1.80719
C	4.24695	-0.40344	1.26885
C	4.15765	0.09110	-0.02704
C	2.58218	0.11790	-2.12377
C	1.36898	-0.50075	-2.28568
H	1.33282	-2.19033	1.47105
H	3.32819	-1.59273	2.82015

H	5.11617	-0.16808	1.87556
H	4.95362	0.70554	-0.43920
H	3.11219	0.70815	-2.85923
H	0.71737	-0.47417	-3.15212
N	1.00842	-1.24890	-1.18265
C	-0.61238	0.56273	-0.12527
C	-0.75824	1.60435	-1.05845
C	-0.25338	0.89028	1.19457
C	-0.58440	2.92993	-0.67577
H	-1.01540	1.36540	-2.08772
C	-0.07921	2.21734	1.57289
H	-0.11227	0.09496	1.92182
C	-0.24765	3.24451	0.64206
H	-0.70669	3.72003	-1.41025
H	0.19587	2.45022	2.59704
H	-0.11060	4.27918	0.93932
Si	-3.02206	-0.76062	0.14288
C	-3.63697	-2.37861	-0.58695
C	-3.90783	0.72391	-0.57541
C	-3.04269	-0.78882	2.01709
H	-4.68189	-2.54695	-0.30188
H	-3.58283	-2.36501	-1.67970
H	-3.04609	-3.22527	-0.22452
H	-4.98437	0.63648	-0.38813
H	-3.54580	1.65024	-0.12026
H	-3.75499	0.79145	-1.65694
H	-2.36529	-1.55039	2.41595
H	-2.75549	0.18222	2.42899
H	-4.05558	-1.02552	2.36312
C	-0.75345	-0.84462	-0.54785
H	-0.69588	-1.64221	0.18803
H	-1.16565	-1.07896	-1.52611

$PhCH_2SiMe_3$

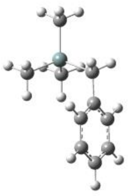

27

-680.0377210

C	3.45887	0.00000	0.42762
C	2.81572	1.20372	0.13962
C	1.54349	1.20144	-0.42946
C	0.88488	0.00000	-0.72397
C	1.54349	-1.20144	-0.42946

C	2.81572	-1.20372	0.13962
H	4.45097	0.00000	0.86800
H	3.30684	2.14816	0.35496
H	1.04944	2.14515	-0.65046
H	1.04945	-2.14515	-0.65046
H	3.30684	-2.14816	0.35496
C	-0.51008	0.00000	-1.28297
H	-0.67655	0.88316	-1.91165
H	-0.67655	-0.88316	-1.91165
Si	-1.82566	0.00000	0.09307
C	-3.52770	0.00000	-0.71162
H	-4.31791	0.00000	0.04648
H	-3.66446	0.88595	-1.34050
H	-3.66446	-0.88595	-1.34050
C	-1.61150	-1.53887	1.15384
H	-1.70578	-2.45012	0.55335
H	-0.62760	-1.55083	1.63425
H	-2.37298	-1.57296	1.94019
C	-1.61150	1.53887	1.15384
H	-0.62760	1.55083	1.63425
H	-1.70578	2.45012	0.55335
H	-2.37298	1.57296	1.94019

Indole Radical

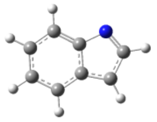

15
-363.0279562

C	0.26109	0.74139	-0.00010
C	0.29459	-0.68015	0.00010
C	-0.86703	-1.42390	0.00010
C	-2.09115	-0.72861	0.00003
C	-2.13758	0.66741	0.00006
C	-0.95974	1.42195	0.00004
C	1.62934	1.13997	-0.00033
C	2.38295	-0.09259	0.00054
H	-0.83386	-2.50848	-0.00001
H	-3.01944	-1.29119	-0.00003
H	-3.09836	1.17135	0.00006
H	-0.99699	2.50725	0.00003
H	2.03018	2.14489	-0.00019
H	3.46461	-0.16849	0.00107
N	1.62557	-1.16116	-0.00051

3-Methyl-*N*-benzylindole

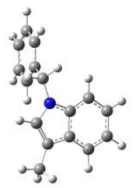

Optimised Geometry

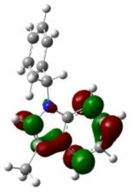

LUMO

32

-673.2471044

C	-2.28562	0.00296	0.11784
C	-1.07881	0.38249	-0.51921
C	-0.71143	1.72251	-0.69307
C	-1.59449	2.68804	-0.23202
C	-2.80969	2.33354	0.39175
C	-3.16077	1.00394	0.57169
C	-2.31951	-1.43673	0.14495
C	-1.16401	-1.85135	-0.46238
H	0.23049	1.99545	-1.16042
H	-1.34385	3.73771	-0.34926
H	-3.47455	3.11787	0.73964
H	-4.09538	0.73907	1.05900
H	-0.80409	-2.85615	-0.64520
N	-0.41461	-0.76651	-0.87990
C	0.90747	-0.81396	-1.46198
H	0.93402	-0.16427	-2.34475
H	1.07110	-1.83720	-1.81551
C	2.01198	-0.41498	-0.49905
C	1.82581	-0.46509	0.88338
C	3.24995	-0.01313	-1.00693
C	2.86787	-0.12522	1.74601
H	0.86047	-0.76270	1.28564
C	4.29275	0.32358	-0.14627
H	3.39775	0.03963	-2.08335
C	4.10378	0.26754	1.23474
H	2.71072	-0.16415	2.81950
H	5.24936	0.63705	-0.55302
H	4.91296	0.53485	1.90701
C	-3.41029	-2.28950	0.71453
H	-3.56520	-2.07831	1.77794
H	-4.36296	-2.11067	0.20450
H	-3.16860	-3.35054	0.61357

3-Methyl-*N*-benzylindole Radical Anion

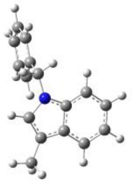

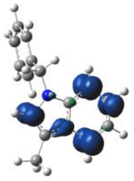

Optimised Geometry Spin Density

32

-673.2547066

C	-2.22467	-0.14811	0.13014
C	-1.14227	0.43937	-0.57839
C	-0.95191	1.82833	-0.69813
C	-1.93691	2.65817	-0.06399
C	-3.01658	2.10403	0.61691
C	-3.20846	0.71059	0.73586
C	-2.06345	-1.55744	0.05764
C	-0.91022	-1.81896	-0.67654
H	-0.08939	2.25123	-1.20361
H	-1.83744	3.73945	-0.12124
H	-3.74164	2.77535	1.07603
H	-4.05569	0.30074	1.27830
H	-0.45612	-2.74916	-0.98976
N	-0.37581	-0.58928	-1.08538
C	0.94366	-0.41046	-1.61946
H	0.93841	0.44258	-2.31026
H	1.19747	-1.29798	-2.21191
C	2.01265	-0.19440	-0.55824
C	1.72519	-0.36463	0.79797
C	3.31031	0.15687	-0.94067
C	2.72539	-0.18386	1.75475
H	0.71198	-0.62205	1.09556
C	4.31110	0.33367	0.01354
H	3.53689	0.29679	-1.99611
C	4.01922	0.16316	1.36776
H	2.48954	-0.31104	2.80728
H	5.31420	0.61077	-0.29790
H	4.79384	0.30514	2.11553
C	-2.99710	-2.56822	0.64892
H	-3.11199	-2.42259	1.73095
H	-4.00167	-2.49495	0.21139
H	-2.63347	-3.58790	0.48501

Anthracene

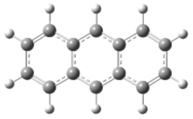

24

-539.3245036

C	3.65291	-0.71491	0.00002
C	2.47601	-1.40683	-0.00004
C	1.21949	-0.71862	-0.00004
C	1.21949	0.71862	-0.00002
C	2.47601	1.40683	0.00002
C	3.65291	0.71491	0.00006
C	0.00000	-1.40222	-0.00003
C	0.00000	1.40223	-0.00003
C	-1.21949	0.71862	-0.00002
C	-1.21949	-0.71862	-0.00001
C	-2.47601	-1.40683	0.00003
H	-2.47123	-2.49359	0.00007
C	-3.65291	-0.71491	0.00006
C	-3.65291	0.71491	0.00001
C	-2.47601	1.40683	-0.00003
H	-0.00001	-2.49025	-0.00004
H	4.59876	-1.24745	0.00005
H	2.47123	-2.49359	-0.00009
H	2.47123	2.49359	0.00003
H	4.59875	1.24745	0.00012
H	-0.00000	2.49025	-0.00004
H	-4.59875	-1.24745	0.00009
H	-4.59875	1.24745	0.00003
H	-2.47123	2.49359	-0.00007

Anthracene (Acetonitrile as solvent)

24

-539.3271692

C	3.65364	-0.71519	0.00001
C	2.47647	-1.40776	-0.00000
C	1.21967	-0.71898	-0.00001
C	1.21967	0.71898	-0.00001
C	2.47647	1.40776	0.00001
C	3.65364	0.71519	0.00002
C	0.00000	-1.40336	-0.00001
C	0.00000	1.40336	-0.00001
C	-1.21967	0.71898	-0.00001
C	-1.21967	-0.71898	-0.00001

C	-2.47647	-1.40776	0.00001
H	-2.47173	-2.49454	0.00002
C	-3.65364	-0.71519	0.00001
C	-3.65364	0.71519	0.00001
C	-2.47647	1.40776	-0.00001
H	-0.00000	-2.49138	-0.00001
H	4.59961	-1.24753	0.00002
H	2.47173	-2.49455	-0.00002
H	2.47173	2.49455	0.00001
H	4.59961	1.24753	0.00003
H	0.00000	2.49138	-0.00002
H	-4.59961	-1.24753	0.00002
H	-4.59961	1.24753	0.00001
H	-2.47173	2.49455	-0.00001

Anthracene Optimised Geometry - Single Point as Radical Anion

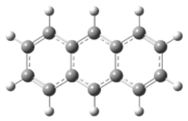

24

-539.3777390

C	3.65290900	-0.71491300	0.00002000
C	2.47600900	-1.40682800	-0.00004300
C	1.21949000	-0.71862400	-0.00003600
C	1.21949100	0.71862300	-0.00001900
C	2.47601000	1.40682800	0.00002100
C	3.65291000	0.71491100	0.00006200
C	0.00000000	-1.40222400	-0.00002900
C	0.00000100	1.40222500	-0.00002800
C	-1.21949000	0.71862400	-0.00002400
C	-1.21949100	-0.71862300	-0.00001000
C	-2.47600900	-1.40682800	0.00002900
H	-2.47123000	-2.49359200	0.00006800
C	-3.65290900	-0.71491100	0.00005700
C	-3.65290900	0.71491100	0.00000700
C	-2.47600900	1.40682800	-0.00002900
H	-0.00000600	-2.49024700	-0.00004100
H	4.59875700	-1.24744600	0.00005100
H	2.47122700	-2.49359200	-0.00009100
H	2.47122800	2.49359100	0.00002800
H	4.59875400	1.24745100	0.00012200
H	-0.00000400	2.49024700	-0.00004400
H	-4.59875500	-1.24745000	0.00008900
H	-4.59875500	1.24745000	0.00002500

H	-2.47123100	2.49359200	-0.00006800

Anthracene Radical Anion

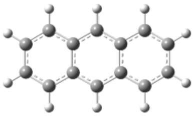

Optimised Geometry

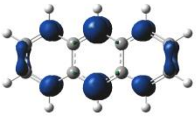

Spin Density

24

-539.3822999

C	3.69400	-0.70192	0.00000
C	2.48716	-1.39559	-0.00001
C	1.23864	-0.72336	0.00001
C	1.23864	0.72336	0.00000
C	2.48716	1.39559	-0.00001
C	3.69400	0.70192	0.00000
C	-0.00000	-1.40414	0.00001
C	0.00000	1.40414	0.00000
C	-1.23864	0.72336	-0.00001
C	-1.23864	-0.72336	-0.00001
C	-2.48716	-1.39559	-0.00002
H	-2.48439	-2.48406	-0.00001
C	-3.69400	-0.70192	-0.00000
C	-3.69400	0.70192	0.00001
C	-2.48716	1.39559	0.00000
H	-0.00001	-2.49312	0.00003
H	4.63421	-1.24728	0.00004
H	2.48439	-2.48406	0.00001
H	2.48439	2.48406	-0.00004
H	4.63420	1.24729	-0.00001
H	-0.00001	2.49312	0.00002
H	-4.63420	-1.24729	-0.00002
H	-4.63420	1.24729	0.00005
H	-2.48440	2.48406	-0.00001

Anthracene Radical Anion (Acetonitrile as solvent)

24

-539.4123390

C	-3.69532	-0.70222	0.00000
C	-2.48853	-1.39641	-0.00000
C	-1.23962	-0.72365	-0.00000
C	-1.23962	0.72365	0.00000
C	-2.48853	1.39641	0.00001
C	-3.69532	0.70222	0.00001

C	0.00000	-1.40508	-0.00001
C	0.00000	1.40508	-0.00001
C	1.23962	0.72365	-0.00000
C	1.23962	-0.72365	-0.00000
C	2.48853	-1.39641	0.00000
H	2.48723	-2.48488	0.00001
C	3.69532	-0.70222	0.00000
C	3.69532	0.70222	0.00000
C	2.48853	1.39641	-0.00000
H	0.00000	-2.49422	-0.00001
H	-4.63505	-1.24781	-0.00001
H	-2.48723	-2.48488	-0.00001
H	-2.48723	2.48488	0.00002
H	-4.63505	1.24781	0.00002
H	0.00000	2.49422	-0.00001
H	4.63505	-1.24781	0.00001
H	4.63505	1.24781	-0.00000
H	2.48723	2.48488	0.00000

Anthracene Radical Anion Optimised Geometry - Single Point as Neutral

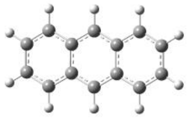

24

-539.3199533

C	3.69399900	-0.70192300	0.00000200
C	2.48715700	-1.39558500	-0.00000700
C	1.23863900	-0.72335800	0.00000800
C	1.23863800	0.72335800	0.00000100
C	2.48715700	1.39558500	-0.00001000
C	3.69399800	0.70192100	0.00000200
C	-0.00000100	-1.40414200	0.00001100
C	0.00000100	1.40414300	0.00000400
C	-1.23863700	0.72336000	-0.00000800
C	-1.23863700	-0.72335800	-0.00000500
C	-2.48715700	-1.39558600	-0.00001800
H	-2.48439300	-2.48405700	-0.00000800
C	-3.69399700	-0.70192300	-0.00000300
C	-3.69399800	0.70192200	0.00001200
C	-2.48715800	1.39558500	0.00000200
H	-0.00000700	-2.49312000	0.00002900
H	4.63420600	-1.24728100	0.00003600
H	2.48439100	-2.48405600	0.00000600
H	2.48439000	2.48405600	-0.00003700

H	4.63419900	1.24729000	-0.00001400
H	-0.00000700	2.49312100	0.00002400
H	-4.63420100	-1.24728800	-0.00002100
H	-4.63420200	1.24728600	0.00004500
H	-2.48439600	2.48405600	-0.00001200

9,10-Dihydroanthracen-9-ide

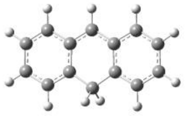

25

-539.9805637

C	-3.69995	0.73094	-0.00100
C	-2.49141	1.41166	-0.00003
C	-1.23528	0.73793	0.00070
C	-1.28444	-0.69249	0.00049
C	-2.50678	-1.35021	-0.00061
C	-3.73236	-0.66873	-0.00138
C	-0.00000	1.43033	0.00146
C	0.00000	-1.50936	0.00222
C	1.28444	-0.69249	0.00049
C	1.23528	0.73794	0.00069
C	2.49141	1.41166	-0.00011
H	2.48753	2.50048	-0.00000
C	3.69995	0.73094	-0.00104
C	3.73236	-0.66873	-0.00133
C	2.50678	-1.35021	-0.00055
H	-0.00000	2.51763	0.00136
H	-4.62977	1.29678	-0.00156
H	-2.48753	2.50048	0.00012
H	-2.50483	-2.44122	-0.00075
H	-4.67248	-1.21135	-0.00220
H	0.00001	-2.17909	0.87704
H	4.62977	1.29678	-0.00163
H	4.67249	-1.21134	-0.00208
H	2.50483	-2.44122	-0.00067
H	-0.00001	-2.18286	-0.86965

Anthracene + [(Me)$_3$(H)SiO^{t}Bu]$^-$ Transition State

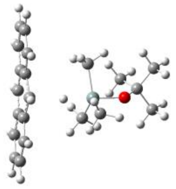

52

-1182.1182990

C	-3.00674	-3.42561	-0.87824
C	-2.92816	-2.23193	-1.54654
C	-2.61805	-1.01522	-0.86099
C	-2.39090	-1.08263	0.55169
C	-2.47716	-2.33664	1.21239
C	-2.78030	-3.48625	0.52578
C	-2.53949	0.21904	-1.52478
C	-1.99511	0.08967	1.24737
C	-2.10431	1.35273	0.60936
C	-2.31882	1.40490	-0.80717
C	-2.32129	2.69116	-1.43547
H	-2.47245	2.74020	-2.51149
C	-2.13947	3.83820	-0.70817
C	-1.94280	3.78060	0.70043
C	-1.92312	2.56176	1.33083
H	-2.70768	0.26330	-2.59847
H	-3.23928	-4.33683	-1.42240
H	-3.09524	-2.19175	-2.62054
H	-2.27487	-2.36969	2.28112
H	-2.83758	-4.43909	1.04293
H	-1.95119	0.05902	2.33193
H	-2.14375	4.80250	-1.20916
H	-1.79460	4.69672	1.26357
H	-1.74131	2.49862	2.40178
Si	1.51568	-0.27227	0.89534
C	1.81309	-0.95855	2.65716
H	1.16438	-1.82753	2.82298
H	2.85317	-1.24842	2.83727
C	1.38747	1.63201	0.67063
H	2.33569	2.11930	0.41685
H	1.01151	2.06736	1.60468
C	0.86336	-1.48223	-0.44380
H	0.32867	-0.92079	-1.22197
H	1.65680	-2.07335	-0.91622
H	-0.12942	-0.13736	1.34532
O	3.27571	-0.42812	0.50496
C	4.02135	-0.12776	-0.64690
C	3.18305	0.34146	-1.85062

C	5.03261	0.97356	-0.29065
C	4.78716	-1.39672	-1.05096
H	2.51026	-0.44717	-2.19694
H	2.58304	1.22059	-1.59949
H	3.84736	0.60851	-2.68089
H	5.63217	0.65443	0.56827
H	5.70523	1.19320	-1.12887
H	4.50696	1.89404	-0.01647
H	5.43044	-1.22398	-1.92254
H	5.40872	-1.73207	-0.21404
H	4.07729	-2.19544	-1.29117
H	0.12804	-2.16583	-0.00201
H	0.64562	1.87889	-0.10118
H	1.52252	-0.19983	3.39458

Phenanthrene

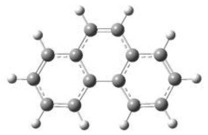

24

-539.3352007

C	3.55438	-0.29589	0.00007
C	2.83173	0.87783	0.00020
C	1.41864	0.86130	0.00012
C	0.72936	-0.37772	-0.00003
C	1.49532	-1.56606	-0.00024
C	2.87544	-1.52857	-0.00019
C	0.67759	2.09462	0.00008
C	-0.72936	-0.37772	0.00001
C	-1.41864	0.86130	-0.00012
C	-0.67760	2.09462	-0.00011
C	-2.83173	0.87783	-0.00016
H	-3.34095	1.83812	-0.00029
C	-3.55438	-0.29589	-0.00003
C	-2.87544	-1.52858	0.00020
C	-1.49532	-1.56607	0.00022
H	1.23293	3.02864	0.00016
H	4.63942	-0.27214	0.00014
H	3.34094	1.83812	0.00036
H	1.00181	-2.53133	-0.00053
H	3.43898	-2.45628	-0.00039
H	-1.23292	3.02864	-0.00022
H	-4.63942	-0.27215	-0.00005
H	-3.43899	-2.45628	0.00038

H	-1.00179	-2.53132	0.00047

Phenanthrene (Acetonitrile as solvent)

24

-539.3378926

C	3.55525	-0.29589	0.00004
C	2.83251	0.87849	0.00012
C	1.41898	0.86164	0.00007
C	0.72970	-0.37814	-0.00002
C	1.49562	-1.56710	-0.00016
C	2.87631	-1.52911	-0.00012
C	0.67781	2.09562	0.00006
C	-0.72971	-0.37813	0.00001
C	-1.41898	0.86164	-0.00007
C	-0.67781	2.09562	-0.00006
C	-2.83252	0.87849	-0.00011
H	-3.34186	1.83872	-0.00019
C	-3.55525	-0.29589	-0.00003
C	-2.87631	-1.52911	0.00012
C	-1.49562	-1.56710	0.00015
H	1.23302	3.02970	0.00011
H	4.64026	-0.27189	0.00008
H	3.34186	1.83872	0.00021
H	1.00290	-2.53282	-0.00034
H	3.43977	-2.45682	-0.00024
H	-1.23302	3.02970	-0.00012
H	-4.64026	-0.27189	-0.00005
H	-3.43977	-2.45682	0.00024
H	-1.00290	-2.53282	0.00032

Phenanthrene Optimised Geometry - Single Point as Radical Anion

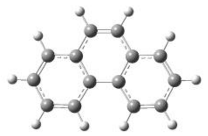

24

-539.3643171

C	3.55437900	-0.29588900	0.00007100
C	2.83172700	0.87783200	0.00020200
C	1.41864300	0.86130300	0.00011500
C	0.72936000	-0.37772500	-0.00002800
C	1.49531700	-1.56606400	-0.00024100
C	2.87543800	-1.52857400	-0.00019400
C	0.67759500	2.09461700	0.00007600
C	-0.72936200	-0.37772200	0.00000500

C	-1.41864100	0.86130200	-0.00012300
C	-0.67759600	2.09461600	-0.00011000
C	-2.83172900	0.87783300	-0.00016300
H	-3.34094600	1.83811900	-0.00029300
C	-3.55437800	-0.29588800	-0.00002800
C	-2.87543600	-1.52857700	0.00019700
C	-1.49531800	-1.56606600	0.00021700
H	1.23292800	3.02864100	0.00016300
H	4.63941700	-0.27214300	0.00013800
H	3.34094400	1.83811900	0.00036000
H	1.00180500	-2.53132900	-0.00053100
H	3.43898400	-2.45628000	-0.00038800
H	-1.23292400	3.02864300	-0.00022000
H	-4.63941600	-0.27214800	-0.00005400
H	-3.43899200	-2.45627600	0.00038100
H	-1.00179400	-2.53132500	0.00047500

Phenanthrene Radical Anion

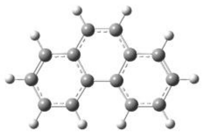

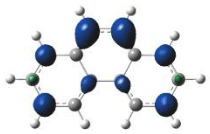

Optimised Geometry

Spin Density

24

-539.3710952

C	3.57188	-0.31503	0.00015
C	2.86283	0.86960	0.00052
C	1.42940	0.89353	0.00026
C	0.74003	-0.37575	-0.00010
C	1.49738	-1.55073	-0.00065
C	2.89931	-1.54833	-0.00052
C	0.70389	2.09783	0.00021
C	-0.74003	-0.37575	0.00009
C	-1.42940	0.89354	-0.00026
C	-0.70389	2.09783	-0.00024
C	-2.86283	0.86959	-0.00049
H	-3.39049	1.82152	-0.00090
C	-3.57188	-0.31503	-0.00012
C	-2.89931	-1.54834	0.00052
C	-1.49738	-1.55073	0.00064
H	1.25262	3.03715	0.00041
H	4.65984	-0.29012	0.00033
H	3.39049	1.82152	0.00097
H	0.99094	-2.51173	-0.00126
H	3.45036	-2.48350	-0.00098

H	-1.25262	3.03715	-0.00045
H	-4.65984	-0.29013	-0.00028
H	-3.45035	-2.48351	0.00096
H	-0.99093	-2.51173	0.00124

Phenanthrene Radical Anion (Acetonitrile as solvent)

24

-539.4014840

C	3.57293	-0.31568	0.00013
C	2.86405	0.86991	0.00047
C	1.43041	0.89411	0.00024
C	0.74044	-0.37565	-0.00010
C	1.49776	-1.55189	-0.00060
C	2.89962	-1.54911	-0.00048
C	0.70459	2.09971	0.00021
C	-0.74044	-0.37565	0.00009
C	-1.43041	0.89411	-0.00024
C	-0.70459	2.09971	-0.00022
C	-2.86405	0.86991	-0.00045
H	-3.39271	1.82114	-0.00084
C	-3.57292	-0.31568	-0.00012
C	-2.89962	-1.54912	0.00048
C	-1.49776	-1.55190	0.00060
H	1.25310	3.03910	0.00040
H	4.66041	-0.29105	0.00030
H	3.39271	1.82114	0.00087
H	0.99221	-2.51338	-0.00116
H	3.45020	-2.48418	-0.00089
H	-1.25310	3.03910	-0.00041
H	-4.66041	-0.29106	-0.00028
H	-3.45020	-2.48419	0.00088
H	-0.99221	-2.51338	0.00114

Phenanthrene Radical Anion Optimised Geometry - Single Point as Neutral

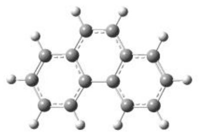

24

-539.3283940

C	3.57188400	-0.31502700	0.00014800
C	2.86282900	0.86959700	0.00051700
C	1.42939700	0.89353500	0.00025900
C	0.74003500	-0.37575200	-0.00009900
C	1.49738500	-1.55073300	-0.00064900

C	2.89931200	-1.54833100	-0.00052500
C	0.70388600	2.09782900	0.00021400
C	-0.74003400	-0.37574800	0.00009000
C	-1.42939700	0.89353600	-0.00026400
C	-0.70389200	2.09783000	-0.00024400
C	-2.86283300	0.86959200	-0.00048600
H	-3.39049100	1.82152100	-0.00090100
C	-3.57188400	-0.31502800	-0.00012400
C	-2.89930600	-1.54833700	0.00052100
C	-1.49738500	-1.55073300	0.00063700
H	1.25261700	3.03714800	0.00041400
H	4.65983800	-0.29012300	0.00033000
H	3.39048900	1.82152400	0.00096900
H	0.99093600	-2.51173500	-0.00125600
H	3.45035900	-2.48350100	-0.00098100
H	-1.25262300	3.03714900	-0.00044800
H	-4.65983700	-0.29013000	-0.00028500
H	-3.45035400	-2.48350500	0.00095700
H	-0.99092900	-2.51173200	0.00123800

9,10-Dihydrophenanthren-9-ide

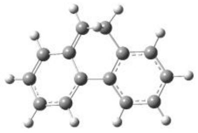

25
-539.9662745

C	3.53346	-0.38339	-0.22064
C	2.82446	0.80620	-0.05156
C	1.44164	0.81729	0.13570
C	0.71383	-0.40064	0.11978
C	1.44552	-1.59224	-0.07499
C	2.82803	-1.58960	-0.22287
C	0.70467	2.10232	0.44325
C	-0.74707	-0.36583	0.18568
C	-1.39536	0.90741	-0.11915
C	-0.67396	2.08102	-0.15985
C	-2.81777	0.83102	-0.38457
H	-3.32796	1.74506	-0.68542
C	-3.53208	-0.32935	-0.22321
C	-2.90239	-1.53505	0.18321
C	-1.52272	-1.52133	0.35308
H	0.66416	2.20962	1.55116
H	4.61162	-0.37026	-0.35200

H	3.35812	1.75547	-0.04436
H	0.91700	-2.53891	-0.13839
H	3.35342	-2.53050	-0.36619
H	-1.16713	3.01958	-0.40064
H	-4.60604	-0.32014	-0.40382
H	-3.47710	-2.44175	0.34164
H	-1.02452	-2.45024	0.62707
H	1.29701	2.95505	0.08777

Phenanthrene + [(Me)$_3$(H)SiO^{t}Bu]$^-$ Transition State

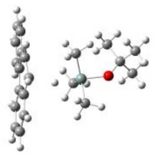

52

-1182.1229112

C	-2.81793	-3.35800	-0.55202
C	-2.16367	-2.75758	0.50797
C	-2.10249	-1.35758	0.63283
C	-2.71376	-0.53926	-0.34497
C	-3.38499	-1.17359	-1.41350
C	-3.43608	-2.55207	-1.52158
C	-1.36721	-0.75950	1.74646
C	-2.64637	0.91646	-0.21053
C	-2.07153	1.45719	0.97856
C	-1.53118	0.60097	1.97819
C	-2.06667	2.86945	1.13760
H	-1.63529	3.28286	2.04656
C	-2.57742	3.70821	0.16881
C	-3.12016	3.17220	-1.01416
C	-3.14882	1.79755	-1.18654
H	-1.14068	-1.41384	2.58253
H	-2.85022	-4.44014	-0.63683
H	-1.65995	-3.36375	1.25794
H	-3.87863	-0.57627	-2.17274
H	-3.95601	-3.00890	-2.35864
H	-1.13530	1.05022	2.88459
H	-2.55086	4.78474	0.31534
H	-3.50881	3.82784	-1.78703
H	-3.56520	1.40063	-2.10715
Si	1.77913	-0.85541	0.52028
C	2.10434	-2.71685	0.79892
H	1.36826	-3.30361	0.23603
H	3.10992	-3.02151	0.49153
C	1.96152	0.29933	2.04060

H	2.96884	0.71332	2.15950
H	1.70700	-0.26860	2.94413
C	0.74706	-0.34116	-1.01264
H	0.12843	0.53359	-0.77319
H	1.35086	-0.11656	-1.89919
H	0.20883	-1.13579	1.20157
O	3.41351	-0.60792	-0.17693
C	4.05083	0.52929	-0.70526
C	3.22275	1.82436	-0.62199
C	5.35918	0.73320	0.07216
C	4.38084	0.24552	-2.17834
H	2.28561	1.73751	-1.17800
H	2.98529	2.07724	0.41486
H	3.79530	2.65494	-1.05102
H	5.95986	-0.18090	0.02367
H	5.94703	1.56515	-0.33429
H	5.13853	0.94219	1.12440
H	4.93706	1.07256	-2.63590
H	4.98290	-0.66656	-2.24835
H	3.45746	0.08913	-2.74574
H	0.05243	-1.15338	-1.26036
H	1.24638	1.12919	1.97486
H	1.96573	-2.96101	1.85897

Naphthalene

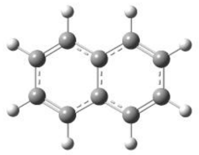

18

-385.7427218

C	2.42934	0.70923	0.00000
C	1.24343	1.40134	-0.00000
C	0.00001	0.71211	-0.00000
C	-0.00001	-0.71211	-0.00000
C	1.24342	-1.40134	-0.00000
C	2.42933	-0.70925	0.00000
H	-1.23821	2.48823	-0.00001
H	3.37304	1.24565	0.00000
H	1.23824	2.48823	0.00000
C	-1.24343	1.40135	0.00000
C	-1.24344	-1.40135	0.00000
H	1.23822	-2.48823	0.00000
H	3.37303	-1.24567	0.00001
C	-2.42933	-0.70924	0.00000

C	-2.42932	0.70925	0.00000
H	-1.23823	-2.48823	-0.00000
H	-3.37304	-1.24564	0.00000
H	-3.37303	1.24566	0.00000

Naphthalene (Acetonitrile as solvent)

18

-385.7447592

C	-2.43005	-0.70953	0.00000
C	-1.24382	-1.40223	0.00000
C	-0.00000	-0.71249	0.00000
C	0.00000	0.71249	0.00000
C	-1.24382	1.40223	0.00000
C	-2.43004	0.70953	0.00000
H	1.23872	-2.48914	-0.00000
H	-3.37385	-1.24581	0.00000
H	-1.23872	-2.48914	0.00000
C	1.24382	-1.40223	-0.00000
C	1.24383	1.40223	-0.00000
H	-1.23872	2.48914	0.00000
H	-3.37385	1.24581	0.00000
C	2.43004	0.70953	0.00000
C	2.43004	-0.70953	-0.00000
H	1.23872	2.48914	-0.00000
H	3.37385	1.24580	0.00000
H	3.37385	-1.24581	-0.00000

Naphthalene Optimised Geometry - Single Point as Radical Anion

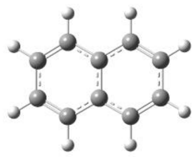

18

-385.7692108

C	2.42933500	0.70923400	0.00000100
C	1.24343300	1.40133900	-0.00000200
C	0.00001100	0.71211000	-0.00000100
C	-0.00000900	-0.71210800	-0.00000100
C	1.24342200	-1.40134100	-0.00000200
C	2.42932600	-0.70924600	0.00000200
H	-1.23820800	2.48823500	-0.00000500
H	3.37303800	1.24565500	0.00000400
H	1.23823700	2.48822600	0.00000000
C	-1.24342700	1.40134700	0.00000000
C	-1.24343900	-1.40134600	0.00000000

H	1.23822000	-2.48823000	0.00000100
H	3.37302800	-1.24566900	0.00000500
C	-2.42933100	-0.70923800	0.00000000
C	-2.42932300	0.70924800	0.00000200
H	-1.23822900	-2.48823100	-0.00000400
H	-3.37304400	-1.24564200	0.00000300
H	-3.37303000	1.24566200	0.00000200

Naphthalene Radical Anion

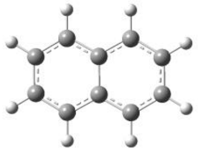

Optimised Geometry

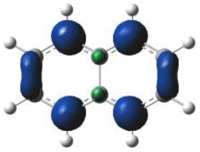

Spin Density

18

-385.7745588

C	2.46742	0.69503	0.00000
C	1.24830	1.40104	-0.00000
C	-0.00003	0.72441	-0.00000
C	0.00003	-0.72440	-0.00000
C	1.24826	-1.40101	-0.00000
C	2.46747	-0.69505	0.00000
H	-1.24711	2.48924	0.00000
H	3.40807	1.24139	0.00001
H	1.24717	2.48925	-0.00000
C	-1.24827	1.40102	0.00000
C	-1.24830	-1.40104	-0.00000
H	1.24712	-2.48924	-0.00000
H	3.40811	-1.24139	0.00000
C	-2.46741	-0.69503	0.00000
C	-2.46747	0.69504	0.00000
H	-1.24715	-2.48925	0.00001
H	-3.40806	-1.24139	0.00001
H	-3.40812	1.24138	-0.00000

Naphthalene Radical Anion (Acetonitrile as solvent)

18

-385.8064555

C	2.46889	0.69530	0.00000
C	1.24909	1.40152	-0.00000
C	-0.00002	0.72487	0.00000
C	0.00002	-0.72487	-0.00000
C	1.24908	-1.40151	0.00000
C	2.46892	-0.69530	0.00000
H	-1.24913	2.48975	0.00000

H	3.40895	1.24203	0.00000
H	1.24915	2.48976	-0.00000
C	-1.24908	1.40151	0.00000
C	-1.24909	-1.40152	0.00000
H	1.24913	-2.48975	0.00000
H	3.40898	-1.24202	0.00000
C	-2.46889	-0.69530	0.00000
C	-2.46892	0.69530	0.00000
H	-1.24915	-2.48976	0.00000
H	-3.40896	-1.24202	0.00000
H	-3.40898	1.24201	-0.00000

Naphthalene Radical Anion Optimised Geometry - Single Point as Neutral

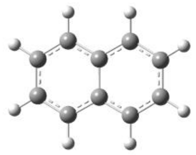

18
-385.7373307

C	2.46742200	0.69503100	0.00000300
C	1.24829900	1.40103900	-0.00000300
C	-0.00002800	0.72440600	-0.00000200
C	0.00002500	-0.72439900	-0.00000100
C	1.24826500	-1.40101500	-0.00000200
C	2.46747000	-0.69504800	0.00000100
H	-1.24710800	2.48923700	0.00000000
H	3.40806800	1.24139200	0.00000700
H	1.24717000	2.48925300	-0.00000300
C	-1.24827200	1.40101600	0.00000100
C	-1.24829900	-1.40103700	-0.00000200
H	1.24711700	-2.48923700	-0.00000200
H	3.40811400	-1.24138900	0.00000400
C	-2.46741500	-0.69503500	0.00000100
C	-2.46747100	0.69504400	0.00000200
H	-1.24715400	-2.48925100	0.00000500
H	-3.40806400	-1.24139300	0.00000700
H	-3.40811700	1.24138200	-0.00000200

1,4-Dihydronaphthalen-1-ide

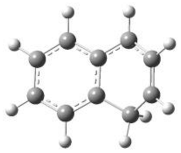

19
-386.3714755

C	-2.43115	-0.62005	-0.28072
C	-1.29428	-1.37323	0.36556
C	0.02312	-0.68114	0.08783
C	0.03079	0.75322	0.14650
C	-1.20642	1.45481	0.17221
C	-2.37511	0.74560	-0.23368
H	1.16332	-2.47116	-0.15301
H	-3.30435	-1.15019	-0.64990
H	-1.43836	-1.39497	1.47041
C	1.20743	-1.38288	-0.09921
C	1.30420	1.38568	0.08249
H	-1.19343	2.54142	0.14146
H	-3.23811	1.32489	-0.56852
C	2.47763	0.65932	-0.06553
C	2.45006	-0.73825	-0.17637
H	1.34627	2.47281	0.13497
H	3.42887	1.18681	-0.11039
H	3.36646	-1.30725	-0.30177
H	-1.24833	-2.42088	0.04227

Naphthalene + [(Me)$_3$(H)SiO^{t}Bu]$^-$ Transition State

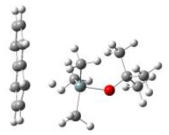

46
-1028.5276082

C	-2.31737	2.37200	-0.29171
C	-2.06982	1.22051	-1.06309
C	-2.64040	-0.03048	-0.60056
C	-3.17631	-0.12186	0.71524
C	-3.23902	1.04328	1.53297
C	-2.83572	2.25804	1.00909
H	-2.16962	-1.10037	-2.40720
H	-2.03807	3.34782	-0.67682
H	-1.91681	1.32519	-2.13305
C	-2.60616	-1.18331	-1.41347
C	-3.65886	-1.38344	1.15694
H	-3.65053	0.96971	2.53566
H	-2.94430	3.15883	1.61032
C	-3.61175	-2.49493	0.34407
C	-3.08007	-2.39888	-0.96158
H	-4.06606	-1.45957	2.16302

H	-3.98224	-3.44948	0.70789
H	-3.03871	-3.27720	-1.59877
Si	1.21758	0.65947	-0.50352
C	1.34846	2.54343	-0.76893
H	0.49381	3.04212	-0.29576
H	2.27653	2.96754	-0.37353
C	1.36955	-0.46991	-2.05454
H	2.39601	-0.78117	-2.28377
H	0.96613	0.06789	-2.92121
C	0.37852	0.05525	1.11127
H	-0.16929	-0.87962	0.93147
H	1.07579	-0.10060	1.94281
H	-0.40843	0.85656	-1.01707
O	2.92924	0.54452	0.03339
C	3.71004	-0.53764	0.47564
C	2.99514	-1.90054	0.43660
C	4.95599	-0.61272	-0.41942
C	4.14633	-0.24983	1.92001
H	2.10061	-1.90040	1.06508
H	2.70086	-2.16419	-0.58284
H	3.67055	-2.68064	0.80684
H	5.48465	0.34581	-0.39324
H	5.64204	-1.40382	-0.09321
H	4.65932	-0.81149	-1.45481
H	4.82067	-1.02564	2.30246
H	4.66085	0.71593	1.96049
H	3.26897	-0.19666	2.57284
H	-0.37019	0.79924	1.40810
H	0.75295	-1.36865	-1.92132
H	1.29320	2.76035	-1.84343
